# Benchmark analysis of algorithms for determining and quantifying full-length mRNA splice forms from RNA-seq data

**DOI:** 10.1093/bioinformatics/btv488

**Published:** 2015-09-03

**Authors:** Katharina E. Hayer, Angel Pizarro, Nicholas F. Lahens, John B. Hogenesch, Gregory R. Grant

**Affiliations:** ^1^University of Pennsylvania, Institute for Translational Medicine and Therapeutics, Philadelphia, PA 19104,; ^2^Scientific Computing at Amazon Web Services, Seattle, WA 98108,; ^3^Department of Pharmacology and; ^4^Department of Genetics, University of Pennsylvania, Philadelphia, PA 19104, USA

## Abstract

**Motivation:** Because of the advantages of RNA sequencing (RNA-Seq) over microarrays, it is gaining widespread popularity for highly parallel gene expression analysis. For example, RNA-Seq is expected to be able to provide accurate identification and quantification of full-length splice forms. A number of informatics packages have been developed for this purpose, but short reads make it a difficult problem in principle. Sequencing error and polymorphisms add further complications. It has become necessary to perform studies to determine which algorithms perform best and which if any algorithms perform adequately. However, there is a dearth of independent and unbiased benchmarking studies. Here we take an approach using both simulated and experimental benchmark data to evaluate their accuracy.

**Results:** We conclude that most methods are inaccurate even using idealized data, and that no method is highly accurate once multiple splice forms, polymorphisms, intron signal, sequencing errors, alignment errors, annotation errors and other complicating factors are present. These results point to the pressing need for further algorithm development.

**Availability and implementation:** Simulated datasets and other supporting information can be found at http://bioinf.itmat.upenn.edu/BEERS/bp2

**Supplementary information:** Supplementary data are available at *Bioinformatics* online.

**Contact:** hayer@upenn.edu

## 1 Introduction

We first fix some terminology. For our purposes a *gene* is a collection of transcripts, also called splice forms. A *transcript* is a collection of exons. An *exon* is a contiguous span of genomic coordinates. Two splice forms of the same gene can, and usually do, have some of the same exons. Exons which overlap but have different start and/or end location are also possible. Two different splice forms may share all of their exons, as long as at least one of them differs by their start/end coordinates. Typically for any given gene in any given cell, some of its splice forms are expressed and others are absent.

**Fig. 1.**
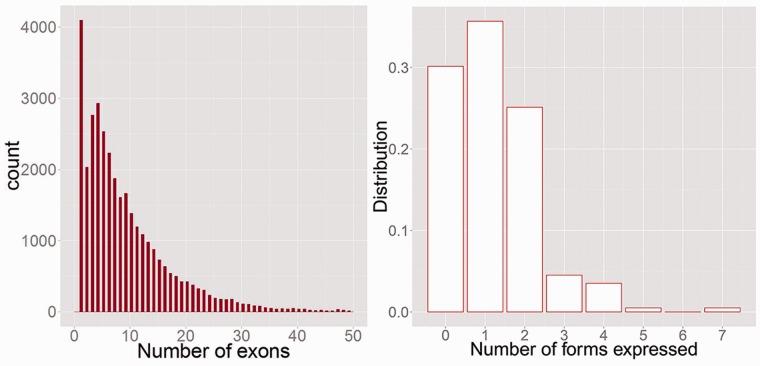
*Left*: Shows number of mouse mm9 ENSEMBL transcripts as a function of the number of exons. 90% of transcripts have multiple exons. 65% have >5 and 35% have >10. *Right:* Distribution of the minimum number of splice forms necessary to explain the RNA-Seq junctions in 300 M read pairs of mouse Liver (Zhang *et al.*, 2014). This is based on the first 200 RefSeq genes annotated on Chromosome 1

One of the primary goals of high throughput RNA-Sequencing (RNA-Seq) is to accurately identify the full-length structure of the transcripts that are present, and their relative abundances, so that researchers can focus on the most relevant splice forms in their system of interest. This is a very difficult problem however. Most human and mouse genes have many exons (Fig. 1, left) and are annotated with multiple splice forms. We observe that ∼35% of genes may express at least two forms under normal conditions (see Fig. 1, right). As methods improve and the number of tissues that are deep sequenced increases, the annotation will only get more complex. Moreover, for species without genome sequence available, *de novo* RNA-Seq and transcript assembly should provide an efficient means of gene discovery.

**Fig. 2.**
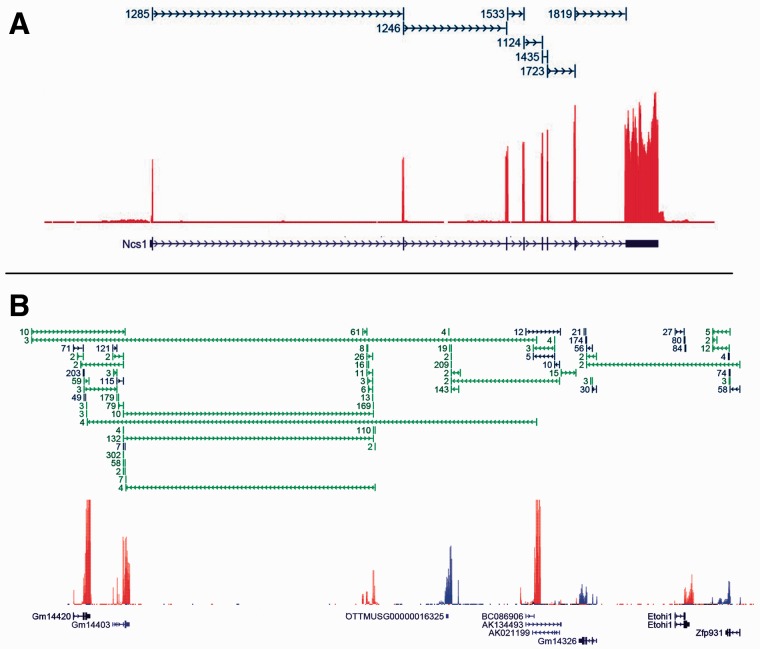
These plots depict mouse liver RNA-Seq data (Zhang *et al.,* 2014). Each plot has three tracks: transcript models (bottom), depth-of-coverage (middle, red = forward, blue = reverse) and spliced reads (top, blue = annotated, green = novel, numbers give how many reads spliced cleanly across each junction). **(A**) Shows data for a gene with one annotated splice form. In this case the one annotated splice form is sufficient to completely explain the data. (**B)** A region showing several annotated genes. Here there are many reads spliced between different genes. In addition to unannotated splice junctions, there is also evidence for completely unannotated genes in this region

**Fig. 3.**
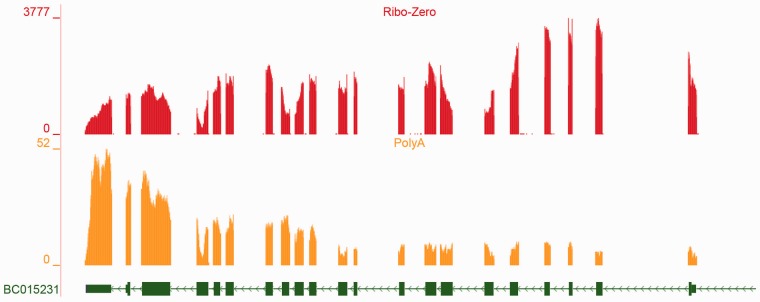
This shows the depth of coverage of a full-length cDNA clone, which has been transcribed and subjected to the Ribo-Zero (red) and PolyA selection (orange) protocols for removal of ribosomal RNA. Both protocols result in extreme local bias (Lahens *et al.,* 2014). PolyA causes 3′ bias (note this gene is oriented on the reverse strand)

By visually inspecting depth-of-coverage plots and representations of spliced reads in a genome browser, it is easy to see that a majority of genes are tractable and the expressed splice forms can be determined (Fig. 2A). Here the data can be explained completely by the one annotated splice form. Blue junctions indicate the existence of reads which mapped cleanly across an annotated splice junction (‘clean’ here means uniquely and with at least eight bases on each side). Note that the blue directional spans above the coverage plot indicate single reads (not read-pairs) spliced across intron-sized gaps. In contrast, Figure 2B shows a region with a significant number of reads spliced from one gene to another, as well as entirely unannotated genes. Green junctions indicate the existence of reads which mapped cleanly across *unannotated* junctions. A number of such genes and regions as shown in Figure 2B typically occur in every RNA-Seq experiment and present the challenging cases for transcript level analysis. The nature of current RNA-Seq, resulting in short error prone reads, alignment artifacts, bias introduced by the library construction process etc., introduces noise into this already difficult problem. For example the ribosomal depletion protocols invariably cause a severe deviation of signal from uniform across a single transcript. Figure 3 shows the result of sequencing a single full length cDNA clone after reverse transcription followed by Ribo-Zero (top) and polyA selection (bottom) (Lahens *et al.* 2014). In both cases the deviation from uniform is marked. At the present time there is no known protocol that doesn’t suffer from this kind of issue. Most algorithms assume at least reasonably uniform coverage across each expressed transcript.

Despite these challenges, many algorithms have been developed to infer full-length transcripts *de novo* from short read RNA-Seq data, of which Cufflinks (Trapnell *et al.,* 2010) is the most widely used. Other methods are StringTie (Pertea *et al.* 2015), Scripture (Guttman *et al.,* 2010), Trinity (Grabherr *et al.,* 2011), Oases (Velvet) (Schultz *et al.,* 2012), Soap-denovo-trans (Xie *et al*., 2014), iReckon (Mezlini *et al.,* 2013), CLASS (Song *et al*., 2013), FlipFlop (Bernard *et al.,* 2014), IsoLasso (Li *et al.,* 2011), MiTie (Behr *et al.,* 2013), Trans-ABySS (Robertson *et al.,* 2010), AUGUSTUS (Stanke *et al.,* 2008), Traph (Tomescu *et al.,* 2014).

Algorithms are classified by whether they use an alignment to a reference genome sequence, or whether they assemble the reads into full transcripts *de novo* by identifying overlapping reads. We call the former type *alignment guided* methods and the latter *de novo* methods. Methods can be run in one or both of two modes, depending on whether or not they utilize pre-existing annotation as a guide. We investigate the accuracy of both types of algorithms, genome guided and *de novo*, in both modes, wherever possible.

There is another class of algorithm that does not attempt to infer transcript structure, but instead just quantifies a set of given transcripts. It was not the goal of this study to evaluate this last category; however, the simulated data we provide could easily be used for that purpose.

A critical aspect of algorithm development is unbiased benchmarking. A few studies have investigated algorithms for transcript inference and quantification from RNA-Seq data, the most notable being the RGASP1 (2009) and RGASP2 (2010) competitions (Steijger *et al.,* 2013). These studies used a PCR approach to validate differences rather than simulated or spike-in data. Their overall conclusion was that ‘assembly of complete isoform structures poses a major challenge even when all constituent elements are identified’ and ‘[c]onsequently, the complexity of higher eukaryotic genomes imposes severe limitations on transcript recall and splice product discrimination’. They further identified that the alignment step that precedes most reconstruction algorithms greatly affects the results, so the following year RGASP3 was held solely to assess alignment algorithms (Engstrom *et al.,* 2013).

Though Steijger *et al.* was published in 2013 from the RGASP effort, it is based on analysis performed in 2009 and 2010 by participants in the first two RGASP competitions. Since then, there has been considerable progress in algorithm development, while few new articles have been published that analyze performance. Chandramohan *et al.* (2013) published an analysis, but the focus was on quantification, not transcript structure. For validation, they used ∼500 genes measured by RT-PCR (Nolan *et al.,* 2006). However, they relied on early generation public data using single-end 36-base reads. Read lengths continue to grow, with the current standard upwards of 100 bases. Furthermore, paired-end strand-specific sequencing has become widespread. These developments clearly have significant impact(s) on transcript reconstruction and expression quantification. Chandramohan included Cufflinks (Trapnell *et al.,* 2010), HTSeq (Anders *et al.,* 2015), RSEM (Li and Dewey, 2011) and IsoEM (Nicolae *et al.,* 2010). They assessed performance by correlation; which is not as informative as precision and recall.

In 2013, the Cufflinks developers released a article with a fairly extensive benchmarking study, using simulated and real data (Trapnell *et al.,* 2013). They generated idealized data with an unpublished in-house method, TuxSeq. The data was not used to benchmark transcript form reconstruction; instead it was only used for benchmarking differential expression analysis which is not the purpose of our study. Furthermore the Cufflinks’ study only evaluated Cufflinks’ performance and did not do a comparative analysis to other methods.

Here we find that at best the methods perform just barely adequately with idealized data and relatively few splice forms, while they largely fail on more realistic data. These results point to the need for new algorithm development.

## 2 Methods

### 2.1 Simulation

We utilized the BEERS simulator (Grant *et al.,* 2011) to generate four strand-specific simulated datasets. The first dataset was generated without polymorphisms, sequencing error or intronic signal, and with all transcripts highly expressed. In this data, our goal was to assess performance in the least challenging scenario where all algorithms should in principle perform optimally. Our systematic dataset Test 1 (T1) has 13 000 (non-overlapping) genes with numbers of splice forms per gene varying from one to five. Each dataset has 50 million paired-end reads using mouse genome build mm9 (Mouse Genome Sequencing Consortium *et al.,* 2002). Dataset T1 was designed to determine upper bounds on the accuracies of the methods by providing data as ideal as possible. Three different types of alternative transcript processing were included: (i) exon skipping, (ii) alternate 3′/5′ site (start/end of transcription) and (iii) alternate splice sites at the exon/exon junctions. The vast majority of transcript forms coming from a single gene can be explained by combinations of these three types of events. The basic forms for dataset T1 were taken from real RefSeq genes (Pruitt *et al.,* 2014) as aligned to the reference genome by the UCSC genome browser (Kent *et al.,* 2002). The first 1000 genes were chosen to have one form and at least 5 exons each. For each of the three categories of alternate processing there are an additional 4000 genes with 1000 having two forms, 1000 having 3 forms, 1000 having 4 forms and 1000 having 5 forms. One transcript can have several modifications of the same type, but we did not mix different types of alternate processing events in the same gene. This way, in this dataset, we can isolate the effects of each type. In total, there are 13 000 genes comprising 43 000 splice forms. The alternate forms were generated by randomly removing some exons (exon skipping), or by randomly leaving exons off one or both ends of the transcript (truncation), or by randomly altering the start or end splice site of individual exons (mostly by multiples of three as is typically observed in real data). For these last events, we did not attempt to maintain canonical splice-signals in the exons whose lengths were modified. This could affect alignment slightly, in the case of non-annotation guided alignment, for a small number of ambiguously aligning reads. However, beyond alignment, no methods of transcript inference utilize the genome sequence, so this issue cannot affect performance at that level. In general, one should not rely too strongly on splice signals, as there are many and potentially uncharacterized ones. The reads for T1 were generated with the following parameters: read length = 100, fragment length min = 200, fragment length max = 500, fragment length median = 300, basewise error = 0%, substitution frequency = 0%, indel frequency = 0%, intron frequency = 0%. The fragment length distribution is a truncated normal with a standard deviation of 1000/3. All transcripts were expressed at the same high level with ∼40 × coverage. We take fragmentation into account yielding data that differ from strictly uniform by the randomness of the fragmentation process. The results of this idealized dataset should give effective bounds on the accuracy of the various methods.

Two additional simulated datasets were generated to assess the effect of polymorphisms and sequencing artifacts. Dataset *EP* (ENSEMBL Perfect) was generated with the same parameters as T1, except the full set of 93 778 unmodified ENSEMBL transcript models was used. Dataset *ER* (ENSEMBL Realistic) was generated with the same parameters except for the following changes, chosen to mimic real data: basewise error = 0.5%, substitution frequency = 0.1%, indel frequency = 0.05%, intron frequency = 30%. These are fairly low polymorphism rates that would be expected if comparing human sequencing data to the human reference genome. In reality the polymorphism rates in other organisms will be higher. We chose these parameters in order to attain reasonable lower bounds on algorithm accuracy in practice, where human/human comparisons are currently among the least polymorphic of all vertebrates. Intron frequency of 30% may seem high at first, but it is typical. However, since introns are so much larger than exons, the majority of intron signal results in very low coverage. Two-thirds of all transcripts are expressed, with levels of expressed transcripts given by an exponential distribution (p=.01). Short genes will be under-represented because of the fragment length distribution so we removed all genes under 200 bases.

From the IVT gene models (described below) we also generated ideal simulated data. These data were generated similar to T1, i.e. with no complicating factors, high uniform expression and perfect alignment.

The simulated datasets and ground truth files, as well as alignment files, and other supporting information, are available here: http://bioinf.itmat.upenn.edu/BEERS/bp2

### 2.2 IVT data

*In vitro* transcription (IVT) RNA was derived from an amplified plasmid library of 1062 human cDNAs (IVT), taken from the Mammalian Gene Collection (Lahens *et al*., 2014). Samples were sequenced by two ribosomal depletion protocols polyA selection and Ribo-Zero Gold kit (Epicentre catalog no. RZHM11106). Afterwards the RNA was converted into Illumina RNA-Seq libraries with the TruSeq RNA sample prep kit (Ilumina catalog no. FC-122-1001) and sequenced with an Illumina HiSeq 2000 (paired 100 bp reads). The IVT data have advantages of being a dataset where we know ground truth and it can be sequenced with standard methods, thereby capturing all normal sources of technical error. Importantly, because IVT is efficient, the expression of each base pair is theoretically the same. We used 1062 human full-length cDNAs and performed IVT-Seq. As with simulated data, the full-length transcript forms are known. In this dataset 50 genes had 2 or more splice forms. These ribosomal depletion protocols polyA selection and Ribo-Zero are the two most common protocols, which introduce within-transcript variance (Fig. 3) that cannot easily be simulated.

These data are available at GEO (accessions GSM1219408 for the polyA and GSM1219398, GSM1219399 for Ribo-Zero).

### 2.3 Alignments

Reads were mapped by the two most commonly used RNA-Seq mapping tools: TopHat (version 2.0.13 Kim *et al*. 2013), and STAR (version 2.4.0 d Dobin *et al*. 2013). Both algorithms were run with default parameters, but in two modes, both with and without annotation. In addition we generated a perfect alignment (SAM) file from ground truth. Alignment accuracy was high for both STAR and TopHat: in ER TopHat aligned 94.4% (respectively 92.63%) of the bases accurately when aligned with (respectively without) gene models. Similarly STAR aligned 94.97% (respectively 93.15%) of the bases accurately when aligned with (respectively without) gene models. In the clean datasets without indels or substitutions accuracy was ∼1.5% higher for STAR and 4% higher for TopHat. The IVT data was aligned similarly with TopHat and STAR, with each aligning ∼88–90% of the reads.

### 2.4 Genome and annotation guided reconstruction algorithms

Genome guided reconstruction algorithms were run with the recommended default parameters. We omitted Scripture and Traph because the code is unstable and we were not able to get them to run on these datasets in finite time. The Scripture approach, however, is examined in the Discussion. The genome guided algorithms that were included are AUGUSTUS (v3.0.3), Cufflinks (v2.2.1), CLASS (v2.00), FlipFlop (v1.4.1), iReckon (v1.0.8), IsoLasso (v2.6.1), MiTie (v10-27-2014) and StringTie (v1.0.0). Some of the given algorithms can be provided with gene models to improve accuracy. In reality any annotation file will have missing transcripts that are expressed, as well as non-expressed transcripts that are present. So for Test 1, we created a gene models file where we remove 40% of the transcripts that are expressed and replace them with transcript models that are not expressed. Similarly 15% EP/ER expressed transcripts were removed and replaced by unexpressed models. The algorithms were run both with and without annotation, wherever possible. For the ENSEMBL datasets EP and ER the results are broken down further by depth of coverage: low (1 × –10×), medium (10 × –100X) and high (100 × –∞×).

### 2.5 *De* *novo* reconstruction algorithms

The following *de novo* reconstruction algorithms were run on the simulated datasets: Trinity (v2014 07 17), SOAPdenovo-Trans (v1.03) and TransABySS (v1.5.2). All of the tools were run with default settings. In order to compare the *de novo* reconstruction algorithms to the genome guided tools, the inferred transcripts were aligned back to the reference genomes with GMAP (version 2014-10-22). All transcripts that were assigned successfully were used to evaluate the algorithms performance. We did not benchmark Oases because it is unstable and we were not able to run it on our datasets.

### 2.6 Accuracy metrics and performance evaluation

We compared the inferred transcript models produced by each algorithm with the true models to calculate accuracy metrics. If an inferred transcript model has the right number of exons and agrees with a known model at all sites of exon/exon junctions, then we call it a true positive. As the start and end of transcription are notoriously difficult to predict, we do not require them to be inferred correctly, just the internal junctions need be correct to be a true-positive. If at least one of the exon/exon junctions is wrong, we call it a false positive. If a model is expressed but not reported by the algorithm, we call it a false negative. This could be problematic if combining high and low expressed splice forms into one analysis. In T1 all genes are expressed at a high level so this is not an issue—and any expressed transcript that was missed can reasonably be called a false-negative. In contrast, EP and ER were subdivided into three categories: low, medium and high. We do not expect a very low false negative rate on the low expressers, but we should expect it from the other categories.

When predicting transcript structure as a sequence of exon start- and end-coordinates, there are an astronomical number of possible true negatives—any sequence of an even number of consecutive coordinates is a putative transcript. Therefore, as long as the algorithm itself does not return an astronomical number of putative transcripts, the false positive rate will be very close to zero and the specificity will be very close to one. This remains true even if every predicted transcript is a false positive. Instead we take an approach that has been established in the literature which is more informative (see, e.g. Hu *et al.,* 2014). The approach is to report the recall (the number of correctly constructed forms divided by the total number of real forms) and precision (true positives divided by the sum of true positives and false positives). We identify the regions where the recall is <25% or the precision is <66.6% as being a region where the error rates are questionable for use in practice (shaded region in our graphs). In T1 we split the results by the category of processing event, the number of forms per gene, and the depth of coverage where appropriate. Finally, we compared the assessed FPKM values to the true FPKM by Pearson correlation (after filtering extreme outliers). The Ruby scripts used to do the analysis are available here: https://github.com/khayer/benchmarking_scripts.

## 3 Results

Most algorithms which rely on a genome alignment can be run in two possible modes, depending on whether or not they utilize transcript model annotation. In the first mode the algorithm determines which of the annotated forms are expressed and then further uses the annotation as a guide to determine novel forms. The second mode attempts to determine the forms of the expressed transcripts from scratch, with no transcript annotation provided. The latter is a considerably harder problem.

Annotation is never perfect. To model this, for dataset T1 we hid roughly 40% of the expressed transcripts, at random, and replaced each one with a different unexpressed transcript of the same gene. Unexpressed transcripts that are called as expressed constitute the false positives. For EP and ER also half of the expressed transcripts were hidden, however those datasets follow a realistic spectrum of expression so that approximately one third of the transcripts are not expressed. Therefore, it is not necessary to introduce further unexpressed transcripts as was done for T1. Supplementary Tables S1–S3 give a summary of all of the available figures.

**Fig. 4.**
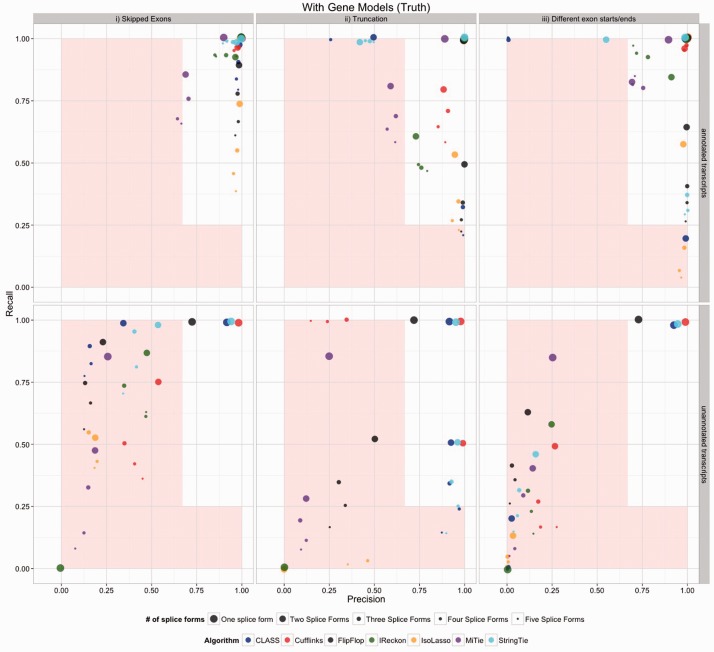
Accuracy results for simulated dataset T1 for the methods which utilize a reference genome. This represents the most ideal case where all genes are highly expressed, there are no polymorphisms and there are no alignment errors. Splicing is divided into three types, the only cases where precision was above 90% in the first two types are when there is a single splice form. The analysis was run with gene annotation provided

Although some algorithms perform well with perfect data and a single splice form, they tend to have difficulty predicting multiple splice forms. Figure 4 represents the most ideal case. The top row represents the correctly annotated transcripts. In this case a false positive is an annotated transcript that is not expressed but is called expressed and a false-negative is an annotated and expressed transcript that is not called expressed. No algorithm should be expected to do this well in practice, but it gives a bound on the accuracy. Even in this case, several algorithms are out of the comfort zone in some if not most cases. Having differing exon start/ends (splicing category iii) gave the algorithms the most difficulty across the board; note that the methods were given the perfect alignment, so the category iii issues are not due to alignment artifacts. In the lower panel we see the accuracy for incorrectly annotated transcripts. Here a false-positive is an inferred (un-annotated) transcript that is not expressed and a false-negative is any of the 15% of hidden transcripts that are expressed that were not called as expressed. Most methods perform well on the genes with one transcript, but the accuracy rates for unannotated transcripts are low across the board. When the data are aligned with TopHat or STAR the error rates naturally increase (Supplementary Fig. S2–S5, S15–S16). As expected, the *de novo* methods performed worse than the genome-alignment guided methods (Supplementary Fig. S6). We conclude that although use without annotation is a common and intended application, the error rates of all algorithms on real data are high.

**Fig. 5.**
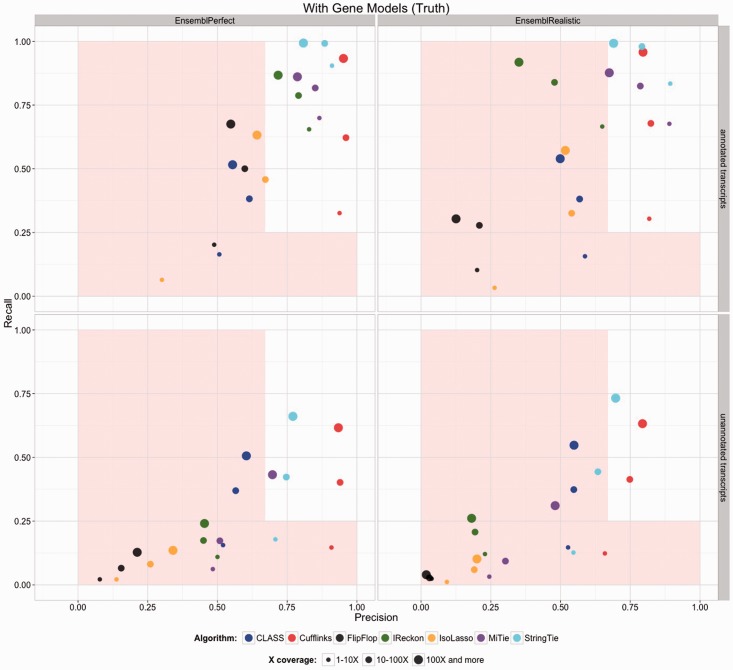
Accuracy results for simulated datasets EP and ER. This represents the most ideal case where all genes are highly expressed, there are no polymorphisms and there are no alignment errors. Results are given separately for low, medium and high depth of coverage. Analyses were run with gene models provided

EP and ER represent more realistic datasets because they contain the full complexity of the ENSEMBL annotation and they span a range of expression levels similar to real data. Additionally ER contains polymorphisms (in the form of substitutions and indels), sequence error and intron signal. Because in practice algorithms will not have access to perfect annotation, the gene models provided were modified models whereby 15% of the expressed transcripts were hidden. Figure 5 shows the results when the algorithms are provided with perfect alignments. The top row represents properly annotated transcripts and the bottom row represents hidden transcripts. The statistics are stratified by depth of coverage. In this case, on correctly annotated transcripts, Cufflinks and StringTie stay in the comfort zone in most cases, with Cufflinks tending to have better precision and StringTie better recall. It is notable that often precision goes down with depth-of-coverage. The reason for this is that with more reads, most algorithms find more ways to go wrong. Without annotation very few data points of any category are in the comfort zone (Supplementary Fig. S7). Only Cufflinks and StringTie appear to be potentially viable. There will of course also be other un-modeled biases and factors in real data, such as position specific biases. So these bounds on the accuracy are certainly quite conservative. The results when TopHat and STAR are used instead of a perfect alignment are given in the Supplementary Figures S8–S11. Again the *de novo* methods underperform the alignment based methods (Supplementary Fig. S12).

**Fig. 6.**
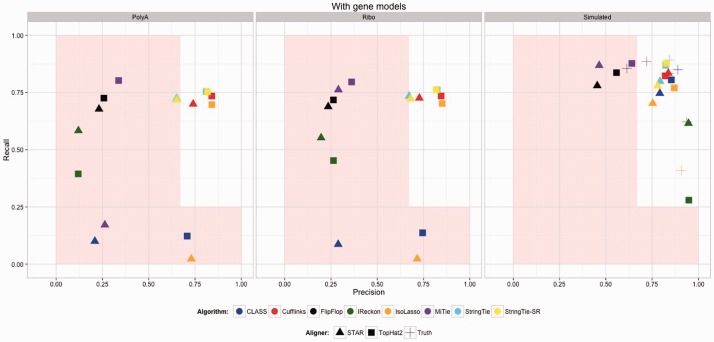
Accuracy results for IVT data. Analyses were run with gene models provided. Two ribosomal depletion protocols are represented. The rightmost panel shows the results on simulated data, for comparison

We turn next to the IVT data to obtain information on the impact of the other effects of real sequencing that we could not model. In this case, since this is real sequence data, we could not provide an error-free alignment. The annotation given was of the known structure of the 1062 transcripts. For the sake of comparison, from these gene models we also generated ideal simulated data based on these gene models (Fig. 6 rightmost panel). Apparently polyA selection caused more problems across the board as compared with Ribo-Zero. Unfortunately, however, the vast majority of RNA-Seq is being generated with the polyA selection protocol. These results are an order of magnitude worse than for the ER data, which speaks to the complications introduced by un-modeled factors. For the genes with more than one splice form, the results were worse still. Note that we get a different separation of methods in the simulated data (right panel) compared with the real data (left and center panels). This indicates comparisons based on simulated data do not necessarily indicate which methods to prefer.

**Fig. 7.**
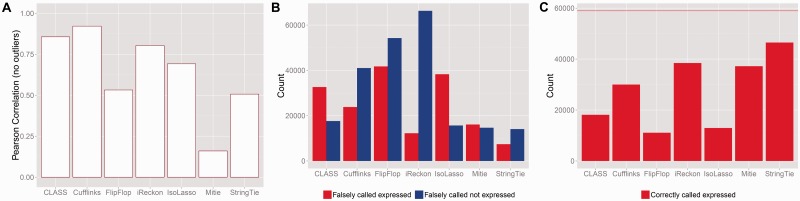
**(A)** Correlation of true FPKM with the inferred on dataset ER. Only transcripts where both the true and inferred values are positive were included. Extreme outliers were also removed. The set sizes for each correlation are given in (**C**) (**B)** Bars on the left show the number of transcripts where the true expression is zero but the algorithm assigned it positive expression, bars on the right show the number of transcripts where the true expression is positive but the algorithm assigned it zero expression. (**C)** This shows the number of transcripts where the true expression is positive and the algorithm gave it positive expression. The horizontal line indicates the total number of truly expressed transcripts

To assess the quality of the inferred quantified FPKM values, we computed the Pearson correlation between the true FPKM and the inferred values. The true FPKM was determined not from the theoretical intensity of the transcripts, but from the actual true number of reads that came from each transcript—exonic reads only. We filtered out extreme outliers. We also removed all cases where the true expression is zero but the algorithm gave it positive expression, or where the true expression is positive and the algorithm gave it zero. We call these *on/off* errors. If we do not do this, then all correlations are very low and uninformative. We separated out how often the on/off errors occur and graphed them separately (Fig. 7B). We did not report outlier statistics, since there was only a handful. But some were extreme, e.g. Cufflinks gives an FPKM value in the 7000 ranges for one gene that is not expressed at all.

## 4 Discussion

These results provide objective and conservative bounds on the error rates of transcript inference and quantification algorithms and found that none of them can be considered highly accurate. One has to decide if chasing differential splicing is worth the potential disadvantages of dealing with many false positives in the downstream analysis. Our experience is that most groups are after evident effects, meaning moderately high fold change of moderately to highly expressed and well annotated genes. However, many such groups follow the path of transcript level analysis, because it has been presented as the standard thing to do. Some groups certainly must worry about alternate splicing, however most groups would find what they are looking for by performing a much more straight forward gene level analysis. And a significant portion of those who need more than a gene level analysis would find exon/intron/junction level analysis sufficient. Therefore, employing the current state of transcript level analysis should only be done with considerable forethought.

Transcript structure determination, either *de novo,* or with annotation, is a challenging problem. To assess how the most recent versions of algorithms perform, we devised a set of tests using simulated and *in vitro* transcribed RNA-Seq data. We generated one clean dataset (T1) simulated from 13 000 genes with one to five splice forms per gene. T1 used paired end, 100 base per end, sequencing. As this is ideal data, it has a perfect representation of all bases, no sequencing or mapping errors and no polymorphisms. We also simulated a realistic dataset (ER) from ∼97 000 ENSEMBL transcripts. We generated this dataset with polymorphisms, error rates and intron signal. Transcripts follow a distribution of intensities at rates consistent with what is typically observed in quality data in practice. Finally, we used *in vitro* transcription data for 1062 full length human cDNAs, where mapping errors, uneven base representation, and polymorphisms are unavoidable. With all of these datasets, we know the truth, making them informative for evaluating the performance of transcript assembly algorithms.

On *perfect* data (T1) with a *single* splice form (as in Fig. 2A), most algorithms perform reasonably well, with fairly high precision. All algorithms seem to be optimized to detect exon skipping events, while of the three types of splicing investigated, variable length exons present the greatest challenge. Detecting truncated genes on the other hand has high precision across the board but the recall of many algorithms suffered. With clean data and just two forms per gene, the error rates for all algorithms go up considerably. If one must do transcript level analysis then Cufflinks and StringTie are among the best performers. In summary, all algorithms designed to delineate transcript forms tend to make many false discoveries, even on perfect data.

The IVT data (IVT) are perhaps the most informative control dataset, because rather than modeling errors, alignment, polymorphisms, base representation, it simply has them as they occur naturally—yet we still know the ground truth in terms of what the true transcript models are. Error rates with this data are considerably higher, even though 95% of genes had only a single form. Put simply, on this real benchmark data, with 95% of transcripts having only a single form, all algorithms predict at least 20% as many false transcripts as true ones, and at best roughly 25% or more of the true ones were missed. In the IVT data, we also had a limited subset of ∼50 genes with more than one transcript. On this limited set of real data, performance was yet worse.

It is instructive to look at what aspects of the popular algorithms need improvement in order to increase their accuracy. Cufflinks uses junction and coverage information to determine local alternative splicing events. However, it often assembles the local information into global transcripts in all possible ways that are consistent with the short reads, and this is the source of many of the false positives. Because the integrity of the transcripts is lost in this approach it is not clear what advantage it could have over a more local analysis, such as at the exon/intron/junction level. Another problem is that it does not effectively sort out exon signal from intron signal. In most RNA-Seq datasets there is some amount of signal at almost every base of almost every intron. This intron signal is often assembled into long exons, resulting in more false positives, as seen in the ER dataset. Perhaps reading-frame information could be used to limit this type of artifact. There is also generally a smattering of junctions that connect exons to the middle of introns (Fig. 2B) yet these probably do not represent real exon/exon junctions. The challenge is for algorithms to find a way to ignore the intron and spurious junction signal to get at the clean transcript models.

Scripture uses an even more liberal approach by essentially ignoring junction information altogether and instead taking a ‘peak finding’ approach to identify putative exons and then connecting them in all possible ways. This approach also suffers from the fact that there is insufficient information to connect local inferences into global inferences and so it constructs a large number of false positives by joining local effects to make full-length transcripts. We have also observed that Scripture tends to find many peaks in introns where the signal was generated via a uniform model, and so should not have any significant peaks. The extreme overcalling of forms makes it unclear how to utilize the output of Scripture in a practical way.

The *de novo* methods such as Trinity are trying to solve a much harder problem by not using the information coming from the genome sequence. If no genome exists then this approach might provide information that can be informative. However, for model organisms, and many others for which a reference genome is available, and which have some degree of quality community annotation, it is hard to see any benefit of using a *de novo* approach.

We have seen in Figure 2B regions that present a particular challenge to transcript level analysis. Indeed it is not even clear that these are true splicing events and not just artifacts of sequencing or alignment. So the first task in using RNA-Seq to perform transcript level analysis should be to determine what part of these challenging regions represents true biology that is worth quantifying and what part of it is artifactual.

In a survey of 315 random papers from PubMed that perform RNA-Seq studies and for which some method of feature quantification was employed, we found transcript level inference is quite popular, being done more than half the time. In particular Cufflinks is cited 123 times and Trinity is cited 24 times. A couple methods are so new that we do not expect citations yet, such as StringTie. Many other papers do not perform transcript *inference* but still attempt to do transcript level analysis via methods which simply assign quantifications to a fixed set of annotated transcripts. In particular, out of the 315 publications surveyed, HTSEQ is cited 45 times, RSEM 19 times, simple counting 17 times and CLCBio Genomics Workbench 10 times. Only a small minority of groups work with exon or gene level analysis; however, our results indicate that such an analysis may often be much more practical and efficient.

## 5 Conclusion

Investigators using RNA-Seq want to know which transcripts are present and their expression levels. Full-length transcript reconstruction from short read data has emerged as a potential solution to the problem. However, short reads are noisy and fundamentally lack the information necessary to build globally accurate transcripts. Despite this, several algorithms have gained widespread usage, underscoring the importance of more research into this problem. Most likely, a satisfactory solution will involve an evolution not just in the algorithms, but in the nature of the data. It is likely this problem will not improve to the point of being practical until much longer reads are available and until the ribosomal depletion protocols improve. Finally, it remains possible that some keen insight into how to identify and effectively utilize signals in the genome could emerge that helps to solve this problem. Regardless, benchmarking studies such as those presented here will remain a critical component to realizing these important goals.

## Supplementary Material

Supplementary Data
