# Supplementary material for: Benchmark analysis of algorithms for determining and quantifying full-length mRNA splice forms from RNA-seq data
Source: Bioinformatics. 2015 Sep 3;31(24):3938–45. doi: 10.1093/bioinformatics/btv488 (PMC4673975; doi:10.1093/bioinformatics/btv488)
Supplement: Supplementary Data [file supp_31_24_3938__index.html]

Benchmark analysis of algorithms for determining and quantifying full-length mRNA splice forms from RNA-seq data — Benchmark analysis of algorithms for determining and quantifying full-length mRNA splice forms from RNA-seq data — Supplementary Data 

# Benchmark analysis of algorithms for determining and quantifying full-length mRNA splice forms from RNA-seq data

## Supplementary Data

files

- Supplementary Data - zip file
