# Supplementary figures and images for: Benchmark analysis of algorithms for determining and quantifying full-length mRNA splice forms from RNA-seq data
Source: Bioinformatics. 2015 Sep 3;31(24):3938–45. doi: 10.1093/bioinformatics/btv488 (PMC4673975; doi:10.1093/bioinformatics/btv488)

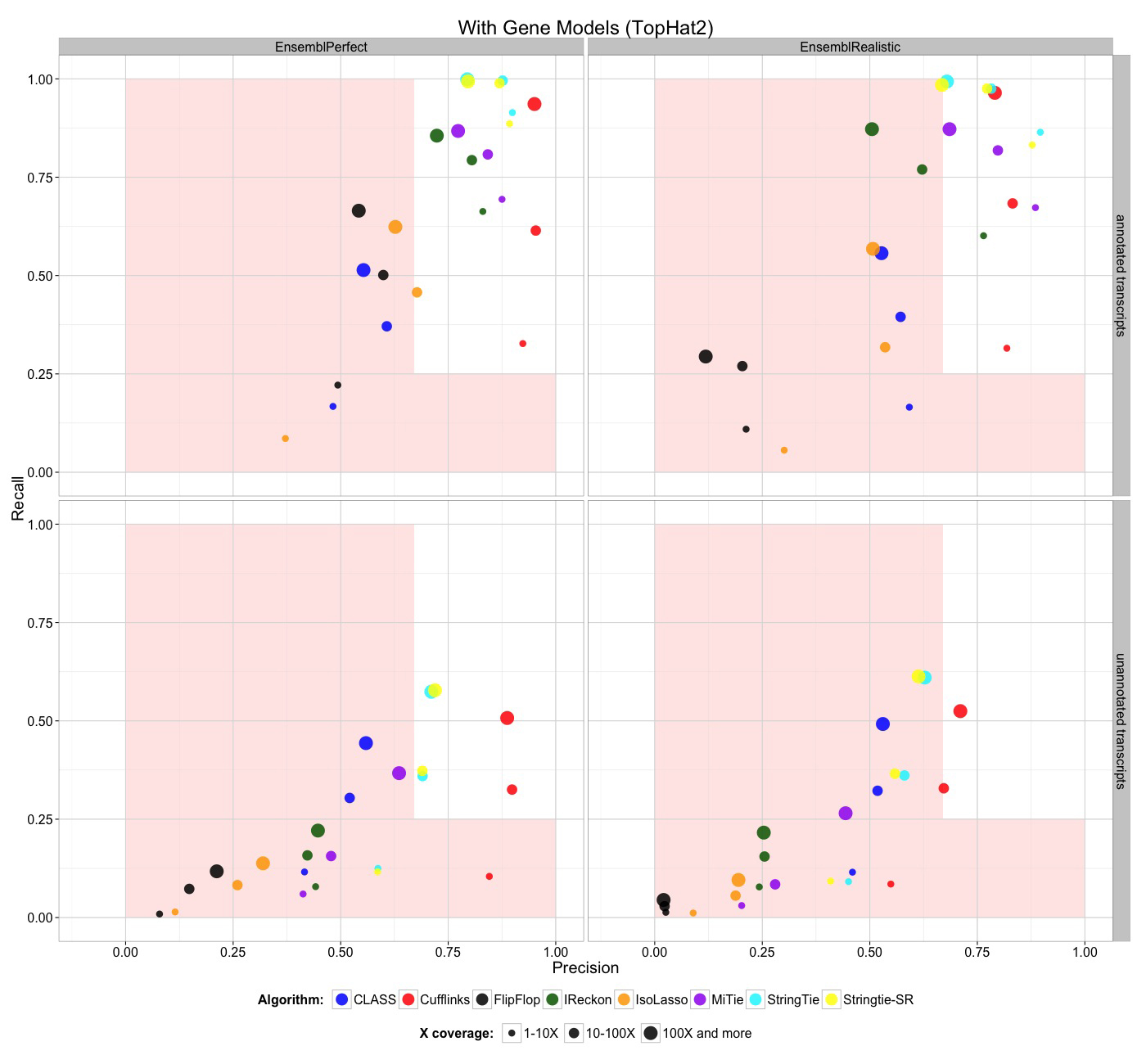

Supplement: Supplementary Data [file supp_btv488_suppl_data.zip › figS18.jpeg]

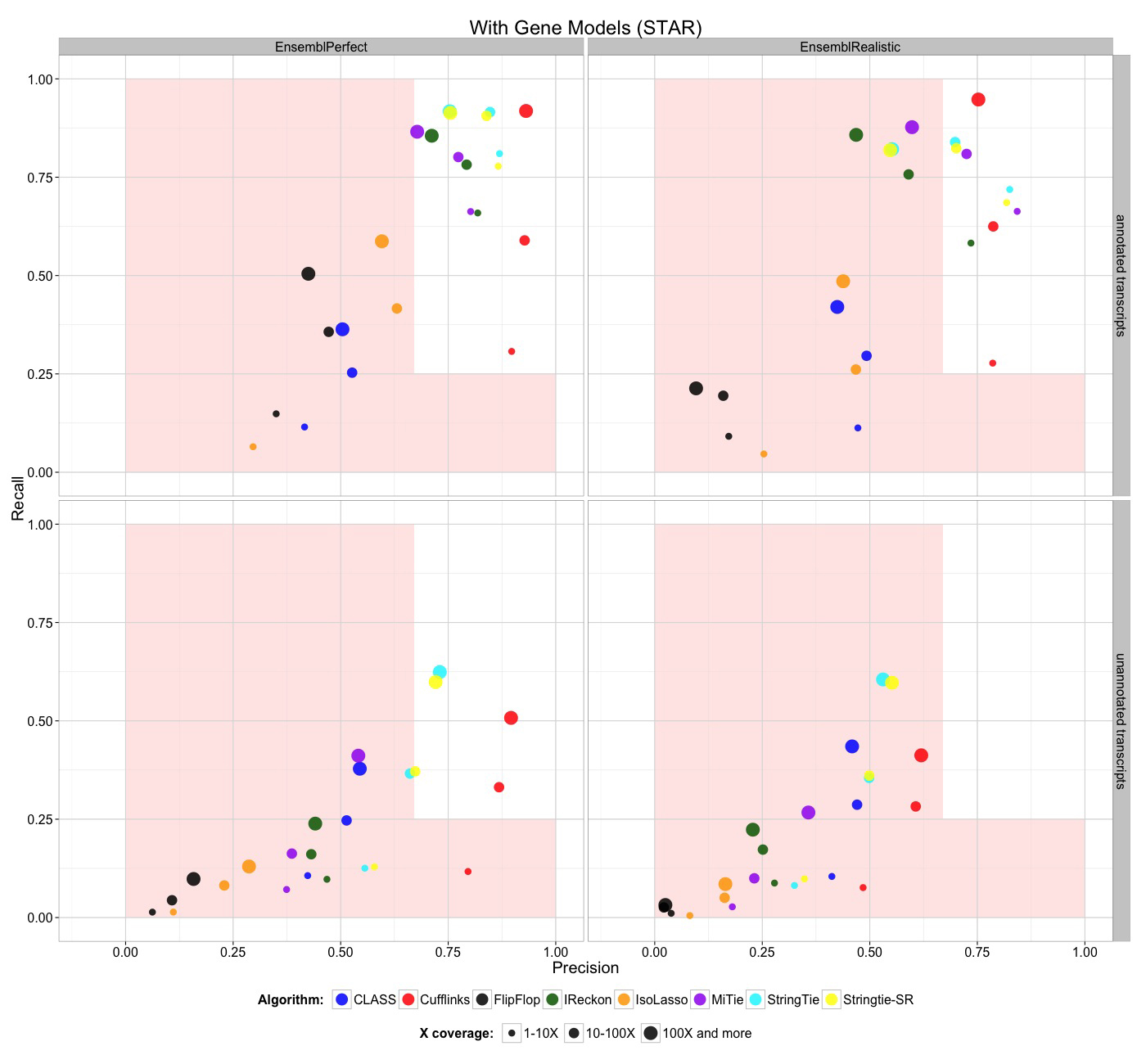

Supplement: Supplementary Data [file supp_btv488_suppl_data.zip › figS19.jpeg]

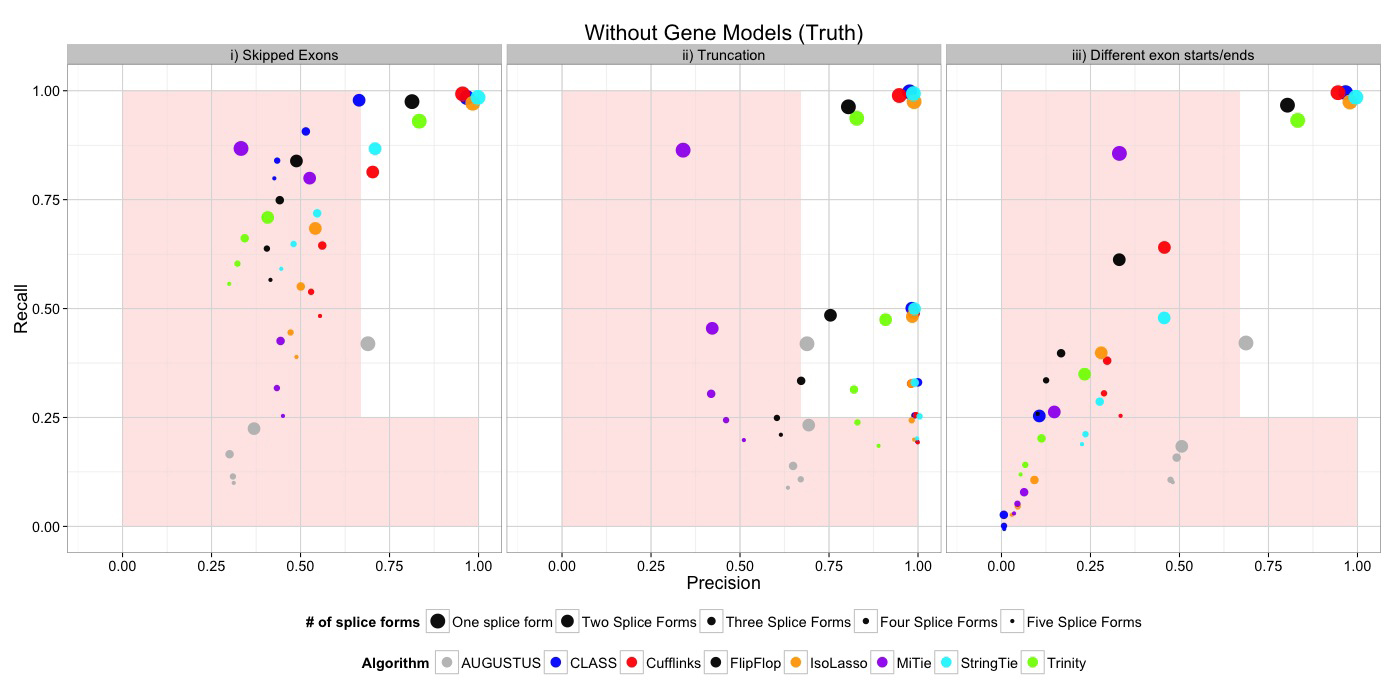

Supplement: Supplementary Data [file supp_btv488_suppl_data.zip › figS1.jpeg]

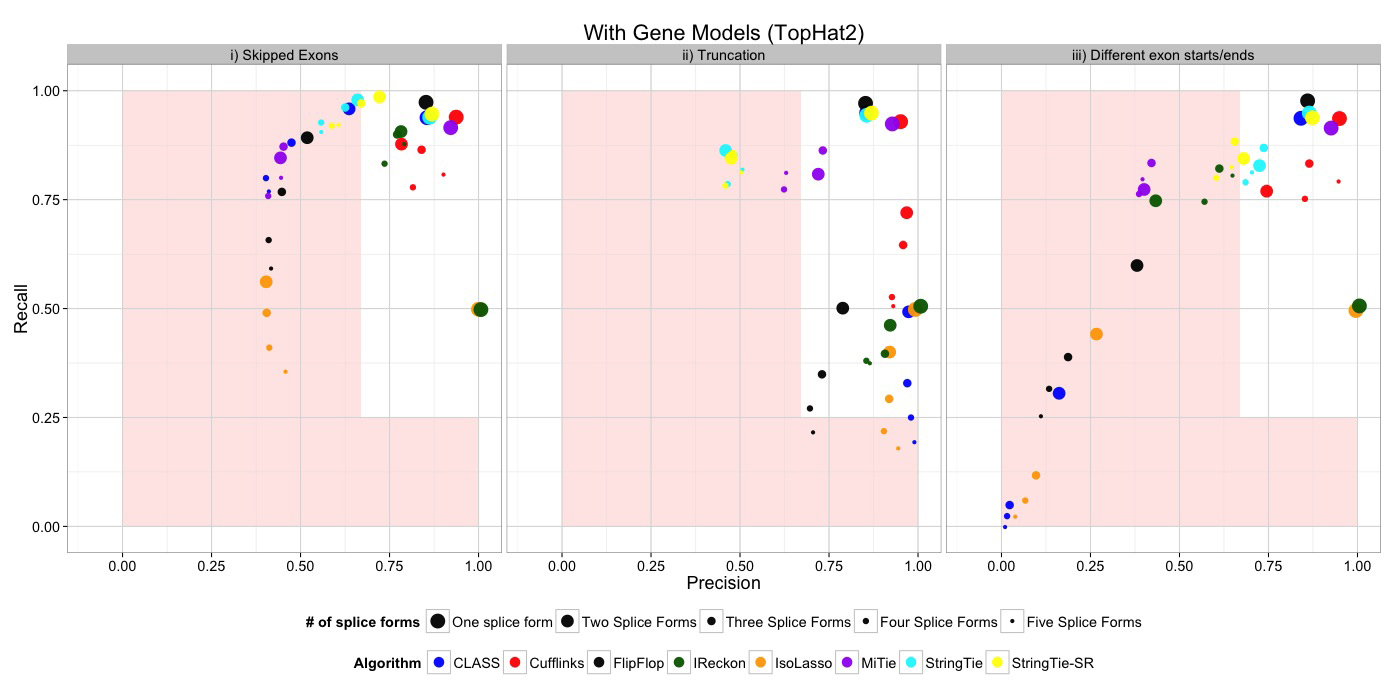

Supplement: Supplementary Data [file supp_btv488_suppl_data.zip › figS2.jpeg]

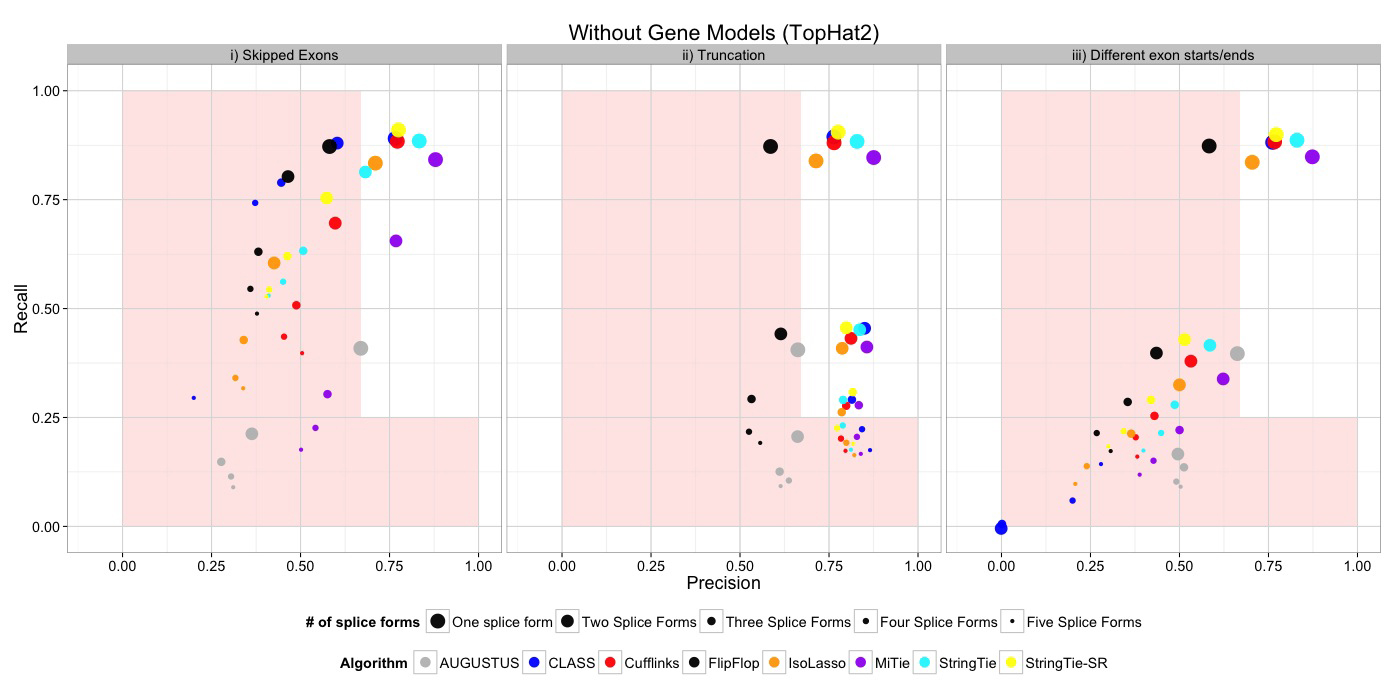

Supplement: Supplementary Data [file supp_btv488_suppl_data.zip › figS3.jpeg]

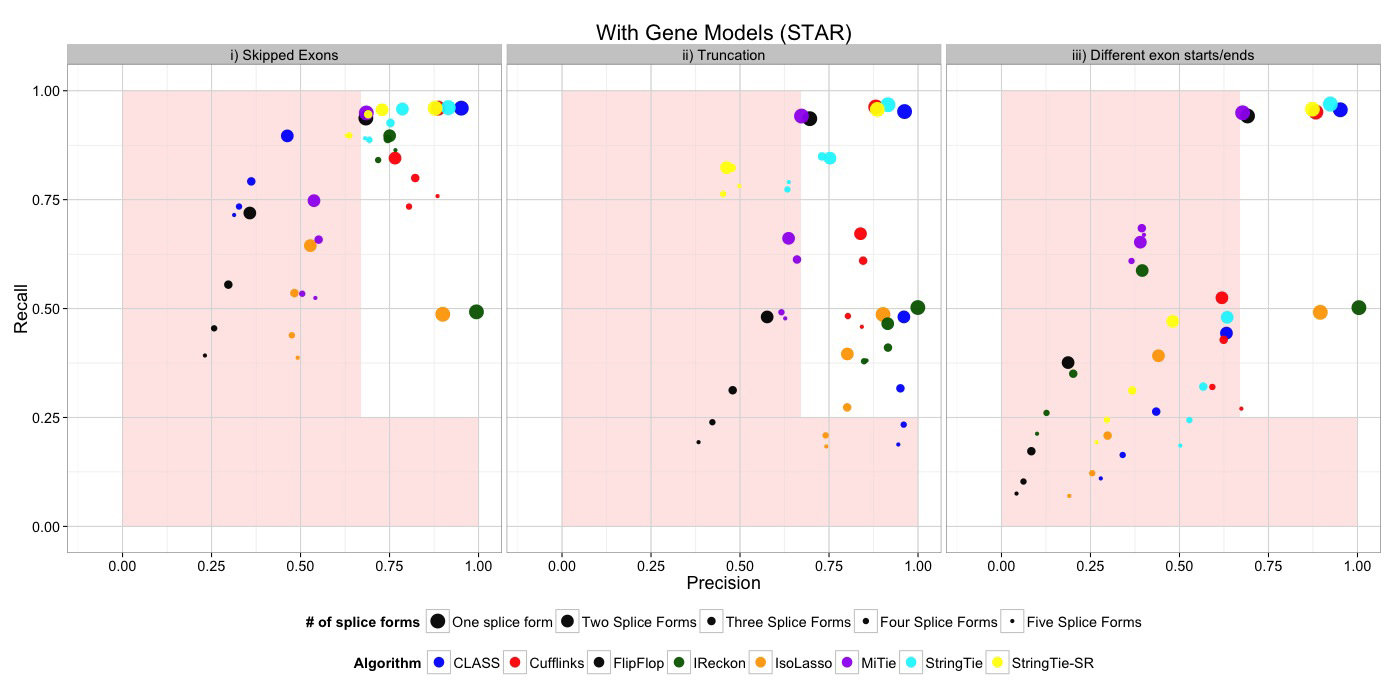

Supplement: Supplementary Data [file supp_btv488_suppl_data.zip › figS4.jpeg]

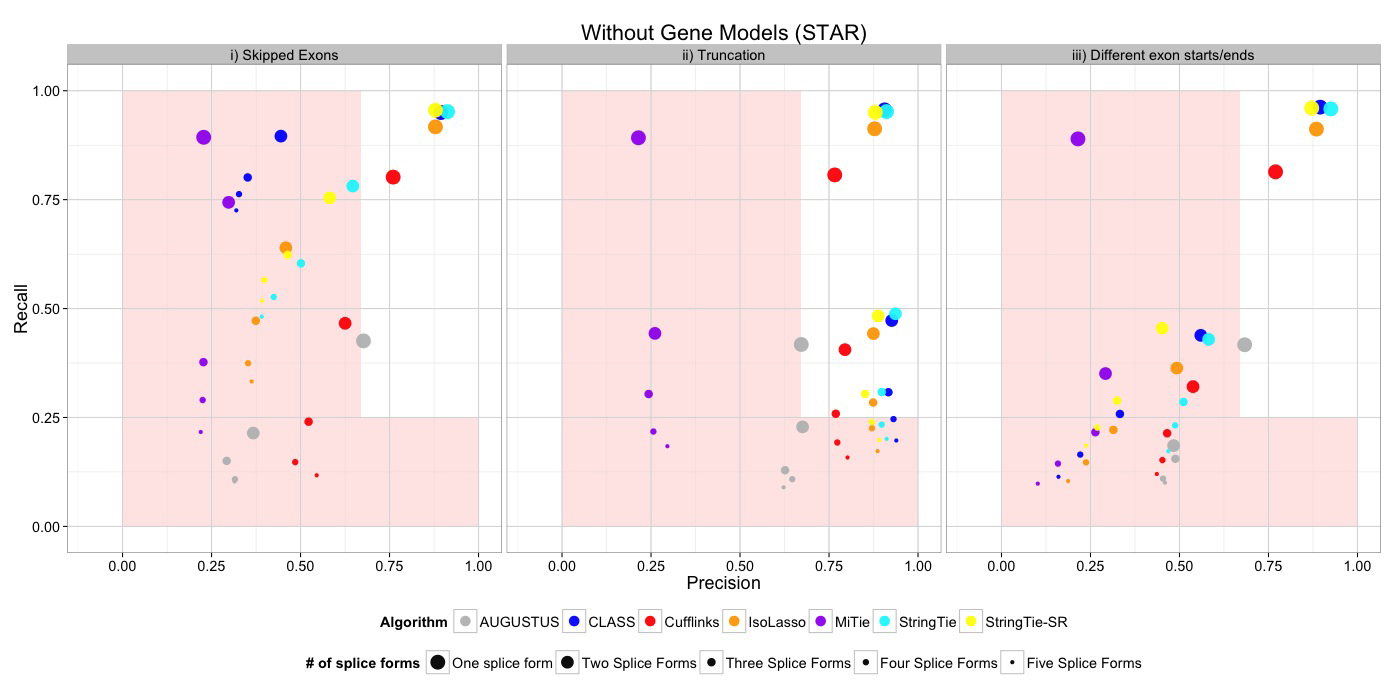

Supplement: Supplementary Data [file supp_btv488_suppl_data.zip › figS5.jpeg]

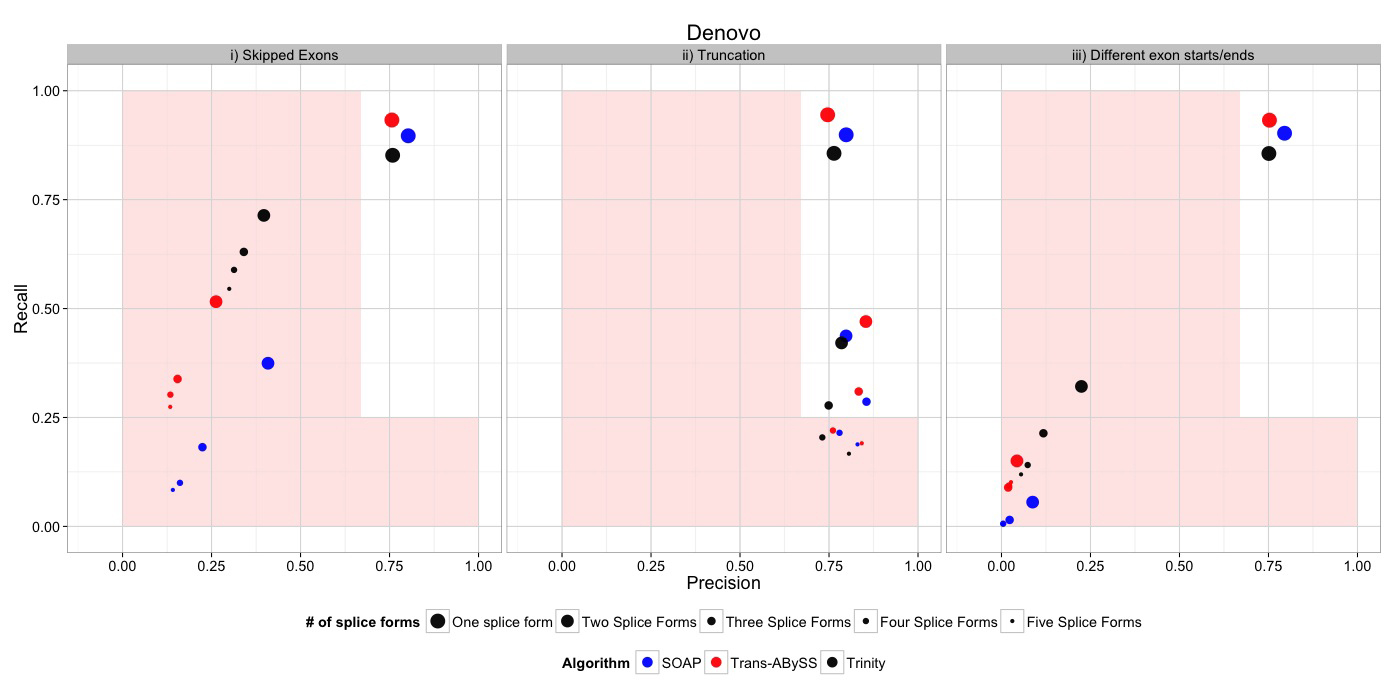

Supplement: Supplementary Data [file supp_btv488_suppl_data.zip › figS6.jpeg]

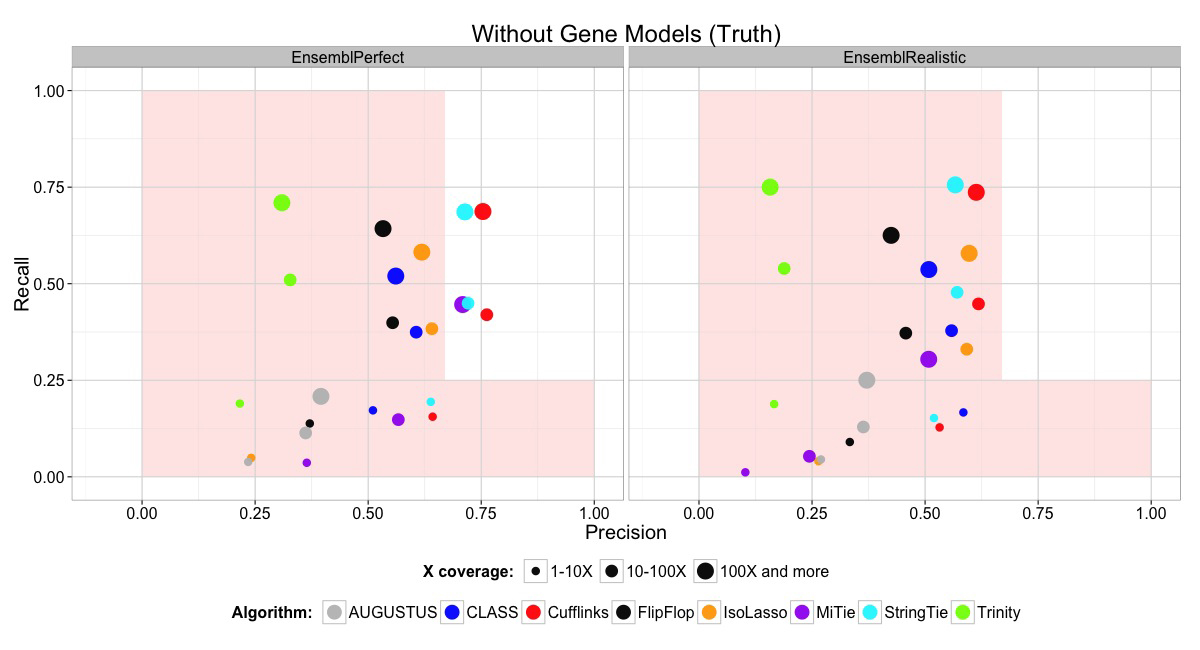

Supplement: Supplementary Data [file supp_btv488_suppl_data.zip › figS7.jpeg]

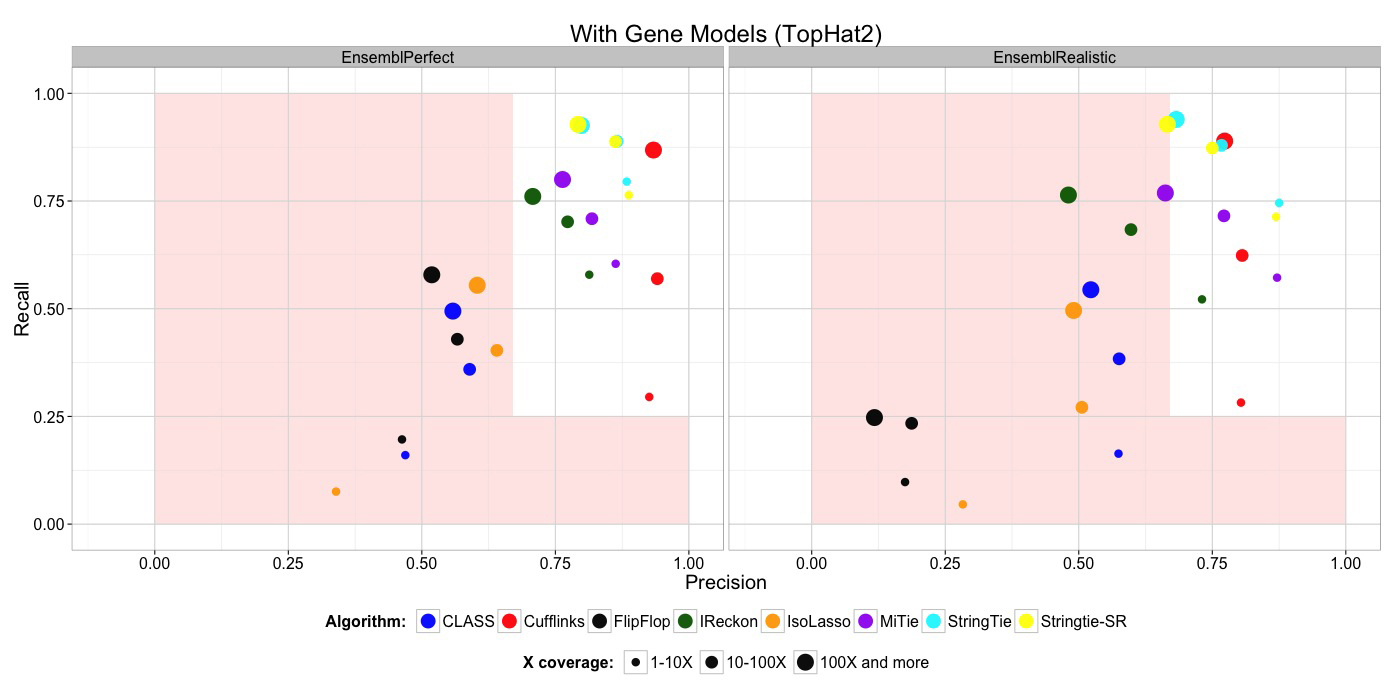

Supplement: Supplementary Data [file supp_btv488_suppl_data.zip › figS8.jpeg]

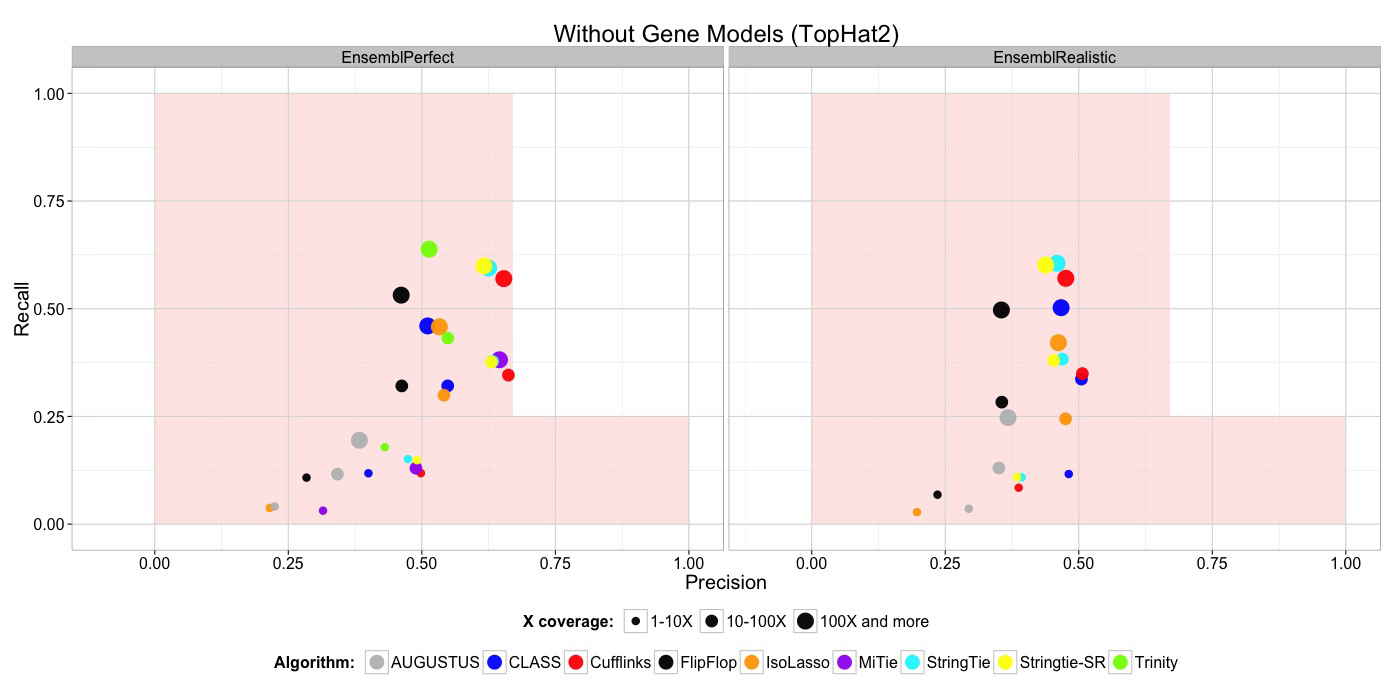

Supplement: Supplementary Data [file supp_btv488_suppl_data.zip › figS9.jpeg]

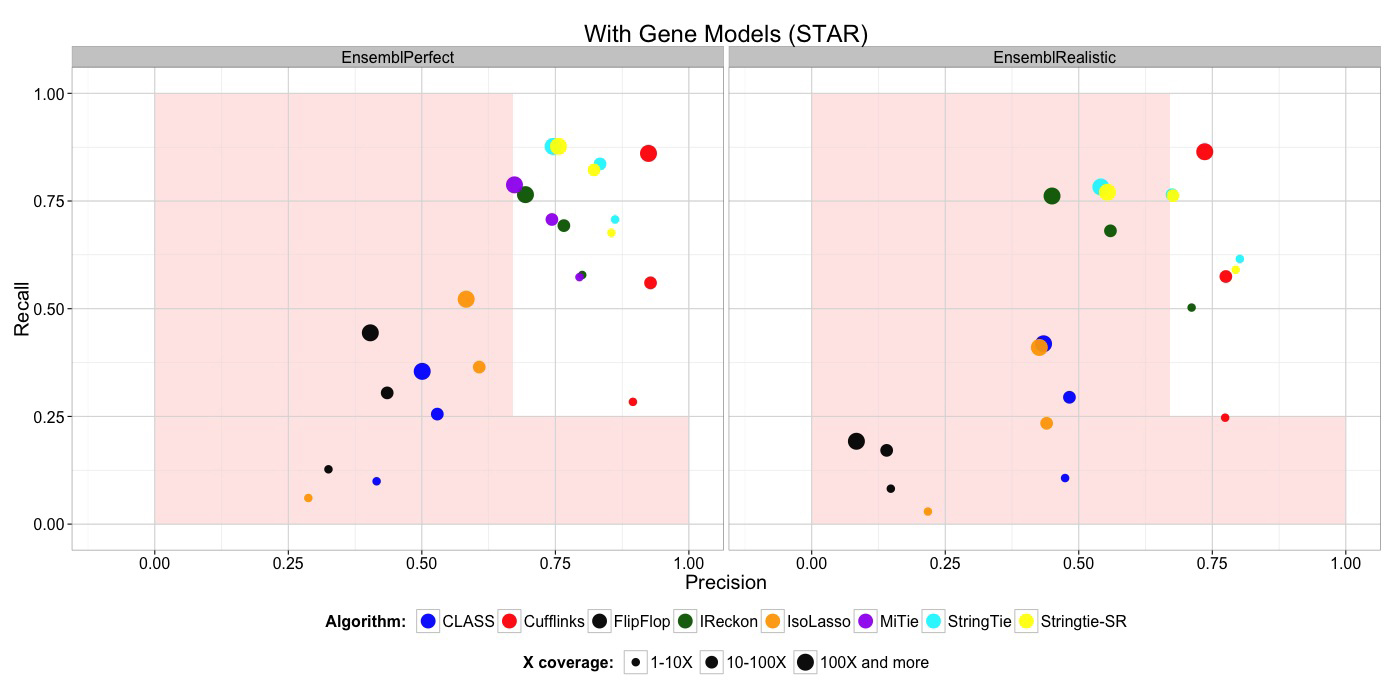

Supplement: Supplementary Data [file supp_btv488_suppl_data.zip › figS10.jpeg]

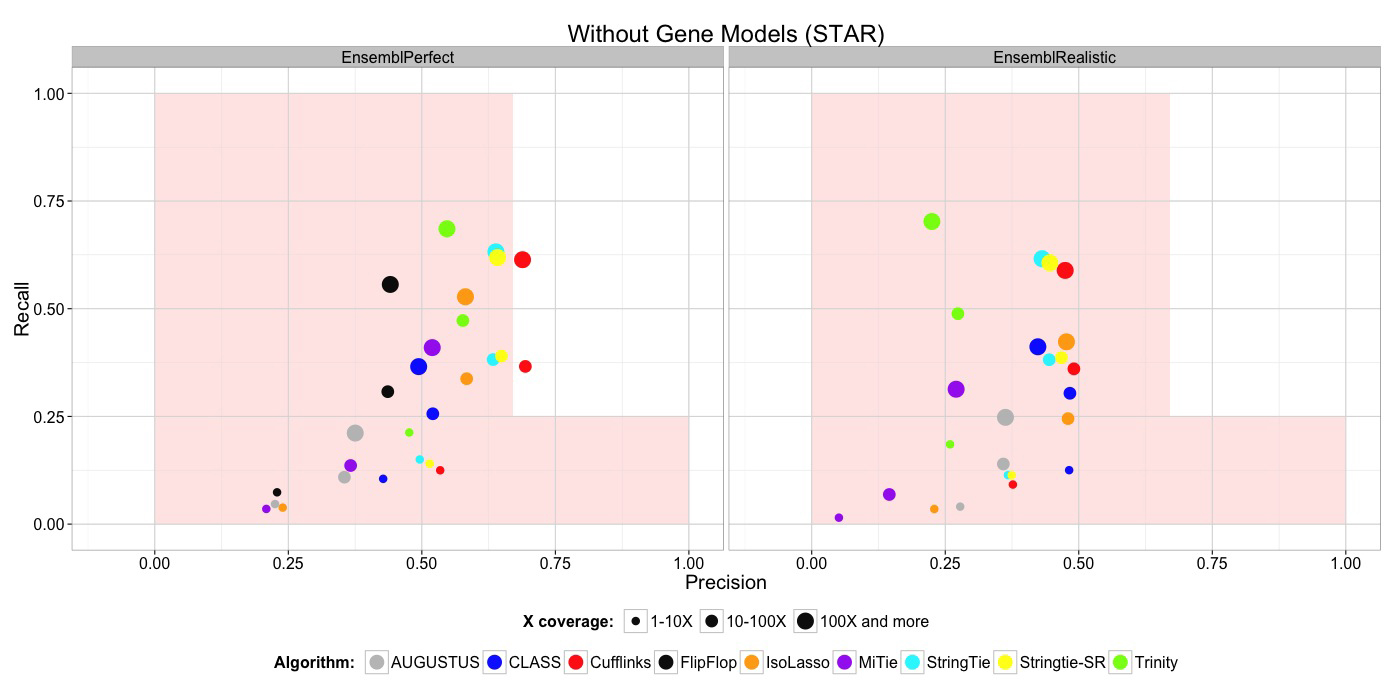

Supplement: Supplementary Data [file supp_btv488_suppl_data.zip › figS11.jpeg]

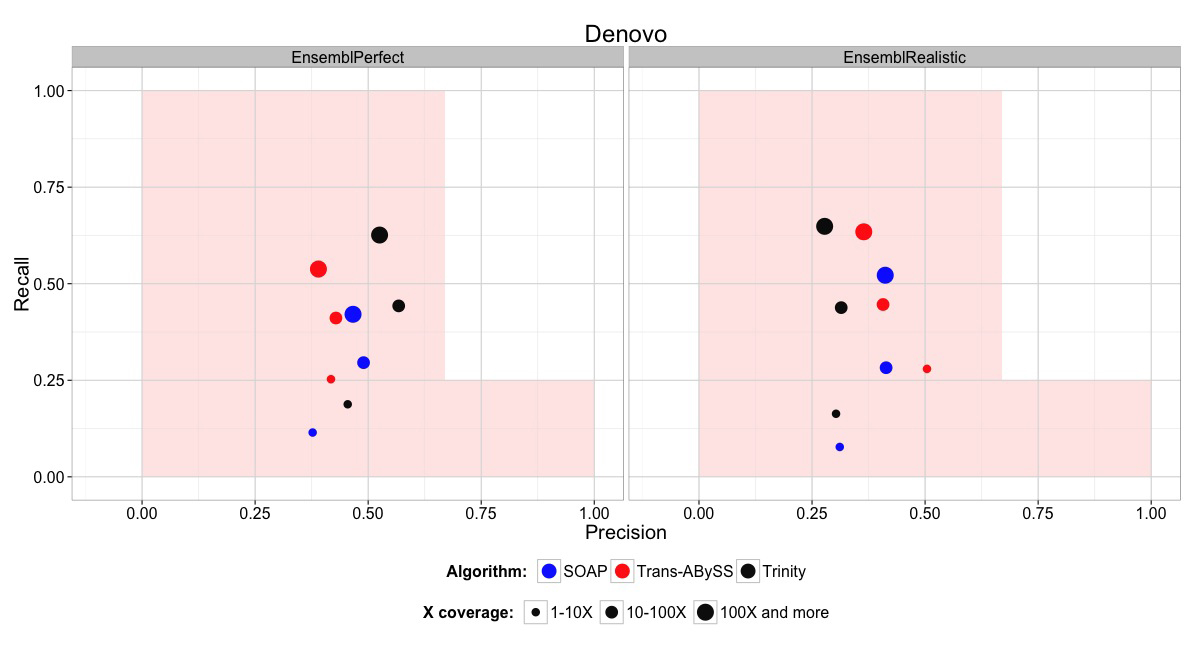

Supplement: Supplementary Data [file supp_btv488_suppl_data.zip › figS12.jpeg]

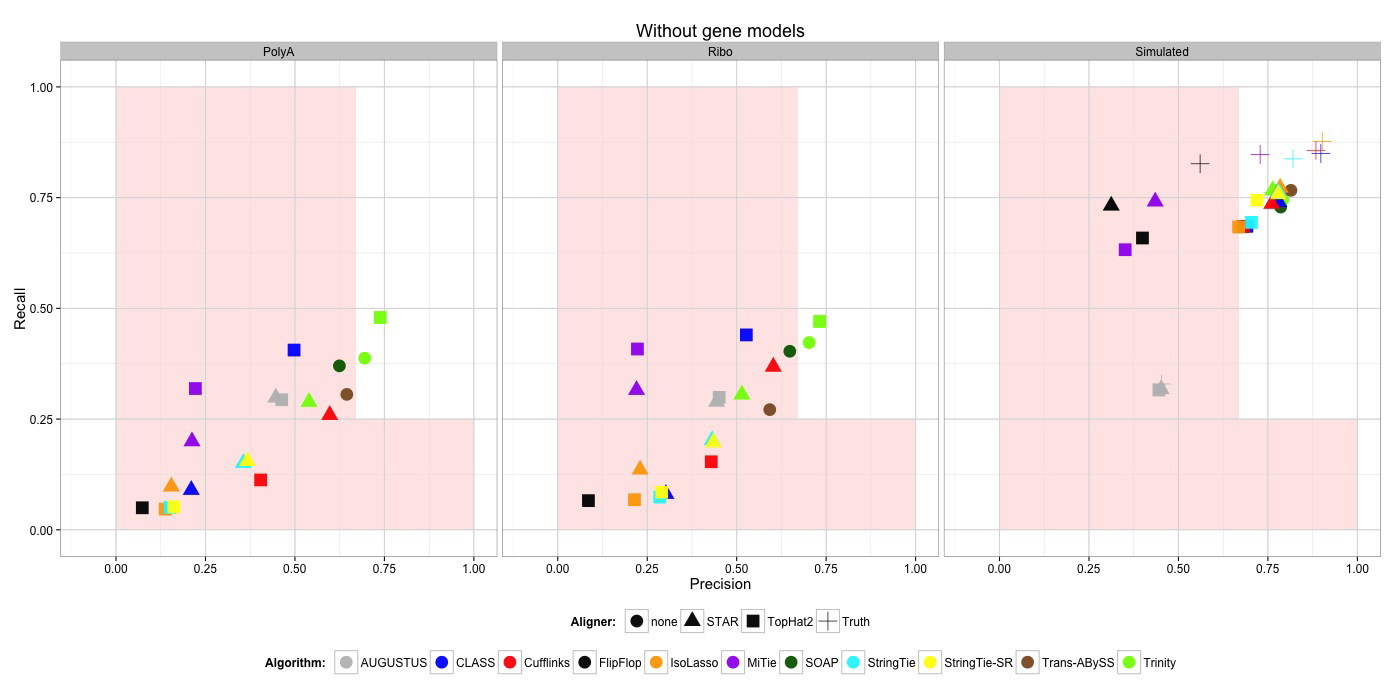

Supplement: Supplementary Data [file supp_btv488_suppl_data.zip › figS13.jpeg]

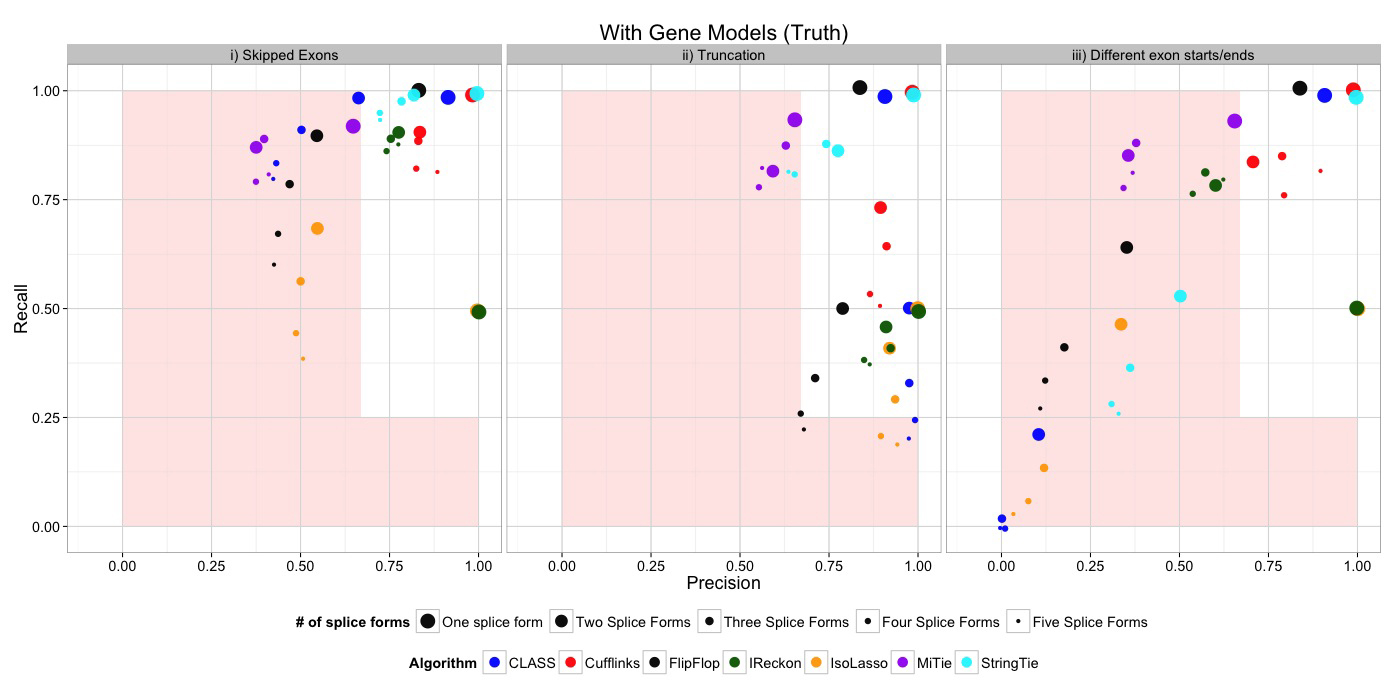

Supplement: Supplementary Data [file supp_btv488_suppl_data.zip › figS14.jpeg]

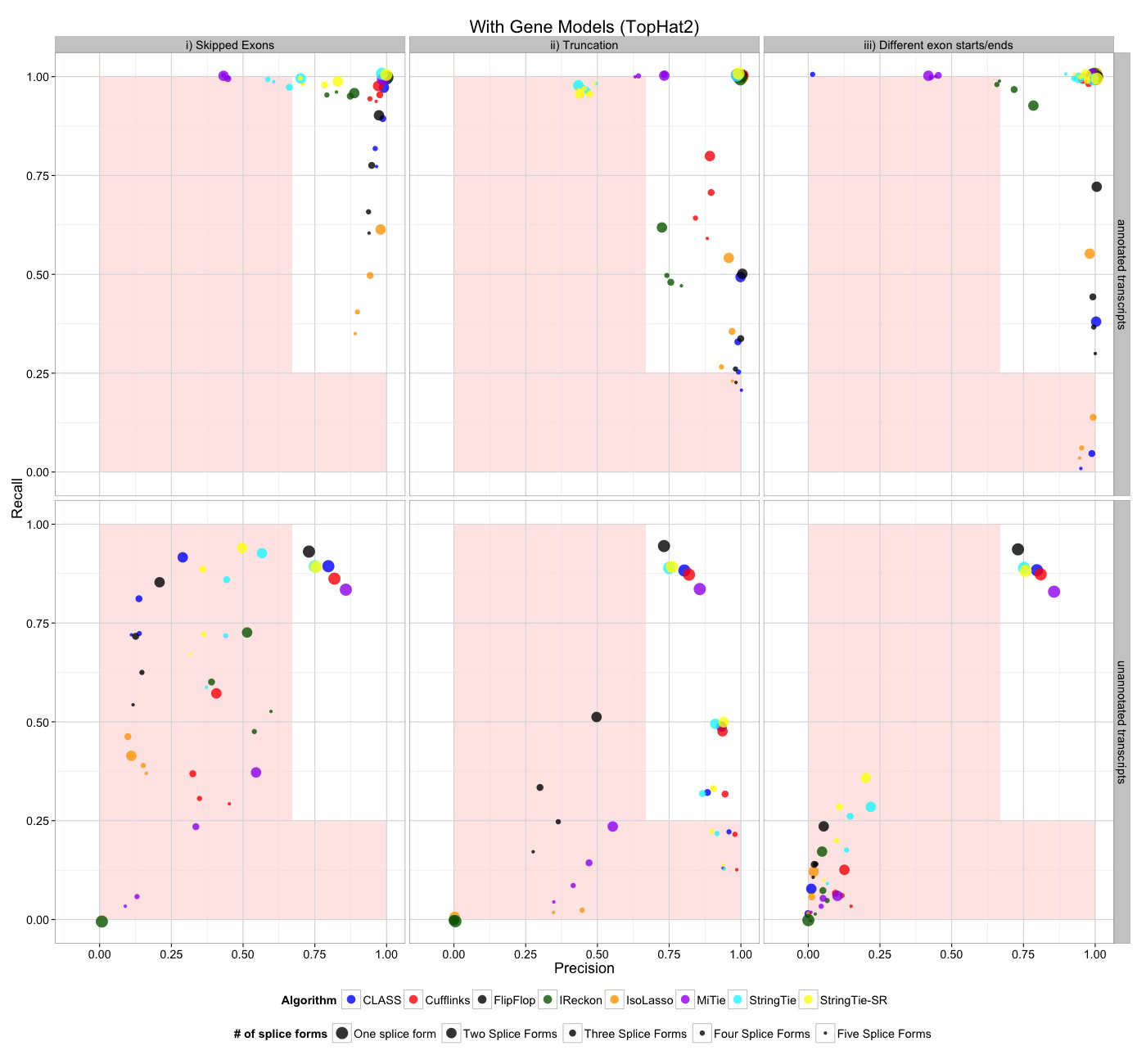

Supplement: Supplementary Data [file supp_btv488_suppl_data.zip › figS15.jpeg]

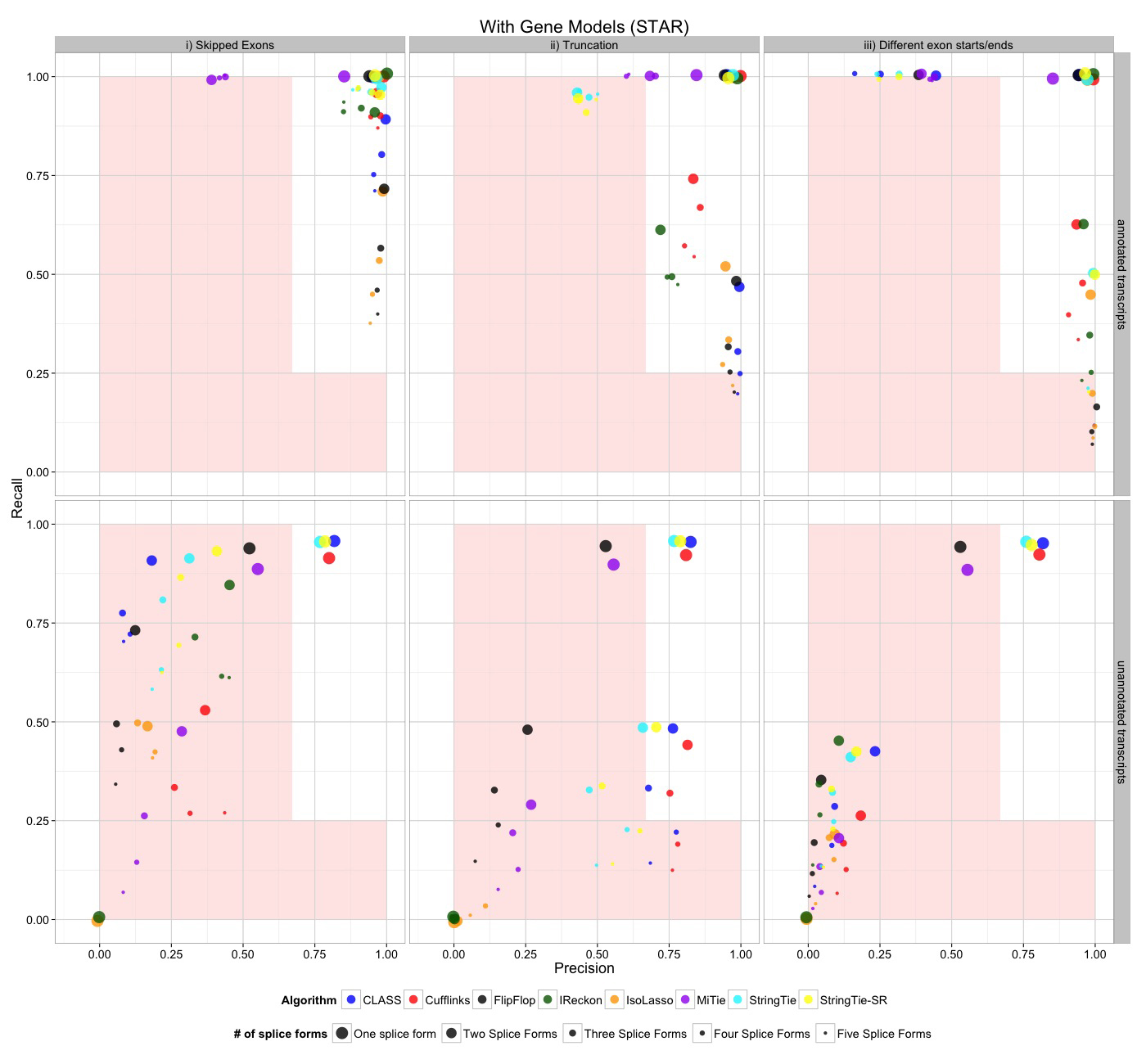

Supplement: Supplementary Data [file supp_btv488_suppl_data.zip › figS16.jpeg]

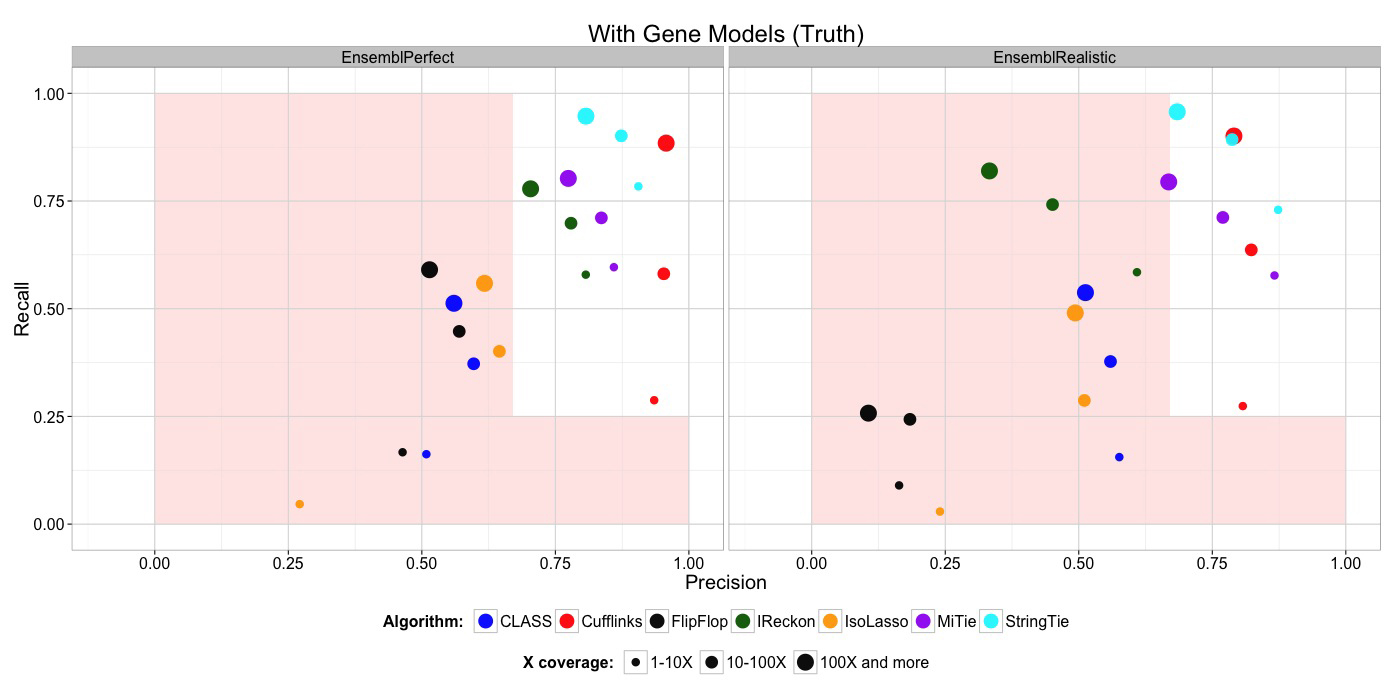

Supplement: Supplementary Data [file supp_btv488_suppl_data.zip › figS17.jpeg]
