# Supplementary material for: Benchmark analysis of algorithms for determining and quantifying full-length mRNA splice forms from RNA-seq data
Source: Bioinformatics. 2015 Sep 3;31(24):3938–45. doi: 10.1093/bioinformatics/btv488 (PMC4673975; doi:10.1093/bioinformatics/btv488)
Supplement: Supplementary Data [file supp_btv488_suppl_data.zip › hayer_bioinf3_Supplemental.doc]

Gene Expression

Supplemental Information for Benchmark Analysis of Algorithms for Determining and Quantifying Full-length mRNA Splice Forms from RNA-Seq Data

# 1 Overview

Table S1 – Summary for DataSet T1

| **DateSet T1** | **Alingment** |  |  |  | **Algorithm Settings** |  | **Algorithms** |  |  | **FlipFlop** |  |
| --- | --- | --- | --- | --- | --- | --- | --- | --- | --- | --- | --- |
| **Figure** | **Truth** | **TopHat2** | **STAR** | **With Gene Models** | **With Gene Models** | **Split output** | **Augustus** | **CLASS** | **Cufflinks** |  |  |
| **Figure 4 Left** | **✔** |  |  | **✔** | **✔** | **✔** |  | **✔** | **✔** | **✔** |  |
| **Figure 4 Middle** | **✔** |  |  | **✔** | **✔** | **✔** |  | **✔** | **✔** | **✔** |  |
| **Figure 4 Right** | **✔** |  |  | **✔** | **✔** | **✔** |  | **✔** | **✔** | **✔** |  |
| **Figure S1 Left** | **✔** |  |  |  |  |  | **✔** | **✔** | **✔** | **✔** |  |
| **Figure S1 Midldle** | **✔** |  |  |  |  |  | **✔** | **✔** | **✔** | **✔** |  |
| **Figure S1 Right** | **✔** |  |  |  |  |  | **✔** | **✔** | **✔** | **✔** |  |
| **Figure S2 Left** |  | **✔** |  | **✔** | **✔** |  |  | **✔** | **✔** | **✔** |  |
| **Figure S2 Middle** |  | **✔** |  | **✔** | **✔** |  |  | **✔** | **✔** | **✔** |  |
| **Figure S2 Right** |  | **✔** |  | **✔** | **✔** |  |  | **✔** | **✔** | **✔** |  |
| **Figure S3 Left** |  | **✔** |  |  |  |  | **✔** | **✔** | **✔** | **✔** |  |
| **Figure S3 Middle** |  | **✔** |  |  |  |  | **✔** | **✔** | **✔** | **✔** |  |
| **Figure S3 Right** |  | **✔** |  |  |  |  | **✔** | **✔** | **✔** | **✔** |  |
| **Figure S4 Left** |  |  | **✔** | **✔** | **✔** |  |  | **✔** | **✔** | **✔** |  |
| **Figure S4 Middle** |  |  | **✔** | **✔** | **✔** |  |  | **✔** | **✔** | **✔** |  |
| **Figure S4 Right** |  |  | **✔** | **✔** | **✔** |  |  | **✔** | **✔** | **✔** |  |
| **Figure S5 Left** |  |  | **✔** |  |  |  | **✔** | **✔** | **✔** | **✔** |  |
| **Figure S5 Middle** |  |  | **✔** |  |  |  | **✔** | **✔** | **✔** | **✔** |  |
| **Figure S5 Right** |  |  | **✔** |  |  |  | **✔** | **✔** | **✔** | **✔** |  |
| **Figure S6 Left** |  |  |  |  |  |  |  |  |  |  |  |
| **Figure S6 Middle** |  |  |  |  |  |  |  |  |  |  |  |
| **Figure S6 Right** |  |  |  |  |  |  |  |  |  |  |  |
| **Figure S14 Left** | **✔** |  |  | **✔** | **✔** |  |  | **✔** | **✔** | **✔** |  |
| **Figure S14 Middle** | **✔** |  |  | **✔** | **✔** |  |  | **✔** | **✔** | **✔** |  |
| **Figure S14 Right** | **✔** |  |  | **✔** | **✔** |  |  | **✔** | **✔** | **✔** |  |
| **Figure S15 Left** |  | **✔** |  | **✔** | **✔** | **✔** |  | **✔** | **✔** | **✔** |  |
| **Figure S15 Middle** |  | **✔** |  | **✔** | **✔** | **✔** |  | **✔** | **✔** | **✔** |  |
| **Figure S15 Right** |  | **✔** |  | **✔** | **✔** | **✔** |  | **✔** | **✔** | **✔** |  |
| **Figure S16 Left** |  |  | **✔** | **✔** | **✔** | **✔** |  | **✔** | **✔** | **✔** |  |
| **Figure S16 Middle** |  |  | **✔** | **✔** | **✔** | **✔** |  | **✔** | **✔** | **✔** |  |
| **Figure S16 Right** |  |  | **✔** | **✔** | **✔** | **✔** |  | **✔** | **✔** | **✔** |  |
| **DateSet T1** | **Algorithms** |  |  |  |  |  |  |  | **Splice Events** |  |  |
| **Figure** | **Ireckon** | **Isolasso** | **MiTie** | **SOAP** | **StringTie** | **StringTie-SR** | **Trans-ABySS** | **Trinity** | **Different exon starts/ends** | **Skipped Exons** | **Truncation** |
| **Figure 4 Left** | **✔** | **✔** | **✔** |  | **✔** |  |  |  | **✔** |  |  |
| **Figure 4 Middle** | **✔** | **✔** | **✔** |  | **✔** |  |  |  |  | **✔** |  |
| **Figure 4 Right** | **✔** | **✔** | **✔** |  | **✔** |  |  |  |  |  | **✔** |
| **Figure S1 Left** |  | **✔** | **✔** |  | **✔** |  |  | **✔** | **✔** |  |  |
| **Figure S1 Midldle** |  | **✔** | **✔** |  | **✔** |  |  | **✔** |  | **✔** |  |
| **Figure S1 Right** |  | **✔** | **✔** |  | **✔** |  |  | **✔** |  |  | **✔** |
| **Figure S2 Left** | **✔** | **✔** | **✔** |  | **✔** | **✔** |  |  | **✔** |  |  |
| **Figure S2 Middle** | **✔** | **✔** | **✔** |  | **✔** | **✔** |  |  |  | **✔** |  |
| **Figure S2 Right** | **✔** | **✔** | **✔** |  | **✔** | **✔** |  |  |  |  | **✔** |
| **Figure S3 Left** |  | **✔** | **✔** |  | **✔** | **✔** |  | **✔** | **✔** |  |  |
| **Figure S3 Middle** |  | **✔** | **✔** |  | **✔** | **✔** |  | **✔** |  | **✔** |  |
| **Figure S3 Right** |  | **✔** | **✔** |  | **✔** | **✔** |  | **✔** |  |  | **✔** |
| **Figure S4 Left** | **✔** | **✔** | **✔** |  | **✔** | **✔** |  |  | **✔** |  |  |
| **Figure S4 Middle** | **✔** | **✔** | **✔** |  | **✔** | **✔** |  |  |  | **✔** |  |
| **Figure S4 Right** | **✔** | **✔** | **✔** |  | **✔** | **✔** |  |  |  |  | **✔** |
| **Figure S5 Left** |  | **✔** | **✔** |  | **✔** | **✔** |  | **✔** | **✔** |  |  |
| **Figure S5 Middle** |  | **✔** | **✔** |  | **✔** | **✔** |  | **✔** |  | **✔** |  |
| **Figure S5 Right** |  | **✔** | **✔** |  | **✔** | **✔** |  | **✔** |  |  | **✔** |
| **Figure S6 Left** |  |  |  | **✔** |  |  | **✔** | **✔** | **✔** |  |  |
| **Figure S6 Middle** |  |  |  | **✔** |  |  | **✔** | **✔** |  | **✔** |  |
| **Figure S6 Right** |  |  |  | **✔** |  |  | **✔** | **✔** |  |  | **✔** |
| **Figure S14 Left** | **✔** | **✔** | **✔** |  | **✔** |  |  |  | **✔** |  |  |
| **Figure S14 Middle** | **✔** | **✔** | **✔** |  | **✔** |  |  |  |  | **✔** |  |
| **Figure S14 Right** | **✔** | **✔** | **✔** |  | **✔** |  |  |  |  |  | **✔** |
| **Figure S15 Left** | **✔** | **✔** | **✔** |  | **✔** | **✔** |  |  | **✔** |  |  |
| **Figure S15 Middle** | **✔** | **✔** | **✔** |  | **✔** | **✔** |  |  |  | **✔** |  |
| **Figure S15 Right** | **✔** | **✔** | **✔** |  | **✔** | **✔** |  |  |  |  | **✔** |
| **Figure S16 Left** | **✔** | **✔** | **✔** |  | **✔** | **✔** |  |  | **✔** |  |  |
| **Figure S16 Middle** | **✔** | **✔** | **✔** |  | **✔** | **✔** |  |  |  | **✔** |  |
| **Figure S16 Right** | **✔** | **✔** | **✔** |  | **✔** | **✔** |  |  |  |  | **✔** |

Table S2 - Summary for DataSet EP and ER

| **EP and ER** | **Alingment** |  |  |  | **Algorithm Settings** |  | **Algorithms** |  |  |  |
| --- | --- | --- | --- | --- | --- | --- | --- | --- | --- | --- |
| **Figure** | **Truth** | **TopHat2** | **STAR** | **With Gene Models** | **With Gene Models** | **Split output** | **Augustus** | **CLASS** | **Cufflinks** | **FlipFlop** |
| Figure 5 Left | ✔ |  |  | ✔ | ✔ | ✔ |  | ✔ | ✔ | ✔ |
| Figure 5 Right | ✔ |  |  | ✔ | ✔ | ✔ |  | ✔ | ✔ | ✔ |
| Figure 7 Left | ✔ |  |  | ✔ | ✔ |  |  | ✔ | ✔ | ✔ |
| Figure 7 Middle | ✔ |  |  | ✔ | ✔ |  |  | ✔ | ✔ | ✔ |
| Figure 7 Right | ✔ |  |  | ✔ | ✔ |  |  | ✔ | ✔ | ✔ |
| Figure S7 Left | ✔ |  |  |  |  |  | ✔ | ✔ | ✔ | ✔ |
| Figure S7 Right | ✔ |  |  |  |  |  | ✔ | ✔ | ✔ | ✔ |
| Figure S8 Left |  | ✔ |  | ✔ | ✔ |  |  | ✔ | ✔ | ✔ |
| Figure S8 Right |  | ✔ |  | ✔ | ✔ |  |  | ✔ | ✔ | ✔ |
| Figure S9 Left |  | ✔ |  |  |  |  | ✔ | ✔ | ✔ | ✔ |
| Figure S9 Right |  | ✔ |  |  |  |  | ✔ | ✔ | ✔ | ✔ |
| Figure S10 Left |  |  | ✔ | ✔ | ✔ |  |  | ✔ | ✔ | ✔ |
| Figure S10 Right |  |  | ✔ | ✔ | ✔ |  |  | ✔ | ✔ | ✔ |
| Figure S11 Left |  |  | ✔ |  |  |  | ✔ | ✔ | ✔ | ✔ |
| Figure S11 Right |  |  | ✔ |  |  |  | ✔ | ✔ | ✔ | ✔ |
| Figure S12 Left |  |  |  |  |  |  |  |  |  |  |
| Figure S12 Right |  |  |  |  |  |  |  |  |  |  |
| Figure S17 Left | ✔ |  |  | ✔ | ✔ |  |  | ✔ | ✔ | ✔ |
| Figure S17 Right | ✔ |  |  | ✔ | ✔ |  |  | ✔ | ✔ | ✔ |
| Figure S18 Left |  | ✔ |  | ✔ | ✔ | ✔ |  | ✔ | ✔ | ✔ |
| Figure S18 Right |  | ✔ |  | ✔ | ✔ | ✔ |  | ✔ | ✔ | ✔ |
| Figure S10 Left |  |  | ✔ | ✔ | ✔ | ✔ |  | ✔ | ✔ | ✔ |
| Figure S10 Right |  |  | ✔ | ✔ | ✔ | ✔ |  | ✔ | ✔ | ✔ |
| **EP and ER** |  |  |  |  |  |  |  |  | **Dataset** |  |
| **Figure** | **Ireckon** | **Isolasso** | **MiTie** | **SOAP** | **StringTie** | **StringTie-SR** | **Trans-ABySS** | **Trinity** | **EnsemblPerfect** | **EnsemblRealistic** |
| Figure 5 Left | ✔ | ✔ | ✔ |  | ✔ |  |  |  | ✔ |  |
| Figure 5 Right | ✔ | ✔ | ✔ |  | ✔ |  |  |  |  | ✔ |
| Figure 7 Left | ✔ | ✔ | ✔ |  | ✔ |  |  |  |  | ✔ |
| Figure 7 Middle | ✔ | ✔ | ✔ |  | ✔ |  |  |  |  | ✔ |
| Figure 7 Right | ✔ | ✔ | ✔ |  | ✔ |  |  |  |  | ✔ |
| Figure S7 Left |  | ✔ | ✔ |  | ✔ |  |  | ✔ | ✔ |  |
| Figure S7 Right |  | ✔ | ✔ |  | ✔ |  |  | ✔ |  | ✔ |
| Figure S8 Left | ✔ | ✔ | ✔ |  | ✔ | ✔ |  |  | ✔ |  |
| Figure S8 Right | ✔ | ✔ | ✔ |  | ✔ | ✔ |  |  |  | ✔ |
| Figure S9 Left |  | ✔ | ✔ |  | ✔ | ✔ |  | ✔ | ✔ |  |
| Figure S9 Right |  | ✔ | ✔ |  | ✔ | ✔ |  | ✔ |  | ✔ |
| Figure S10 Left | ✔ | ✔ | ✔ |  | ✔ | ✔ |  |  | ✔ |  |
| Figure S10 Right | ✔ | ✔ | ✔ |  | ✔ | ✔ |  |  |  | ✔ |
| Figure S11 Left |  | ✔ | ✔ |  | ✔ | ✔ |  | ✔ | ✔ |  |
| Figure S11 Right |  | ✔ | ✔ |  | ✔ | ✔ |  | ✔ |  | ✔ |
| Figure S12 Left |  |  |  | ✔ |  |  | ✔ | ✔ | ✔ |  |
| Figure S12 Right |  |  |  | ✔ |  |  | ✔ | ✔ |  | ✔ |
| Figure S17 Left | ✔ | ✔ | ✔ |  | ✔ |  |  |  | ✔ |  |
| Figure S17 Right | ✔ | ✔ | ✔ |  | ✔ |  |  |  |  | ✔ |
| Figure S18 Left | ✔ | ✔ | ✔ |  | ✔ | ✔ |  |  | ✔ |  |
| Figure S18 Right | ✔ | ✔ | ✔ |  | ✔ | ✔ |  |  |  | ✔ |
| Figure S10 Left | ✔ | ✔ | ✔ |  | ✔ | ✔ |  |  | ✔ |  |
| Figure S10 Right | ✔ | ✔ | ✔ |  | ✔ | ✔ |  |  |  | ✔ |

Table S3 - Summary for DataSet IVT

| **IVT** | **Alingment** |  |  |  | **Algorithm Settings** | **Algorithms** |  |  |  |  |
| --- | --- | --- | --- | --- | --- | --- | --- | --- | --- | --- |
| **Figures** | **Truth** | **TopHat2** | **STAR** | **With Gene Models** | **With Gene Models** | **Augustus** | **CLASS** | **Cufflinks** | **FlipFlop** | **Ireckon** |
| Figures 6 Left |  | ✔ | ✔ | ✔ | ✔ |  | ✔ | ✔ | ✔ | ✔ |
| Figures 6 Middle |  | ✔ | ✔ | ✔ | ✔ |  | ✔ | ✔ | ✔ | ✔ |
| Figures 6 Right | ✔ | ✔ | ✔ | ✔ | ✔ |  | ✔ | ✔ | ✔ | ✔ |
| Figures S13 Left |  | ✔ | ✔ |  |  | ✔ | ✔ | ✔ | ✔ |  |
| Figures S13 Middle |  | ✔ | ✔ |  |  | ✔ | ✔ | ✔ | ✔ |  |
| Figures S13 Right | ✔ | ✔ | ✔ |  |  | ✔ | ✔ | ✔ | ✔ |  |
| **IVT** |  |  |  |  |  |  |  | **Dataset** |  |  |
| **Figures** | **Isolasso** | **MiTie** | **SOAP** | **StringTie** | **StringTie-SR** | **Trans-ABySS** | **Trinity** | **PolyA** | **Ribo** | **Simulated** |
| Figures 6 Left | ✔ | ✔ |  | ✔ | ✔ |  |  | ✔ |  |  |
| Figures 6 Middle | ✔ | ✔ |  | ✔ | ✔ |  |  |  | ✔ |  |
| Figures 6 Right | ✔ | ✔ |  | ✔ |  |  |  |  |  | ✔ |
| Figures S13 Left | ✔ | ✔ | ✔ | ✔ | ✔ | ✔ | ✔ | ✔ |  |  |
| Figures S13 Middle | ✔ | ✔ | ✔ | ✔ | ✔ | ✔ | ✔ |  | ✔ |  |
| Figures S13 Right | ✔ | ✔ | ✔ | ✔ |  | ✔ | ✔ |  |  | ✔ |

# 2 Supplemental Figures

**Fig. S1.** Accuracy statistics for simulated data set T1. True alignment. Analysis performed without gene models.

**Fig. S2.** Accuracy statistics for simulated data set T1. TopHat alignment. Analysis performed with gene models.

**Fig. S3.** Accuracy statistics for simulated data set T1. TopHat alignment. Analysis performed without gene models.

**Fig. S4.** Accuracy statistics for simulated data set T1. STAR alignment. Analysis performed with gene models.

**Fig. S5.** Accuracy statistics for simulated data set T1. STAR alignment. Analysis performed without gene models.

**Fig. S6.** Accuracy statistics for simulated data set T1. De novo methods.

**Fig. S7.**  Accuracy statistics for simulated data sets EP and ER. True alignment. Analysis performed without gene models.

**Fig. S8.**  Accuracy statistics for simulated data sets EP and ER. TopHat alignment. Analysis performed with gene models.

**Fig. S9.**  Accuracy statistics for simulated data sets EP and ER. TopHat alignment. Analysis performed without gene models.

**Fig. S10.**  Accuracy statistics for simulated data sets EP and ER. STAR alignment. Analysis performed with gene models.

**Fig. S11.**  Accuracy statistics for simulated data sets EP and ER. STAR alignment. Analysis performed without gene models.

**Fig. S12.**  Accuracy statistics for simulated data sets EP and ER. De novo methods.

**Fig. S13.**  Accuracy statistics for the IVT data. Analysis performed without gene models.

**Fig. S14.**  Accuracy statistics for simulated data set T1. True alignment. Analysis performed with gene models.

**Fig. S15.**  Accuracy statistics for simulated data set T1. TopHat alignment. Analysis performed with gene models. The metrics are divided into “annotated transcripts” and “unannotated transcripts”. The “annotated transcripts” include the results for expressed and unexpressed simulated transcripts for which the structure was given to the algorithm. Whereas a false-positive in the lower panels is an inferred (unannotated) transcript that is not expressed and a false-negative is any of the 15% of hidden transcripts that are expressed that were not called as expressed.

**Fig. S16.**  Accuracy statistics for simulated data set T1. STAR alignment. Analysis performed with gene models. The metrics are divided into “annotated transcripts” and “unannotated transcripts”. The “annotated transcripts” include the results for expressed and unexpressed simulated transcripts for which the structure was given to the algorithm. Whereas a false-positive in the lower panels is an inferred (unannotated) transcript that is not expressed and a false-negative is any of the 15% of hidden transcripts that are expressed that were not called as expressed.

**Fig. S17.**  Accuracy statistics for simulated data sets EP and ER. True alignment. Analysis performed with gene models.

**Fig. S18.**  Accuracy statistics for simulated data sets EP and ER. TopHat alignment. Analysis performed with gene models. The metrics are divided into “annotated transcripts” and “unannotated transcripts”. The “annotated transcripts” include the results for expressed and unexpressed simulated transcripts for which the structure was given to the algorithm. Whereas a false-positive in the lower panels is an inferred (unannotated) transcript that is not expressed and a false-negative is any of the 15% of hidden transcripts that are expressed that were not called as expressed.

**Fig. S19.**  Accuracy statistics for simulated data sets EP and ER. STAR alignment. Analysis performed with gene models. The metrics are divided into “annotated transcripts” and “unannotated transcripts”. The “annotated transcripts” include the results for expressed and unexpressed simulated transcripts for which the structure was given to the algorithm. Whereas a false-positive in the lower panels is an inferred (unannotated) transcript that is not expressed and a false-negative is any of the 15% of hidden transcripts that are expressed that were not called as expressed.

# 3 Supplemtal Tables

Table S4 – Values used to generate figure 4

| **DataSet T1** | | |  |  |  |  |  |  |  |  |
| --- | --- | --- | --- | --- | --- | --- | --- | --- | --- | --- |
| **Figure 4** | **With Gene Models (Truth) - Split by annotation** | | | | | | | | | |
|  |  |  |  |  |  |  |  |  |  |  |
| **Left** | **Different exon starts/ends** | | | |  |  |  |  |  | |
|  |  |  |  |  |  |  |  |  |  |  |
| Recall | One Splice Form | Two Splice Forms | Three Splice Forms | Four Splice Forms | Five Splice Forms | One Splice Form Annotated | Two Splice Forms Annotated | Three Splice Forms Annotated | Four Splice Forms Annotated | Five Splice Forms Annotated |
| CLASS | 0.986 | 0.202 | 0.008 | 0.002 | 0.000 | 1.000 | 0.203 | 1.000 | 1.000 | 1.000 |
| Cufflinks | 0.994 | 0.492 | 0.276 | 0.168 | 0.165 | 1.000 | 0.953 | 0.967 | 0.960 | 0.971 |
| FlipFlop | 1.000 | 0.628 | 0.408 | 0.356 | 0.264 | 1.000 | 0.639 | 0.411 | 0.334 | 0.270 |
| IReckon | 0.000 | 0.580 | 0.310 | 0.227 | 0.136 | 1.000 | 0.841 | 0.919 | 0.943 | 0.970 |
| IsoLasso | 0.000 | 0.140 | 0.054 | 0.024 | 0.004 | 1.000 | 0.577 | 0.151 | 0.070 | 0.032 |
| MiTie | 0.852 | 0.404 | 0.296 | 0.082 | 0.052 | 1.000 | 0.824 | 0.796 | 0.813 | 0.849 |
| StringTie | 0.988 | 0.456 | 0.310 | 0.220 | 0.145 | 1.000 | 1.000 | 0.378 | 0.312 | 0.293 |
|  |  |  |  |  |  |  |  |  |  |  |
| Precision | One Splice Form | Two Splice Forms | Three Splice Forms | Four Splice Forms | Five Splice Forms | One Splice Form Annotated | Two Splice Forms Annotated | Three Splice Forms Annotated | Four Splice Forms Annotated | Five Splice Forms Annotated |
| CLASS | 0.920 | 0.027 | 0.001 | 0.001 | 0.000 | 0.990 | 0.997 | 0.013 | 0.002 | 0.000 |
| Cufflinks | 0.984 | 0.272 | 0.173 | 0.189 | 0.269 | 1.000 | 0.980 | 0.992 | 0.975 | 0.993 |
| FlipFlop | 0.724 | 0.115 | 0.033 | 0.037 | 0.023 | 1.000 | 0.999 | 0.999 | 0.997 | 0.998 |
| IReckon | 0.000 | 0.241 | 0.115 | 0.134 | 0.151 | 0.998 | 0.912 | 0.777 | 0.721 | 0.703 |
| IsoLasso | NA | 0.037 | 0.009 | 0.008 | 0.001 | 1.000 | 0.983 | 0.987 | 0.955 | 0.962 |
| MiTie | 0.253 | 0.139 | 0.086 | 0.036 | 0.021 | 0.894 | 0.698 | 0.748 | 0.690 | 0.709 |
| StringTie | 0.945 | 0.159 | 0.062 | 0.064 | 0.042 | 0.992 | 0.557 | 0.998 | 0.999 | 0.980 |
|  |  |  |  |  |  |  |  |  |  |  |
| **Middle** | **Skipped Exons** | | | | | |  |  |  |  |
|  |  |  |  |  |  |  |  |  |  |  |
| Recall | One Splice Form | Two Splice Forms | Three Splice Forms | Four Splice Forms | Five Splice Forms | One Splice Form Annotated | Two Splice Forms Annotated | Three Splice Forms Annotated | Four Splice Forms Annotated | Five Splice Forms Annotated |
| CLASS | 0.986 | 0.988 | 0.898 | 0.822 | 0.782 | 1.000 | 0.975 | 0.908 | 0.838 | 0.796 |
| Cufflinks | 0.994 | 0.746 | 0.510 | 0.420 | 0.354 | 1.000 | 0.968 | 0.962 | 0.947 | 0.933 |
| FlipFlop | 1.000 | 0.914 | 0.752 | 0.660 | 0.568 | 1.000 | 0.901 | 0.783 | 0.672 | 0.605 |
| IReckon | 0.000 | 0.866 | 0.730 | 0.618 | 0.625 | 1.000 | 0.919 | 0.928 | 0.932 | 0.936 |
| IsoLasso | 0.000 | 0.524 | 0.544 | 0.430 | 0.409 | 1.000 | 0.731 | 0.558 | 0.453 | 0.388 |
| MiTie | 0.852 | 0.478 | 0.334 | 0.148 | 0.075 | 1.000 | 0.851 | 0.763 | 0.681 | 0.658 |
| StringTie | 0.988 | 0.980 | 0.954 | 0.808 | 0.703 | 1.000 | 0.988 | 0.987 | 0.990 | 0.988 |
|  |  |  |  |  |  |  |  |  |  |  |
| Precision | One Splice Form | Two Splice Forms | Three Splice Forms | Four Splice Forms | Five Splice Forms | One Splice Form Annotated | Two Splice Forms Annotated | Three Splice Forms Annotated | Four Splice Forms Annotated | Five Splice Forms Annotated |
| CLASS | 0.920 | 0.338 | 0.154 | 0.165 | 0.126 | 0.990 | 0.991 | 0.982 | 0.969 | 0.973 |
| Cufflinks | 0.984 | 0.531 | 0.349 | 0.411 | 0.450 | 1.000 | 0.973 | 0.982 | 0.964 | 0.974 |
| FlipFlop | 0.724 | 0.229 | 0.130 | 0.161 | 0.127 | 1.000 | 0.988 | 0.974 | 0.974 | 0.963 |
| IReckon | 0.000 | 0.475 | 0.355 | 0.471 | 0.470 | 0.998 | 0.964 | 0.910 | 0.843 | 0.860 |
| IsoLasso | NA | 0.191 | 0.145 | 0.195 | 0.184 | 1.000 | 0.988 | 0.979 | 0.956 | 0.960 |
| MiTie | 0.253 | 0.192 | 0.157 | 0.126 | 0.071 | 0.894 | 0.687 | 0.712 | 0.651 | 0.669 |
| StringTie | 0.945 | 0.542 | 0.404 | 0.418 | 0.340 | 0.992 | 0.978 | 0.947 | 0.909 | 0.892 |
|  |  |  |  |  |  |  |  |  |  |  |
| **Right** | **Truncation** | | |  |  |  |  |  |  |  |
|  |  |  |  |  |  |  |  |  |  |  |
| Recall | One Splice Form | Two Splice Forms | Three Splice Forms | Four Splice Forms | Five Splice Forms | One Splice Form Annotated | Two Splice Forms Annotated | Three Splice Forms Annotated | Four Splice Forms Annotated | Five Splice Forms Annotated |
| CLASS | 0.986 | 0.504 | 0.340 | 0.243 | 0.141 | 1.000 | 1.000 | 0.328 | 1.000 | 0.212 |
| Cufflinks | 0.994 | 0.510 | 1.000 | 1.000 | 1.000 | 1.000 | 0.797 | 0.707 | 0.639 | 0.591 |
| FlipFlop | 1.000 | 0.522 | 0.346 | 0.260 | 0.162 | 1.000 | 0.497 | 0.342 | 0.267 | 0.232 |
| IReckon | 0.000 | 0.000 | 0.000 | 0.000 | 0.000 | 1.000 | 0.613 | 0.484 | 0.499 | 0.472 |
| IsoLasso | 0.000 | 0.000 | 0.000 | 0.029 | 0.014 | 1.000 | 0.537 | 0.350 | 0.273 | 0.226 |
| MiTie | 0.852 | 0.276 | 0.202 | 0.116 | 0.075 | 1.000 | 0.801 | 0.688 | 0.631 | 0.582 |
| StringTie | 0.988 | 0.514 | 0.342 | 0.246 | 0.144 | 1.000 | 0.987 | 0.986 | 0.989 | 0.986 |
|  |  |  |  |  |  |  |  |  |  |  |
| Precision | One Splice Form | Two Splice Forms | Three Splice Forms | Four Splice Forms | Five Splice Forms | One Splice Form Annotated | Two Splice Forms Annotated | Three Splice Forms Annotated | Four Splice Forms Annotated | Five Splice Forms Annotated |
| CLASS | 0.920 | 0.926 | 0.924 | 0.976 | 0.870 | 0.990 | 0.490 | 0.996 | 0.251 | 0.999 |
| Cufflinks | 0.984 | 0.992 | 0.340 | 0.242 | 0.143 | 1.000 | 0.886 | 0.900 | 0.849 | 0.885 |
| FlipFlop | 0.724 | 0.501 | 0.299 | 0.335 | 0.250 | 1.000 | 0.999 | 0.995 | 0.983 | 0.988 |
| IReckon | 0.000 | NA | NA | NA | NA | 0.998 | 0.725 | 0.763 | 0.744 | 0.788 |
| IsoLasso | NA | 0.000 | 0.000 | 0.460 | 0.350 | 1.000 | 0.953 | 0.962 | 0.933 | 0.967 |
| MiTie | 0.253 | 0.122 | 0.096 | 0.117 | 0.091 | 0.894 | 0.597 | 0.623 | 0.572 | 0.611 |
| StringTie | 0.945 | 0.966 | 0.919 | 0.957 | 0.894 | 0.992 | 0.427 | 0.470 | 0.443 | 0.494 |

Table S4 - Values used to generate figure 5

| **DataSet EP and ER** | |  |  |  |  |  |
| --- | --- | --- | --- | --- | --- | --- |
| **Figure 5** | **With Gene Models (Truth) - Split by annotation** | | | | |  |
|  |  |  |  |  |  |  |
| **Left** | **EnsemblPerfect** | |  |  |  |  |
|  |  |  |  |  |  |  |
| Recall | 0-10X | 10-100X | 100X_and_more | 0-10X_annotated | 10-100X_annotated | 100X_and_more_annotated |
| CLASS | 0.159 | 0.368 | 0.513 | 0.170 | 0.380 | 0.515 |
| Cufflinks | 0.145 | 0.398 | 0.613 | 0.321 | 0.618 | 0.935 |
| FlipFlop | 0.016 | 0.067 | 0.129 | 0.203 | 0.506 | 0.673 |
| IReckon | 0.109 | 0.178 | 0.245 | 0.656 | 0.790 | 0.864 |
| IsoLasso | 0.018 | 0.082 | 0.142 | 0.060 | 0.454 | 0.629 |
| MiTie | 0.063 | 0.175 | 0.439 | 0.698 | 0.814 | 0.868 |
| StringTie | 0.172 | 0.430 | 0.668 | 0.898 | 0.993 | 0.994 |
|  |  |  |  |  |  |  |
| Precision | 0-10X | 10-100X | 100X_and_more | 0-10X_annotated | 10-100X_annotated | 100X_and_more_annotated |
| CLASS | 0.515 | 0.569 | 0.605 | 0.504 | 0.608 | 0.558 |
| Cufflinks | 0.911 | 0.933 | 0.932 | 0.945 | 0.958 | 0.956 |
| FlipFlop | 0.077 | 0.156 | 0.219 | 0.489 | 0.601 | 0.543 |
| IReckon | 0.498 | 0.447 | 0.459 | 0.828 | 0.796 | 0.720 |
| IsoLasso | 0.134 | 0.264 | 0.333 | 0.294 | 0.678 | 0.636 |
| MiTie | 0.479 | 0.513 | 0.689 | 0.873 | 0.848 | 0.783 |
| StringTie | 0.714 | 0.745 | 0.768 | 0.909 | 0.882 | 0.813 |
|  |  |  |  |  |  |  |
| **Right** | **EnsemblRealistic** | |  |  |  |  |
|  |  |  |  |  |  |  |
| Recall | 0-10X | 10-100X | 100X_and_more | 0-10X_annotated | 10-100X_annotated | 100X_and_more_annotated |
| CLASS | 0.150 | 0.373 | 0.547 | 0.163 | 0.382 | 0.534 |
| Cufflinks | 0.118 | 0.407 | 0.634 | 0.299 | 0.671 | 0.956 |
| FlipFlop | 0.017 | 0.035 | 0.044 | 0.101 | 0.281 | 0.298 |
| IReckon | 0.124 | 0.215 | 0.259 | 0.661 | 0.838 | 0.918 |
| IsoLasso | 0.012 | 0.062 | 0.095 | 0.038 | 0.319 | 0.574 |
| MiTie | 0.029 | 0.101 | 0.308 | 0.670 | 0.818 | 0.883 |
| StringTie | 0.123 | 0.444 | 0.734 | 0.830 | 0.975 | 0.991 |
|  |  |  |  |  |  |  |
| Precision | 0-10X | 10-100X | 100X_and_more | 0-10X_annotated | 10-100X_annotated | 100X_and_more_annotated |
| CLASS | 0.534 | 0.546 | 0.550 | 0.592 | 0.568 | 0.504 |
| Cufflinks | 0.665 | 0.742 | 0.790 | 0.818 | 0.827 | 0.789 |
| FlipFlop | 0.032 | 0.035 | 0.026 | 0.195 | 0.210 | 0.123 |
| IReckon | 0.228 | 0.193 | 0.175 | 0.643 | 0.479 | 0.352 |
| IsoLasso | 0.090 | 0.192 | 0.207 | 0.267 | 0.537 | 0.524 |
| MiTie | 0.237 | 0.301 | 0.479 | 0.884 | 0.794 | 0.680 |
| StringTie | 0.542 | 0.638 | 0.692 | 0.891 | 0.797 | 0.687 |

Table S5 - Values used to generate figure 6

| **IVT** |  |  |  |
| --- | --- | --- | --- |
| **Figure 6** | **With Gene Models** | | |
|  |  |  |  |
| **Left** | **PolyA** |  |  |
|  |  |  |  |
| Recall | STAR | Tophat2 |  |
| CLASS | 0.095 | 0.125 |  |
| Cufflinks | 0.707 | 0.729 |  |
| FlipFlop | 0.683 | 0.731 |  |
| IReckon | 0.588 | 0.391 |  |
| IsoLasso | 0.027 | 0.700 |  |
| MiTie | 0.172 | 0.799 |  |
| StringTie | 0.723 | 0.758 |  |
| StringTie-SR | 0.723 | 0.758 |  |
|  |  |  |  |
| Precision | STAR | Tophat2 |  |
| CLASS | 0.216 | 0.710 |  |
| Cufflinks | 0.745 | 0.846 |  |
| FlipFlop | 0.228 | 0.264 |  |
| IReckon | 0.125 | 0.126 |  |
| IsoLasso | 0.737 | 0.844 |  |
| MiTie | 0.260 | 0.344 |  |
| StringTie | 0.652 | 0.812 |  |
| StringTie-SR | 0.656 | 0.810 |  |
|  |  |  |  |
| **Middle** | **Ribo** |  |  |
|  |  |  |  |
| Recall | STAR | Tophat2 |  |
| CLASS | 0.086 | 0.133 |  |
| Cufflinks | 0.734 | 0.732 |  |
| FlipFlop | 0.685 | 0.713 |  |
| IReckon | 0.548 | 0.453 |  |
| IsoLasso | 0.027 | 0.697 |  |
| MiTie | 0.770 | 0.803 |  |
| StringTie | 0.728 | 0.764 |  |
| StringTie-SR | 0.728 | 0.765 |  |
|  |  |  |  |
| Precision | STAR | Tophat2 |  |
| CLASS | 0.296 | 0.749 |  |
| Cufflinks | 0.735 | 0.837 |  |
| FlipFlop | 0.233 | 0.262 |  |
| IReckon | 0.192 | 0.259 |  |
| IsoLasso | 0.718 | 0.848 |  |
| MiTie | 0.289 | 0.356 |  |
| StringTie | 0.675 | 0.826 |  |
| StringTie-SR | 0.679 | 0.823 |  |
|  |  |  |  |
| **Right** | **Simulated** |  |  |
|  |  |  |  |
| Recall | STAR | Tophat2 | Truth |
| CLASS | 0.748 | 0.803 | 0.854 |
| Cufflinks | 0.836 | 0.830 | 0.839 |
| FlipFlop | 0.780 | 0.833 | 0.856 |
| IReckon | 0.612 | 0.285 | 0.627 |
| IsoLasso | 0.710 | 0.775 | 0.404 |
| MiTie | 0.871 | 0.877 | 0.891 |
| StringTie | 0.795 | 0.874 | 0.901 |
| StringTie-SR | 0.787 | 0.876 | NA |
|  |  |  |  |
| Precision | STAR | Tophat2 | Truth |
| CLASS | 0.785 | 0.849 | 0.893 |
| Cufflinks | 0.836 | 0.828 | 0.849 |
| FlipFlop | 0.455 | 0.564 | 0.619 |
| IReckon | 0.943 | 0.941 | 0.944 |
| IsoLasso | 0.751 | 0.862 | 0.905 |
| MiTie | 0.464 | 0.639 | 0.716 |
| StringTie | 0.789 | 0.830 | 0.839 |
| StringTie-SR | 0.775 | 0.832 | NA |

Table S6 - Values used to generate figure 7

| **DataSet EP and ER** | | |  |
| --- | --- | --- | --- |
| **Figure 7** | **With Gene Models (Truth)** | | |
|  |  |  | |
| **Left** |  |  | |
|  |  |  | |
| Algorithm | Pearson Correlation | | |
| Mitie | 0.160 |  | |
| StringTie | 0.507 |  | |
| FlipFlop | 0.532 |  | |
| IsoLasso | 0.692 |  | |
| iReckon | 0.803 |  | |
| CLASS | 0.856 |  | |
| Cufflinks | 0.922 |  | |
|  |  |  | |
| **Middle** |  |  | |
|  |  |  | |
| Algorithm | Falsely called expressed | Falsely called not expressed | |
| CLASS | 32,647 | 17,627 | |
| Cufflinks | 23,823 | 41,024 | |
| FlipFlop | 41,743 | 54,244 | |
| iReckon | 12,252 | 66,272 | |
| IsoLasso | 38,299 | 15,667 | |
| Mitie | 16,105 | 14,673 | |
| StringTie | 7,416 | 14,079 | |
|  |  |  | |
| **Right** |  |  | |
|  |  |  | |
| Algorithm | Correctly called expressed | | |
| CLASS | 18,127 |  | |
| Cufflinks | 30,002 |  | |
| FlipFlop | 11,088 |  | |
| iReckon | 38,451 |  | |
| IsoLasso | 12,950 |  | |
| Mitie | 37,208 |  | |
| StringTie | 46,499 |  | |

Table S7 - Values to generate figure S1

| **DataSet T1** |  |  |  |  |  |
| --- | --- | --- | --- | --- | --- |
| **Figure S1** | **Without Gene Models (Truth)** | | |  |  |
|  |  |  |  |  |  |
| **Left** | **Different exon starts/ends** | |  |  |  |
|  |  |  |  |  |  |
| Recall | One Splice Form | Two Splice Forms | Three Splice Forms | Four Splice Forms | Five Splice Forms |
| AUGUSTUS | 0.425 | 0.187 | 0.151 | 0.109 | 0.094 |
| CLASS | 0.991 | 0.254 | 0.024 | 0.004 | 0.000 |
| Cufflinks | 0.993 | 0.634 | 0.383 | 0.306 | 0.258 |
| FlipFlop | 0.970 | 0.606 | 0.392 | 0.329 | 0.257 |
| IsoLasso | 0.971 | 0.399 | 0.102 | 0.043 | 0.019 |
| MiTie | 0.863 | 0.256 | 0.082 | 0.044 | 0.026 |
| StringTie | 0.990 | 0.477 | 0.280 | 0.219 | 0.182 |
| Trinity | 0.929 | 0.342 | 0.201 | 0.149 | 0.114 |
|  |  |  |  |  |  |
| Precision | One Splice Form | Two Splice Forms | Three Splice Forms | Four Splice Forms | Five Splice Forms |
| AUGUSTUS | 0.688 | 0.510 | 0.497 | 0.467 | 0.485 |
| CLASS | 0.974 | 0.113 | 0.014 | 0.004 | 0.000 |
| Cufflinks | 0.953 | 0.459 | 0.296 | 0.282 | 0.342 |
| FlipFlop | 0.807 | 0.324 | 0.164 | 0.124 | 0.105 |
| IsoLasso | 0.986 | 0.285 | 0.097 | 0.052 | 0.028 |
| MiTie | 0.332 | 0.146 | 0.069 | 0.045 | 0.037 |
| StringTie | 0.991 | 0.452 | 0.268 | 0.233 | 0.226 |
| Trinity | 0.832 | 0.239 | 0.113 | 0.073 | 0.061 |
|  |  |  |  |  |  |
| **Middle** | **Skipped Exons** |  |  |  |  |
|  |  |  |  |  |  |
| Recall | One Splice Form | Two Splice Forms | Three Splice Forms | Four Splice Forms | Five Splice Forms |
| AUGUSTUS | 0.425 | 0.223 | 0.162 | 0.122 | 0.102 |
| CLASS | 0.991 | 0.982 | 0.908 | 0.836 | 0.796 |
| Cufflinks | 0.993 | 0.815 | 0.640 | 0.545 | 0.478 |
| FlipFlop | 0.970 | 0.846 | 0.745 | 0.635 | 0.573 |
| IsoLasso | 0.971 | 0.688 | 0.558 | 0.439 | 0.392 |
| MiTie | 0.863 | 0.803 | 0.426 | 0.314 | 0.255 |
| StringTie | 0.990 | 0.860 | 0.723 | 0.649 | 0.594 |
| Trinity | 0.929 | 0.703 | 0.664 | 0.606 | 0.562 |
|  |  |  |  |  |  |
| Precision | One Splice Form | Two Splice Forms | Three Splice Forms | Four Splice Forms | Five Splice Forms |
| AUGUSTUS | 0.688 | 0.371 | 0.299 | 0.317 | 0.314 |
| CLASS | 0.974 | 0.670 | 0.522 | 0.441 | 0.421 |
| Cufflinks | 0.953 | 0.711 | 0.559 | 0.531 | 0.547 |
| FlipFlop | 0.807 | 0.481 | 0.448 | 0.406 | 0.408 |
| IsoLasso | 0.986 | 0.539 | 0.495 | 0.470 | 0.486 |
| MiTie | 0.332 | 0.523 | 0.439 | 0.435 | 0.443 |
| StringTie | 0.991 | 0.704 | 0.550 | 0.481 | 0.449 |
| Trinity | 0.832 | 0.400 | 0.346 | 0.315 | 0.301 |
|  |  |  |  |  |  |
| **Right** | **Truncation** |  |  |  |  |
|  |  |  |  |  |  |
| Recall | One Splice Form | Two Splice Forms | Three Splice Forms | Four Splice Forms | Five Splice Forms |
| AUGUSTUS | 0.425 | 0.230 | 0.139 | 0.115 | 0.094 |
| CLASS | 0.991 | 0.493 | 0.332 | 0.248 | 0.197 |
| Cufflinks | 0.993 | 0.496 | 0.329 | 0.248 | 0.199 |
| FlipFlop | 0.970 | 0.484 | 0.328 | 0.255 | 0.208 |
| IsoLasso | 0.971 | 0.481 | 0.323 | 0.244 | 0.193 |
| MiTie | 0.863 | 0.455 | 0.312 | 0.244 | 0.201 |
| StringTie | 0.990 | 0.497 | 0.331 | 0.250 | 0.198 |
| Trinity | 0.929 | 0.479 | 0.313 | 0.235 | 0.191 |
|  |  |  |  |  |  |
| Precision | One Splice Form | Two Splice Forms | Three Splice Forms | Four Splice Forms | Five Splice Forms |
| AUGUSTUS | 0.688 | 0.698 | 0.647 | 0.668 | 0.641 |
| CLASS | 0.974 | 0.990 | 0.997 | 0.995 | 0.991 |
| Cufflinks | 0.953 | 0.986 | 0.980 | 0.988 | 0.997 |
| FlipFlop | 0.807 | 0.760 | 0.671 | 0.605 | 0.623 |
| IsoLasso | 0.986 | 0.983 | 0.985 | 0.987 | 0.984 |
| MiTie | 0.332 | 0.424 | 0.427 | 0.457 | 0.510 |
| StringTie | 0.991 | 0.997 | 0.994 | 0.997 | 0.991 |
| Trinity | 0.832 | 0.907 | 0.824 | 0.838 | 0.894 |

Table S8 - Values to generate figure S2

| **DataSet T1** |  |  |  |  |  |
| --- | --- | --- | --- | --- | --- |
| **Figure S2** | **With Gene Models (TopHat2)** | | |  |  |
|  |  |  |  |  |  |
| **Left** | **Different exon starts/ends** | |  |  |  |
|  |  |  |  |  |  |
| Recall | One Splice Form | Two Splice Forms | Three Splice Forms | Four Splice Forms | Five Splice Forms |
| CLASS | 0.942 | 0.307 | 0.046 | 0.019 | 0.004 |
| Cufflinks | 0.934 | 0.775 | 0.837 | 0.753 | 0.796 |
| FlipFlop | 0.969 | 0.599 | 0.396 | 0.312 | 0.256 |
| IReckon | 0.499 | 0.740 | 0.823 | 0.749 | 0.797 |
| IsoLasso | 0.500 | 0.444 | 0.125 | 0.056 | 0.025 |
| MiTie | 0.918 | 0.766 | 0.841 | 0.757 | 0.800 |
| StringTie | 0.946 | 0.822 | 0.875 | 0.792 | 0.818 |
| StringTie-SR | 0.944 | 0.840 | 0.879 | 0.799 | 0.820 |
|  |  |  |  |  |  |
| Precision | One Splice Form | Two Splice Forms | Three Splice Forms | Four Splice Forms | Five Splice Forms |
| CLASS | 0.849 | 0.163 | 0.026 | 0.017 | 0.006 |
| Cufflinks | 0.944 | 0.748 | 0.863 | 0.854 | 0.942 |
| FlipFlop | 0.853 | 0.379 | 0.182 | 0.141 | 0.116 |
| IReckon | 1.000 | 0.427 | 0.613 | 0.577 | 0.644 |
| IsoLasso | 1.000 | 0.261 | 0.100 | 0.061 | 0.033 |
| MiTie | 0.925 | 0.398 | 0.417 | 0.388 | 0.402 |
| StringTie | 0.858 | 0.733 | 0.745 | 0.685 | 0.698 |
| StringTie-SR | 0.867 | 0.684 | 0.660 | 0.611 | 0.642 |
|  |  |  |  |  |  |
| **Middle** | **Skipped Exons** |  |  |  |  |
|  |  |  |  |  |  |
| Recall | One Splice Form | Two Splice Forms | Three Splice Forms | Four Splice Forms | Five Splice Forms |
| CLASS | 0.942 | 0.962 | 0.874 | 0.794 | 0.767 |
| Cufflinks | 0.934 | 0.873 | 0.861 | 0.779 | 0.804 |
| FlipFlop | 0.969 | 0.889 | 0.767 | 0.652 | 0.588 |
| IReckon | 0.499 | 0.899 | 0.893 | 0.838 | 0.872 |
| IsoLasso | 0.500 | 0.565 | 0.485 | 0.407 | 0.360 |
| MiTie | 0.918 | 0.844 | 0.871 | 0.766 | 0.807 |
| StringTie | 0.946 | 0.976 | 0.960 | 0.921 | 0.910 |
| StringTie-SR | 0.944 | 0.978 | 0.970 | 0.925 | 0.923 |
|  |  |  |  |  |  |
| Precision | One Splice Form | Two Splice Forms | Three Splice Forms | Four Splice Forms | Five Splice Forms |
| CLASS | 0.849 | 0.640 | 0.477 | 0.400 | 0.409 |
| Cufflinks | 0.944 | 0.786 | 0.848 | 0.816 | 0.899 |
| FlipFlop | 0.853 | 0.512 | 0.455 | 0.418 | 0.420 |
| IReckon | 1.000 | 0.775 | 0.768 | 0.739 | 0.794 |
| IsoLasso | 1.000 | 0.400 | 0.397 | 0.419 | 0.453 |
| MiTie | 0.925 | 0.438 | 0.446 | 0.409 | 0.442 |
| StringTie | 0.858 | 0.663 | 0.618 | 0.554 | 0.557 |
| StringTie-SR | 0.867 | 0.717 | 0.670 | 0.595 | 0.600 |
|  |  |  |  |  |  |
| **Right** | **Truncation** |  |  |  |  |
|  |  |  |  |  |  |
| Recall | One Splice Form | Two Splice Forms | Three Splice Forms | Four Splice Forms | Five Splice Forms |
| CLASS | 0.942 | 0.491 | 0.324 | 0.245 | 0.198 |
| Cufflinks | 0.934 | 0.715 | 0.641 | 0.529 | 0.499 |
| FlipFlop | 0.969 | 0.502 | 0.342 | 0.264 | 0.219 |
| IReckon | 0.499 | 0.460 | 0.396 | 0.375 | 0.377 |
| IsoLasso | 0.500 | 0.402 | 0.290 | 0.212 | 0.183 |
| MiTie | 0.918 | 0.809 | 0.858 | 0.770 | 0.809 |
| StringTie | 0.946 | 0.857 | 0.860 | 0.791 | 0.813 |
| StringTie-SR | 0.944 | 0.849 | 0.857 | 0.789 | 0.808 |
|  |  |  |  |  |  |
| Precision | One Splice Form | Two Splice Forms | Three Splice Forms | Four Splice Forms | Five Splice Forms |
| CLASS | 0.849 | 0.979 | 0.974 | 0.983 | 0.990 |
| Cufflinks | 0.944 | 0.962 | 0.952 | 0.927 | 0.926 |
| FlipFlop | 0.853 | 0.796 | 0.723 | 0.695 | 0.707 |
| IReckon | 1.000 | 0.916 | 0.910 | 0.854 | 0.866 |
| IsoLasso | 1.000 | 0.924 | 0.927 | 0.900 | 0.941 |
| MiTie | 0.925 | 0.721 | 0.726 | 0.627 | 0.625 |
| StringTie | 0.858 | 0.465 | 0.484 | 0.460 | 0.502 |
| StringTie-SR | 0.867 | 0.469 | 0.486 | 0.463 | 0.503 |

Table S9 - Values used to generate figure S3

| **DataSet T1** |  |  |  |  |  |
| --- | --- | --- | --- | --- | --- |
| **Figure S3** | **Without Gene Models (TopHat2)** | | |  |  |
|  |  |  |  |  |  |
| **Left** | **Different exon starts/ends** | |  |  |  |
|  |  |  |  |  |  |
| Recall | One Splice Form | Two Splice Forms | Three Splice Forms | Four Splice Forms | Five Splice Forms |
| AUGUSTUS | 0.403 | 0.169 | 0.143 | 0.101 | 0.091 |
| CLASS | 0.889 | 0.000 | 0.000 | 0.055 | 0.149 |
| Cufflinks | 0.878 | 0.385 | 0.262 | 0.200 | 0.159 |
| FlipFlop | 0.873 | 0.404 | 0.292 | 0.216 | 0.176 |
| IsoLasso | 0.834 | 0.328 | 0.213 | 0.137 | 0.093 |
| MiTie | 0.846 | 0.331 | 0.226 | 0.154 | 0.121 |
| StringTie | 0.891 | 0.417 | 0.282 | 0.213 | 0.171 |
| StringTie-SR | 0.905 | 0.428 | 0.296 | 0.224 | 0.178 |
|  |  |  |  |  |  |
| Precision | One Splice Form | Two Splice Forms | Three Splice Forms | Four Splice Forms | Five Splice Forms |
| AUGUSTUS | 0.665 | 0.499 | 0.509 | 0.484 | 0.498 |
| CLASS | 0.760 | 0.000 | 0.000 | 0.194 | 0.278 |
| Cufflinks | 0.772 | 0.530 | 0.429 | 0.372 | 0.388 |
| FlipFlop | 0.588 | 0.433 | 0.348 | 0.270 | 0.304 |
| IsoLasso | 0.712 | 0.502 | 0.364 | 0.238 | 0.211 |
| MiTie | 0.871 | 0.616 | 0.499 | 0.426 | 0.384 |
| StringTie | 0.833 | 0.593 | 0.491 | 0.442 | 0.404 |
| StringTie-SR | 0.771 | 0.520 | 0.419 | 0.342 | 0.307 |
|  |  |  |  |  |  |
| **Middle** | **Skipped Exons** |  |  |  |  |
|  |  |  |  |  |  |
| Recall | One Splice Form | Two Splice Forms | Three Splice Forms | Four Splice Forms | Five Splice Forms |
| AUGUSTUS | 0.403 | 0.209 | 0.144 | 0.110 | 0.096 |
| CLASS | 0.889 | 0.887 | 0.796 | 0.740 | 0.293 |
| Cufflinks | 0.878 | 0.689 | 0.515 | 0.437 | 0.391 |
| FlipFlop | 0.873 | 0.807 | 0.634 | 0.541 | 0.492 |
| IsoLasso | 0.834 | 0.607 | 0.433 | 0.345 | 0.310 |
| MiTie | 0.846 | 0.649 | 0.310 | 0.224 | 0.169 |
| StringTie | 0.891 | 0.807 | 0.639 | 0.566 | 0.534 |
| StringTie-SR | 0.905 | 0.748 | 0.623 | 0.551 | 0.535 |
|  |  |  |  |  |  |
| Precision | One Splice Form | Two Splice Forms | Three Splice Forms | Four Splice Forms | Five Splice Forms |
| AUGUSTUS | 0.665 | 0.357 | 0.279 | 0.300 | 0.309 |
| CLASS | 0.760 | 0.596 | 0.443 | 0.380 | 0.197 |
| Cufflinks | 0.772 | 0.590 | 0.482 | 0.456 | 0.498 |
| FlipFlop | 0.588 | 0.472 | 0.386 | 0.360 | 0.381 |
| IsoLasso | 0.712 | 0.426 | 0.345 | 0.315 | 0.343 |
| MiTie | 0.871 | 0.776 | 0.576 | 0.545 | 0.505 |
| StringTie | 0.833 | 0.684 | 0.508 | 0.447 | 0.417 |
| StringTie-SR | 0.771 | 0.581 | 0.470 | 0.408 | 0.402 |
|  |  |  |  |  |  |
| **Right** | **Truncation** |  |  |  |  |
|  |  |  |  |  |  |
| Recall | One Splice Form | Two Splice Forms | Three Splice Forms | Four Splice Forms | Five Splice Forms |
| AUGUSTUS | 0.403 | 0.214 | 0.127 | 0.107 | 0.089 |
| CLASS | 0.889 | 0.450 | 0.290 | 0.226 | 0.182 |
| Cufflinks | 0.878 | 0.426 | 0.279 | 0.208 | 0.169 |
| FlipFlop | 0.873 | 0.443 | 0.292 | 0.223 | 0.190 |
| IsoLasso | 0.834 | 0.402 | 0.264 | 0.198 | 0.165 |
| MiTie | 0.846 | 0.415 | 0.272 | 0.205 | 0.173 |
| StringTie | 0.891 | 0.450 | 0.291 | 0.227 | 0.183 |
| StringTie-SR | 0.905 | 0.461 | 0.300 | 0.232 | 0.188 |
|  |  |  |  |  |  |
| Precision | One Splice Form | Two Splice Forms | Three Splice Forms | Four Splice Forms | Five Splice Forms |
| AUGUSTUS | 0.665 | 0.667 | 0.619 | 0.641 | 0.613 |
| CLASS | 0.760 | 0.844 | 0.822 | 0.844 | 0.866 |
| Cufflinks | 0.772 | 0.819 | 0.800 | 0.777 | 0.797 |
| FlipFlop | 0.588 | 0.620 | 0.540 | 0.528 | 0.562 |
| IsoLasso | 0.712 | 0.783 | 0.782 | 0.794 | 0.819 |
| MiTie | 0.871 | 0.853 | 0.841 | 0.821 | 0.839 |
| StringTie | 0.833 | 0.840 | 0.793 | 0.786 | 0.808 |
| StringTie-SR | 0.771 | 0.799 | 0.810 | 0.777 | 0.810 |

Table S10 - Values used to generate figure S4

| **DataSet T1** |  |  |  |  |  |
| --- | --- | --- | --- | --- | --- |
| **Figure S4** | **With Gene Models (STAR)** | | |  |  |
|  |  |  |  |  |  |
| **Left** | **Different exon starts/ends** | |  |  |  |
|  |  |  |  |  |  |
| Recall | One Splice Form | Two Splice Forms | Three Splice Forms | Four Splice Forms | Five Splice Forms |
| CLASS | 0.955 | 0.437 | 0.259 | 0.166 | 0.107 |
| Cufflinks | 0.956 | 0.530 | 0.432 | 0.325 | 0.276 |
| FlipFlop | 0.938 | 0.371 | 0.166 | 0.107 | 0.068 |
| IReckon | 0.497 | 0.583 | 0.343 | 0.254 | 0.214 |
| IsoLasso | 0.485 | 0.392 | 0.206 | 0.122 | 0.074 |
| MiTie | 0.944 | 0.651 | 0.681 | 0.615 | 0.667 |
| StringTie | 0.963 | 0.475 | 0.317 | 0.241 | 0.193 |
| StringTie-SR | 0.956 | 0.475 | 0.316 | 0.239 | 0.193 |
|  |  |  |  |  |  |
| Precision | One Splice Form | Two Splice Forms | Three Splice Forms | Four Splice Forms | Five Splice Forms |
| CLASS | 0.959 | 0.640 | 0.437 | 0.348 | 0.284 |
| Cufflinks | 0.888 | 0.617 | 0.625 | 0.586 | 0.676 |
| FlipFlop | 0.690 | 0.192 | 0.082 | 0.058 | 0.042 |
| IReckon | 0.998 | 0.395 | 0.198 | 0.119 | 0.093 |
| IsoLasso | 0.898 | 0.443 | 0.305 | 0.255 | 0.191 |
| MiTie | 0.679 | 0.382 | 0.387 | 0.364 | 0.398 |
| StringTie | 0.922 | 0.637 | 0.571 | 0.532 | 0.505 |
| StringTie-SR | 0.879 | 0.479 | 0.361 | 0.289 | 0.263 |
|  |  |  |  |  |  |
| **Middle** | **Skipped Exons** |  |  |  |  |
|  |  |  |  |  |  |
| Recall | One Splice Form | Two Splice Forms | Three Splice Forms | Four Splice Forms | Five Splice Forms |
| CLASS | 0.955 | 0.900 | 0.794 | 0.742 | 0.710 |
| Cufflinks | 0.956 | 0.851 | 0.805 | 0.741 | 0.753 |
| FlipFlop | 0.938 | 0.723 | 0.555 | 0.449 | 0.385 |
| IReckon | 0.497 | 0.895 | 0.889 | 0.840 | 0.868 |
| IsoLasso | 0.485 | 0.651 | 0.531 | 0.438 | 0.385 |
| MiTie | 0.944 | 0.750 | 0.660 | 0.537 | 0.524 |
| StringTie | 0.963 | 0.954 | 0.931 | 0.884 | 0.891 |
| StringTie-SR | 0.956 | 0.952 | 0.943 | 0.900 | 0.899 |
|  |  |  |  |  |  |
| Precision | One Splice Form | Two Splice Forms | Three Splice Forms | Four Splice Forms | Five Splice Forms |
| CLASS | 0.959 | 0.465 | 0.368 | 0.328 | 0.320 |
| Cufflinks | 0.888 | 0.769 | 0.816 | 0.807 | 0.882 |
| FlipFlop | 0.690 | 0.350 | 0.298 | 0.251 | 0.233 |
| IReckon | 0.998 | 0.756 | 0.740 | 0.717 | 0.765 |
| IsoLasso | 0.898 | 0.522 | 0.482 | 0.477 | 0.490 |
| MiTie | 0.679 | 0.534 | 0.547 | 0.505 | 0.544 |
| StringTie | 0.922 | 0.778 | 0.755 | 0.686 | 0.684 |
| StringTie-SR | 0.879 | 0.728 | 0.689 | 0.630 | 0.622 |
|  |  |  |  |  |  |
| **Right** | **Truncation** |  |  |  |  |
|  |  |  |  |  |  |
| Recall | One Splice Form | Two Splice Forms | Three Splice Forms | Four Splice Forms | Five Splice Forms |
| CLASS | 0.955 | 0.475 | 0.312 | 0.239 | 0.192 |
| Cufflinks | 0.956 | 0.671 | 0.607 | 0.478 | 0.462 |
| FlipFlop | 0.938 | 0.481 | 0.311 | 0.245 | 0.196 |
| IReckon | 0.497 | 0.462 | 0.410 | 0.375 | 0.379 |
| IsoLasso | 0.485 | 0.392 | 0.278 | 0.206 | 0.179 |
| MiTie | 0.944 | 0.665 | 0.607 | 0.495 | 0.470 |
| StringTie | 0.963 | 0.848 | 0.841 | 0.780 | 0.797 |
| StringTie-SR | 0.956 | 0.828 | 0.818 | 0.756 | 0.775 |
|  |  |  |  |  |  |
| Precision | One Splice Form | Two Splice Forms | Three Splice Forms | Four Splice Forms | Five Splice Forms |
| CLASS | 0.959 | 0.955 | 0.944 | 0.957 | 0.948 |
| Cufflinks | 0.888 | 0.831 | 0.845 | 0.798 | 0.841 |
| FlipFlop | 0.690 | 0.570 | 0.481 | 0.419 | 0.378 |
| IReckon | 0.998 | 0.915 | 0.913 | 0.846 | 0.856 |
| IsoLasso | 0.898 | 0.802 | 0.801 | 0.744 | 0.742 |
| MiTie | 0.679 | 0.631 | 0.663 | 0.619 | 0.634 |
| StringTie | 0.922 | 0.748 | 0.729 | 0.641 | 0.633 |
| StringTie-SR | 0.879 | 0.458 | 0.472 | 0.453 | 0.496 |

Table S11 - Values to generate figure S5

| **DataSet T1** |  |  |  |  |  |
| --- | --- | --- | --- | --- | --- |
| **Figure S5** | **Without Gene Models (STAR)** | | |  |  |
|  |  |  |  |  |  |
| **Left** | **Different exon starts/ends** | |  |  |  |
|  |  |  |  |  |  |
| Recall | One Splice Form | Two Splice Forms | Three Splice Forms | Four Splice Forms | Five Splice Forms |
| AUGUSTUS | 0.420 | 0.181 | 0.149 | 0.107 | 0.094 |
| CLASS | 0.955 | 0.438 | 0.259 | 0.166 | 0.107 |
| Cufflinks | 0.806 | 0.325 | 0.218 | 0.153 | 0.118 |
| FlipFlop | na | na | na | na | na |
| IsoLasso | 0.916 | 0.362 | 0.217 | 0.147 | 0.096 |
| MiTie | 0.890 | 0.348 | 0.209 | 0.146 | 0.099 |
| StringTie | 0.959 | 0.423 | 0.286 | 0.224 | 0.178 |
| StringTie-SR | 0.954 | 0.451 | 0.290 | 0.225 | 0.178 |
|  |  |  |  |  |  |
| Precision | One Splice Form | Two Splice Forms | Three Splice Forms | Four Splice Forms | Five Splice Forms |
| AUGUSTUS | 0.679 | 0.490 | 0.481 | 0.449 | 0.467 |
| CLASS | 0.902 | 0.561 | 0.336 | 0.229 | 0.162 |
| Cufflinks | 0.765 | 0.534 | 0.460 | 0.452 | 0.442 |
| FlipFlop | na | na | na | na | na |
| IsoLasso | 0.877 | 0.495 | 0.310 | 0.241 | 0.186 |
| MiTie | 0.222 | 0.287 | 0.262 | 0.160 | 0.104 |
| StringTie | 0.919 | 0.574 | 0.512 | 0.494 | 0.467 |
| StringTie-SR | 0.872 | 0.457 | 0.325 | 0.271 | 0.242 |
|  |  |  |  |  |  |
| **Middle** | **Skipped Exons** |  |  |  |  |
|  |  |  |  |  |  |
| Recall | One Splice Form | Two Splice Forms | Three Splice Forms | Four Splice Forms | Five Splice Forms |
| AUGUSTUS | 0.420 | 0.218 | 0.153 | 0.116 | 0.100 |
| CLASS | 0.955 | 0.903 | 0.806 | 0.758 | 0.732 |
| Cufflinks | 0.806 | 0.471 | 0.243 | 0.154 | 0.110 |
| FlipFlop | na | na | na | na | na |
| IsoLasso | 0.916 | 0.644 | 0.466 | 0.373 | 0.339 |
| MiTie | 0.890 | 0.745 | 0.382 | 0.283 | 0.214 |
| StringTie | 0.959 | 0.780 | 0.607 | 0.529 | 0.475 |
| StringTie-SR | 0.954 | 0.758 | 0.625 | 0.558 | 0.526 |
|  |  |  |  |  |  |
| Precision | One Splice Form | Two Splice Forms | Three Splice Forms | Four Splice Forms | Five Splice Forms |
| AUGUSTUS | 0.679 | 0.366 | 0.288 | 0.311 | 0.319 |
| CLASS | 0.902 | 0.448 | 0.355 | 0.321 | 0.321 |
| Cufflinks | 0.765 | 0.624 | 0.524 | 0.489 | 0.543 |
| FlipFlop | na | na | na | na | na |
| IsoLasso | 0.877 | 0.454 | 0.378 | 0.346 | 0.362 |
| MiTie | 0.222 | 0.290 | 0.227 | 0.219 | 0.217 |
| StringTie | 0.919 | 0.648 | 0.494 | 0.422 | 0.394 |
| StringTie-SR | 0.872 | 0.584 | 0.468 | 0.399 | 0.385 |
|  |  |  |  |  |  |
| **Right** | **Truncation** |  |  |  |  |
|  |  |  |  |  |  |
| Recall | One Splice Form | Two Splice Forms | Three Splice Forms | Four Splice Forms | Five Splice Forms |
| AUGUSTUS | 0.420 | 0.221 | 0.133 | 0.112 | 0.091 |
| CLASS | 0.955 | 0.475 | 0.312 | 0.239 | 0.192 |
| Cufflinks | 0.806 | 0.401 | 0.255 | 0.192 | 0.162 |
| FlipFlop | na | na | na | na | na |
| IsoLasso | 0.916 | 0.440 | 0.291 | 0.219 | 0.177 |
| MiTie | 0.890 | 0.449 | 0.303 | 0.225 | 0.188 |
| StringTie | 0.959 | 0.484 | 0.315 | 0.241 | 0.194 |
| StringTie-SR | 0.954 | 0.476 | 0.312 | 0.238 | 0.191 |
|  |  |  |  |  |  |
| Precision | One Splice Form | Two Splice Forms | Three Splice Forms | Four Splice Forms | Five Splice Forms |
| AUGUSTUS | 0.679 | 0.672 | 0.627 | 0.653 | 0.626 |
| CLASS | 0.902 | 0.928 | 0.911 | 0.935 | 0.932 |
| Cufflinks | 0.765 | 0.790 | 0.774 | 0.778 | 0.799 |
| FlipFlop | na | na | na | na | na |
| IsoLasso | 0.877 | 0.883 | 0.873 | 0.877 | 0.880 |
| MiTie | 0.222 | 0.254 | 0.246 | 0.252 | 0.298 |
| StringTie | 0.919 | 0.932 | 0.892 | 0.900 | 0.911 |
| StringTie-SR | 0.872 | 0.896 | 0.845 | 0.878 | 0.891 |

Table S12 - Values to generate figure S6

| **DataSet T1** |  | |  | |  | |  | |  | |
| --- | --- | --- | --- | --- | --- | --- | --- | --- | --- | --- |
| **Figure S6** | | **De novo** | |  | |  | |  | |  |
|  | |  | |  | |  | |  | |  |
| **Left** | | **Different exon starts/ends** | | | |  | |  | |  |
|  | |  | |  | |  | |  | |  |
| Recall | | One Splice Form | | Two Splice Forms | | Three Splice Forms | | Four Splice Forms | | Five Splice Forms |
| SOAP | | 0.898 | | 0.055 | | 0.011 | | 0.004 | | 0.001 |
| Trans-ABySS | | 0.938 | | 0.153 | | 0.087 | | 0.095 | | 0.101 |
| Trinity | | 0.854 | | 0.317 | | 0.215 | | 0.147 | | 0.114 |
|  | |  | |  | |  | |  | |  |
| Precision | | One Splice Form | | Two Splice Forms | | Three Splice Forms | | Four Splice Forms | | Five Splice Forms |
| SOAP | | 0.802 | | 0.080 | | 0.017 | | 0.007 | | 0.002 |
| Trans-ABySS | | 0.751 | | 0.048 | | 0.023 | | 0.021 | | 0.026 |
| Trinity | | 0.758 | | 0.227 | | 0.119 | | 0.073 | | 0.062 |
|  | |  | |  | |  | |  | |  |
| **Middle** | | **Skipped Exons** | |  | |  | |  | |  |
|  | |  | |  | |  | |  | |  |
| Recall | | One Splice Form | | Two Splice Forms | | Three Splice Forms | | Four Splice Forms | | Five Splice Forms |
| SOAP | | 0.898 | | 0.377 | | 0.174 | | 0.099 | | 0.081 |
| Trans-ABySS | | 0.938 | | 0.517 | | 0.341 | | 0.297 | | 0.277 |
| Trinity | | 0.854 | | 0.713 | | 0.637 | | 0.593 | | 0.541 |
|  | |  | |  | |  | |  | |  |
| Precision | | One Splice Form | | Two Splice Forms | | Three Splice Forms | | Four Splice Forms | | Five Splice Forms |
| SOAP | | 0.802 | | 0.411 | | 0.232 | | 0.156 | | 0.141 |
| Trans-ABySS | | 0.751 | | 0.264 | | 0.152 | | 0.129 | | 0.129 |
| Trinity | | 0.758 | | 0.395 | | 0.337 | | 0.311 | | 0.303 |
|  | |  | |  | |  | |  | |  |
| **Right** | | **Truncation** | |  | |  | |  | |  |
|  | |  | |  | |  | |  | |  |
| Recall | | One Splice Form | | Two Splice Forms | | Three Splice Forms | | Four Splice Forms | | Five Splice Forms |
| SOAP | | 0.898 | | 0.442 | | 0.291 | | 0.214 | | 0.182 |
| Trans-ABySS | | 0.938 | | 0.463 | | 0.302 | | 0.226 | | 0.188 |
| Trinity | | 0.854 | | 0.414 | | 0.270 | | 0.204 | | 0.170 |
|  | |  | |  | |  | |  | |  |
| Precision | | One Splice Form | | Two Splice Forms | | Three Splice Forms | | Four Splice Forms | | Five Splice Forms |
| SOAP | | 0.802 | | 0.805 | | 0.853 | | 0.775 | | 0.827 |
| Trans-ABySS | | 0.751 | | 0.856 | | 0.830 | | 0.760 | | 0.839 |
| Trinity | | 0.758 | | 0.782 | | 0.753 | | 0.733 | | 0.812 |

Table S13 - Values used to generate figure S7

| **DataSet EP and ER** | | | |  |
| --- | --- | --- | --- | --- |
| **Figure S7** | **Without Gene Models (Truth)** | | | |
|  |  |  |  | |
| **Left** | **EnsemblPerfect** |  |  | |
|  |  |  |  | |
| Recall | 0-10X | 10-100X | 100X_and_more | |
| AUGUSTUS | 0.046 | 0.120 | 0.215 | |
| CLASS | 0.169 | 0.379 | 0.515 | |
| Cufflinks | 0.152 | 0.419 | 0.681 | |
| FlipFlop | 0.131 | 0.404 | 0.637 | |
| IsoLasso | 0.046 | 0.385 | 0.574 | |
| MiTie | 0.039 | 0.146 | 0.446 | |
| StringTie | 0.191 | 0.448 | 0.680 | |
| Trinity | 0.194 | 0.508 | 0.702 | |
|  |  |  |  | |
| Precision | 0-10X | 10-100X | 100X_and_more | |
| AUGUSTUS | 0.233 | 0.356 | 0.391 | |
| CLASS | 0.507 | 0.602 | 0.563 | |
| Cufflinks | 0.635 | 0.759 | 0.753 | |
| FlipFlop | 0.378 | 0.547 | 0.537 | |
| IsoLasso | 0.239 | 0.636 | 0.617 | |
| MiTie | 0.367 | 0.560 | 0.704 | |
| StringTie | 0.635 | 0.721 | 0.710 | |
| Trinity | 0.218 | 0.331 | 0.304 | |
|  |  |  |  | |
| **Right** | **EnsemblRealistic** |  |  | |
|  |  |  |  | |
| Recall | 0-10X | 10-100X | 100X_and_more | |
| AUGUSTUS | 0.039 | 0.135 | 0.253 | |
| CLASS | 0.161 | 0.381 | 0.536 | |
| Cufflinks | 0.129 | 0.445 | 0.738 | |
| FlipFlop | 0.093 | 0.378 | 0.628 | |
| IsoLasso | 0.034 | 0.334 | 0.574 | |
| MiTie | 0.006 | 0.048 | 0.302 | |
| StringTie | 0.151 | 0.470 | 0.754 | |
| Trinity | 0.183 | 0.535 | 0.754 | |
|  |  |  |  | |
| Precision | 0-10X | 10-100X | 100X_and_more | |
| AUGUSTUS | 0.277 | 0.359 | 0.368 | |
| CLASS | 0.583 | 0.564 | 0.510 | |
| Cufflinks | 0.525 | 0.624 | 0.617 | |
| FlipFlop | 0.327 | 0.452 | 0.425 | |
| IsoLasso | 0.267 | 0.592 | 0.591 | |
| MiTie | 0.098 | 0.252 | 0.512 | |
| StringTie | 0.520 | 0.578 | 0.560 | |
| Trinity | 0.165 | 0.191 | 0.150 | |

Table S14 - Values used to generate figure S8

| **DataSet EP and ER** | |  |  |
| --- | --- | --- | --- |
| **Figure S8** | **With Gene Models (TopHat2)** | |  |
|  |  |  |  |
| **Left** | **EnsemblPerfect** |  |  |
|  |  |  |  |
| Recall | 0-10X | 10-100X | 100X_and_more |
| CLASS | 0.153 | 0.365 | 0.502 |
| Cufflinks | 0.297 | 0.576 | 0.867 |
| FlipFlop | 0.190 | 0.436 | 0.579 |
| IReckon | 0.576 | 0.699 | 0.767 |
| IsoLasso | 0.073 | 0.398 | 0.553 |
| MiTie | 0.599 | 0.714 | 0.794 |
| StringTie | 0.792 | 0.896 | 0.931 |
| Stringtie-SR | 0.771 | 0.894 | 0.932 |
|  |  |  |  |
| Precision | 0-10X | 10-100X | 100X_and_more |
| CLASS | 0.473 | 0.589 | 0.555 |
| Cufflinks | 0.921 | 0.945 | 0.941 |
| FlipFlop | 0.467 | 0.560 | 0.511 |
| IReckon | 0.813 | 0.778 | 0.702 |
| IsoLasso | 0.343 | 0.641 | 0.609 |
| MiTie | 0.856 | 0.824 | 0.766 |
| StringTie | 0.891 | 0.859 | 0.794 |
| Stringtie-SR | 0.889 | 0.857 | 0.788 |
|  |  |  |  |
| **Right** | **EnsemblRealistic** |  |  |
|  |  |  |  |
| Recall | 0-10X | 10-100X | 100X_and_more |
| CLASS | 0.157 | 0.380 | 0.551 |
| Cufflinks | 0.275 | 0.626 | 0.896 |
| FlipFlop | 0.093 | 0.238 | 0.250 |
| IReckon | 0.515 | 0.684 | 0.770 |
| IsoLasso | 0.043 | 0.279 | 0.488 |
| MiTie | 0.578 | 0.708 | 0.773 |
| StringTie | 0.746 | 0.885 | 0.932 |
| Stringtie-SR | 0.719 | 0.880 | 0.930 |
|  |  |  |  |
| Precision | 0-10X | 10-100X | 100X_and_more |
| CLASS | 0.578 | 0.568 | 0.524 |
| Cufflinks | 0.805 | 0.811 | 0.780 |
| FlipFlop | 0.179 | 0.186 | 0.111 |
| IReckon | 0.727 | 0.592 | 0.480 |
| IsoLasso | 0.277 | 0.499 | 0.482 |
| MiTie | 0.871 | 0.765 | 0.661 |
| StringTie | 0.873 | 0.771 | 0.678 |
| Stringtie-SR | 0.866 | 0.758 | 0.664 |

Table S15 - Values used to generate figure S9

| **DataSet EP and ER** | |  |  |
| --- | --- | --- | --- |
| **Figure S9** | **Without Gene Models (TopHat2)** | |  |
|  |  |  |  |
| **Left** | **EnsemblPerfect** |  |  |
|  |  |  |  |
| Recall | 0-10X | 10-100X | 100X_and_more |
| AUGUSTUS | 0.044 | 0.112 | 0.202 |
| CLASS | 0.124 | 0.326 | 0.461 |
| Cufflinks | 0.111 | 0.342 | 0.568 |
| FlipFlop | 0.103 | 0.329 | 0.530 |
| IsoLasso | 0.043 | 0.300 | 0.454 |
| MiTie | 0.036 | 0.130 | 0.385 |
| StringTie | 0.145 | 0.377 | 0.594 |
| Stringtie-SR | 0.145 | 0.383 | 0.598 |
| Trinity | 0.177 | 0.439 | 0.630 |
|  |  |  |  |
| Precision | 0-10X | 10-100X | 100X_and_more |
| AUGUSTUS | 0.230 | 0.344 | 0.378 |
| CLASS | 0.402 | 0.542 | 0.512 |
| Cufflinks | 0.493 | 0.655 | 0.661 |
| FlipFlop | 0.290 | 0.468 | 0.469 |
| IsoLasso | 0.209 | 0.537 | 0.527 |
| MiTie | 0.311 | 0.496 | 0.642 |
| StringTie | 0.476 | 0.625 | 0.622 |
| Stringtie-SR | 0.496 | 0.631 | 0.617 |
| Trinity | 0.435 | 0.545 | 0.515 |
|  |  |  |  |
| **Right** | **EnsemblRealistic** |  |  |
|  |  |  |  |
| Recall | 0-10X | 10-100X | 100X_and_more |
| AUGUSTUS | 0.041 | 0.133 | 0.246 |
| CLASS | 0.121 | 0.332 | 0.496 |
| Cufflinks | 0.090 | 0.345 | 0.563 |
| FlipFlop | 0.063 | 0.289 | 0.497 |
| IsoLasso | 0.026 | 0.241 | 0.419 |
| StringTie | 0.110 | 0.379 | 0.611 |
| Stringtie-SR | 0.108 | 0.374 | 0.602 |
| Trinity | NA | NA | NA |
|  |  |  |  |
| Precision | 0-10X | 10-100X | 100X_and_more |
| AUGUSTUS | 0.292 | 0.357 | 0.365 |
| CLASS | 0.477 | 0.498 | 0.463 |
| Cufflinks | 0.383 | 0.501 | 0.478 |
| FlipFlop | 0.233 | 0.361 | 0.350 |
| IsoLasso | 0.200 | 0.468 | 0.461 |
| StringTie | 0.391 | 0.469 | 0.461 |
| Stringtie-SR | 0.377 | 0.454 | 0.434 |
| Trinity | NA | NA | NA |

Table S16 - Values used to generate figure S10

| **DataSet EP and ER** |  |  |  |
| --- | --- | --- | --- |
| **Figure S10** | **With Gene Models (STAR)** |  |  |
|  |  |  |  |
| **Left** | **EnsemblPerfect** |  |  |
|  |  |  |  |
| Recall | 0-10X | 10-100X | 100X_and_more |
| CLASS | 0.107 | 0.255 | 0.361 |
| Cufflinks | 0.282 | 0.557 | 0.860 |
| FlipFlop | 0.127 | 0.310 | 0.448 |
| IReckon | 0.571 | 0.693 | 0.765 |
| IsoLasso | 0.059 | 0.365 | 0.519 |
| MiTie | 0.578 | 0.701 | 0.793 |
| StringTie | 0.711 | 0.837 | 0.879 |
| Stringtie-SR | 0.681 | 0.828 | 0.871 |
|  |  |  |  |
| Precision | 0-10X | 10-100X | 100X_and_more |
| CLASS | 0.420 | 0.529 | 0.503 |
| Cufflinks | 0.898 | 0.925 | 0.929 |
| FlipFlop | 0.319 | 0.432 | 0.396 |
| IReckon | 0.802 | 0.764 | 0.689 |
| IsoLasso | 0.280 | 0.606 | 0.578 |
| MiTie | 0.788 | 0.744 | 0.668 |
| StringTie | 0.861 | 0.827 | 0.753 |
| Stringtie-SR | 0.852 | 0.827 | 0.753 |
|  |  |  |  |
| **Right** | **EnsemblRealistic** |  |  |
|  |  |  |  |
| Recall | 0-10X | 10-100X | 100X_and_more |
| CLASS | 0.114 | 0.295 | 0.418 |
| Cufflinks | 0.252 | 0.580 | 0.858 |
| FlipFlop | 0.075 | 0.174 | 0.189 |
| IReckon | 0.506 | 0.673 | 0.757 |
| IsoLasso | 0.035 | 0.232 | 0.414 |
| StringTie | 0.620 | 0.770 | 0.788 |
| Stringtie-SR | 0.591 | 0.759 | 0.777 |
|  |  |  |  |
| Precision | 0-10X | 10-100X | 100X_and_more |
| CLASS | 0.467 | 0.490 | 0.431 |
| Cufflinks | 0.767 | 0.776 | 0.743 |
| FlipFlop | 0.147 | 0.138 | 0.083 |
| IReckon | 0.707 | 0.563 | 0.445 |
| IsoLasso | 0.225 | 0.434 | 0.422 |
| StringTie | 0.809 | 0.678 | 0.548 |
| Stringtie-SR | 0.797 | 0.678 | 0.550 |

Table S17 - Values used to generate figure S11

| **DataSet EP and ER** |  |  |  |
| --- | --- | --- | --- |
| **Figure S11** | **Without Gene Models (STAR)** | |  |
|  |  |  |  |
| **Left** | **EnsemblPerfect** |  |  |
|  |  |  |  |
| Recall | 0-10X | 10-100X | 100X_and_more |
| AUGUSTUS | 0.043 | 0.114 | 0.207 |
| CLASS | 0.107 | 0.256 | 0.362 |
| Cufflinks | 0.125 | 0.365 | 0.613 |
| FlipFlop | 0.077 | 0.315 | 0.555 |
| IsoLasso | 0.046 | 0.334 | 0.527 |
| MiTie | 0.035 | 0.133 | 0.410 |
| StringTie | 0.147 | 0.388 | 0.635 |
| Stringtie-SR | 0.148 | 0.388 | 0.613 |
| Trinity | 0.205 | 0.480 | 0.683 |
|  |  |  |  |
| Precision | 0-10X | 10-100X | 100X_and_more |
| AUGUSTUS | 0.223 | 0.349 | 0.383 |
| CLASS | 0.420 | 0.528 | 0.502 |
| Cufflinks | 0.527 | 0.687 | 0.694 |
| FlipFlop | 0.236 | 0.432 | 0.434 |
| IsoLasso | 0.238 | 0.586 | 0.585 |
| MiTie | 0.204 | 0.369 | 0.520 |
| StringTie | 0.501 | 0.634 | 0.637 |
| Stringtie-SR | 0.511 | 0.645 | 0.644 |
| Trinity | 0.479 | 0.575 | 0.544 |
|  |  |  |  |
| **Right** | **EnsemblRealistic** |  |  |
|  |  |  |  |
| Recall | 0-10X | 10-100X | 100X_and_more |
| AUGUSTUS | 0.040 | 0.133 | 0.250 |
| CLASS | 0.122 | 0.297 | 0.418 |
| Cufflinks | 0.094 | 0.353 | 0.582 |
| IsoLasso | 0.030 | 0.251 | 0.425 |
| MiTie | 0.010 | 0.074 | 0.316 |
| StringTie | 0.109 | 0.377 | 0.615 |
| Stringtie-SR | 0.110 | 0.380 | 0.601 |
| Trinity | 0.184 | 0.491 | 0.703 |
|  |  |  |  |
| Precision | 0-10X | 10-100X | 100X_and_more |
| AUGUSTUS | 0.280 | 0.358 | 0.366 |
| CLASS | 0.480 | 0.489 | 0.427 |
| Cufflinks | 0.376 | 0.489 | 0.473 |
| IsoLasso | 0.222 | 0.487 | 0.470 |
| MiTie | 0.056 | 0.146 | 0.263 |
| StringTie | 0.375 | 0.451 | 0.437 |
| Stringtie-SR | 0.380 | 0.465 | 0.438 |
| Trinity | 0.260 | 0.279 | 0.218 |

Table S18 - Values used to generate figure S12

| **DataSet EP and ER** |  |  |  |
| --- | --- | --- | --- |
| **Figure S12** | **De novo** |  |  |
|  |  |  |  |
| **Left** | **EnsemblPerfect** |  |  |
|  |  |  |  |
| Recall | 0-10X | 10-100X | 100X_and_more |
| SOAP | 0.119 | 0.290 | 0.424 |
| Trans-ABySS | 0.252 | 0.406 | 0.545 |
| Trinity | 0.182 | 0.437 | 0.619 |
|  |  |  |  |
| Precision | 0-10X | 10-100X | 100X_and_more |
| SOAP | 0.379 | 0.493 | 0.468 |
| Trans-ABySS | 0.417 | 0.430 | 0.385 |
| Trinity | 0.451 | 0.563 | 0.529 |
|  |  |  |  |
| **Right** | **EnsemblRealistic** |  |  |
|  |  |  |  |
| Recall | 0-10X | 10-100X | 100X_and_more |
| SOAP | 0.079 | 0.288 | 0.516 |
| Trans-ABySS | 0.273 | 0.439 | 0.637 |
| Trinity | 0.171 | 0.446 | 0.647 |
|  |  |  |  |
| Precision | 0-10X | 10-100X | 100X_and_more |
| SOAP | 0.316 | 0.408 | 0.405 |
| Trans-ABySS | 0.504 | 0.404 | 0.360 |
| Trinity | 0.304 | 0.321 | 0.273 |

Table S19 - Values for generating figure S13

| **IVT** |  |  |  |  |  |  |  |  |  |
| --- | --- | --- | --- | --- | --- | --- | --- | --- | --- |
| **Figure S13** | **Without Gene Models** | | |  |  |  |  |  |  |
|  |  |  |  |  |  |  |  |  |  |
| **Left** | **PolyA** |  |  |  | **Right** | **Simulated** |  |  |  |
|  |  |  |  |  |  |  |  |  |  |
| Recall | none | STAR | Tophat2 |  | Recall | none | STAR | Tophat2 | Truth |
| AUGUSTUS | NA | 0.293 | 0.295 |  | AUGUSTUS | NA | 0.320 | 0.320 | 0.322 |
| CLASS | NA | 0.095 | 0.406 |  | CLASS | NA | 0.748 | 0.688 | 0.854 |
| Cufflinks | NA | 0.252 | 0.108 |  | Cufflinks | NA | 0.741 | 0.679 | 0.858 |
| FlipFlop | NA | NA | 0.056 |  | FlipFlop | NA | 0.731 | 0.655 | 0.833 |
| IsoLasso | NA | 0.100 | 0.050 |  | IsoLasso | NA | 0.766 | 0.683 | 0.876 |
| MiTie | NA | 0.205 | 0.314 |  | MiTie | NA | 0.749 | 0.636 | 0.848 |
| SOAP | 0.373 | NA | NA |  | SOAP | 0.728 | NA | NA | NA |
| StringTie | NA | 0.156 | 0.043 |  | StringTie | NA | 0.766 | 0.697 | 0.835 |
| StringTie-SR | NA | 0.158 | 0.045 |  | StringTie-SR | NA | 0.757 | 0.738 | NA |
| Trans-ABySS | 0.299 | NA | NA |  | Trans-ABySS | 0.770 | NA | NA | NA |
| Trinity | 0.394 | 0.289 | 0.474 |  | Trinity | 0.748 | 0.772 | 0.765 | 0.756 |
|  |  |  |  |  |  |  |  |  |  |
| Precision | none | STAR | Tophat2 |  | Precision | none | STAR | Tophat2 | Truth |
| AUGUSTUS | NA | 0.448 | 0.455 |  | AUGUSTUS | NA | 0.450 | 0.450 | 0.451 |
| CLASS | NA | 0.216 | 0.492 |  | CLASS | NA | 0.785 | 0.698 | 0.893 |
| Cufflinks | NA | 0.600 | 0.412 |  | Cufflinks | NA | 0.763 | 0.683 | 0.886 |
| FlipFlop | NA | NA | 0.076 |  | FlipFlop | NA | 0.313 | 0.400 | 0.558 |
| IsoLasso | NA | 0.162 | 0.136 |  | IsoLasso | NA | 0.785 | 0.662 | 0.906 |
| MiTie | NA | 0.214 | 0.227 |  | MiTie | NA | 0.431 | 0.345 | 0.728 |
| SOAP | 0.620 | NA | NA |  | SOAP | 0.786 | NA | NA | NA |
| StringTie | NA | 0.352 | 0.156 |  | StringTie | NA | 0.785 | 0.697 | 0.820 |
| StringTie-SR | NA | 0.361 | 0.162 |  | StringTie-SR | NA | 0.775 | 0.727 | NA |
| Trans-ABySS | 0.637 | NA | NA |  | Trans-ABySS | 0.809 | NA | NA | NA |
| Trinity | 0.700 | 0.532 | 0.741 |  | Trinity | 0.797 | 0.755 | 0.776 | 0.764 |
|  |  |  |  |  |  |  |  |  |  |
| **Middle** | **Ribo** |  |  |  |  |  |  |  |  |
|  |  |  |  |  |  |  |  |  |  |
| Recall | none | STAR | Tophat2 |  |  |  |  |  |  |
| AUGUSTUS | NA | 0.290 | 0.295 |  |  |  |  |  |  |
| CLASS | NA | 0.086 | 0.435 |  |  |  |  |  |  |
| Cufflinks | NA | 0.370 | 0.153 |  |  |  |  |  |  |
| FlipFlop | NA | NA | 0.073 |  |  |  |  |  |  |
| IsoLasso | NA | 0.133 | 0.066 |  |  |  |  |  |  |
| MiTie | NA | 0.308 | 0.411 |  |  |  |  |  |  |
| SOAP | 0.406 | NA | NA |  |  |  |  |  |  |
| StringTie | NA | 0.200 | 0.079 |  |  |  |  |  |  |
| StringTie-SR | NA | 0.201 | 0.080 |  |  |  |  |  |  |
| Trans-ABySS | 0.264 | NA | NA |  |  |  |  |  |  |
| Trinity | 0.417 | 0.307 | 0.474 |  |  |  |  |  |  |
|  |  |  |  |  |  |  |  |  |  |
| Precision | none | STAR | Tophat2 |  |  |  |  |  |  |
| AUGUSTUS | NA | 0.446 | 0.453 |  |  |  |  |  |  |
| CLASS | NA | 0.297 | 0.528 |  |  |  |  |  |  |
| Cufflinks | NA | 0.603 | 0.432 |  |  |  |  |  |  |
| FlipFlop | NA | NA | 0.078 |  |  |  |  |  |  |
| IsoLasso | NA | 0.237 | 0.208 |  |  |  |  |  |  |
| MiTie | NA | 0.220 | 0.227 |  |  |  |  |  |  |
| SOAP | 0.641 | NA | NA |  |  |  |  |  |  |
| StringTie | NA | 0.431 | 0.285 |  |  |  |  |  |  |
| StringTie-SR | NA | 0.435 | 0.290 |  |  |  |  |  |  |
| Trans-ABySS | 0.599 | NA | NA |  |  |  |  |  |  |
| Trinity | 0.703 | 0.514 | 0.724 |  |  |  |  |  |  |

Table S20 - Values to generate figure S14

| **DataSet T1** |  |  |  |  |  |
| --- | --- | --- | --- | --- | --- |
| **Figure S14** | **With Gene Models (Truth)** | |  |  |  |
|  |  |  |  |  |  |
| **Left** | **Different exon starts/ends** | |  |  |  |
|  |  |  |  |  |  |
| Recall | One Splice Form | Two Splice Forms | Three Splice Forms | Four Splice Forms | Five Splice Forms |
| CLASS | 0.988 | 0.203 | 0.012 | 0.002 | 0.000 |
| Cufflinks | 0.997 | 0.838 | 0.852 | 0.762 | 0.810 |
| FlipFlop | 1.000 | 0.637 | 0.410 | 0.339 | 0.269 |
| IReckon | 0.499 | 0.776 | 0.817 | 0.764 | 0.803 |
| IsoLasso | 0.500 | 0.468 | 0.135 | 0.059 | 0.026 |
| MiTie | 0.926 | 0.851 | 0.883 | 0.771 | 0.810 |
| StringTie | 0.990 | 0.532 | 0.366 | 0.289 | 0.263 |
|  |  |  |  |  |  |
| Precision | One Splice Form | Two Splice Forms | Three Splice Forms | Four Splice Forms | Five Splice Forms |
| CLASS | 0.909 | 0.098 | 0.008 | 0.002 | 0.001 |
| Cufflinks | 0.988 | 0.709 | 0.790 | 0.794 | 0.895 |
| FlipFlop | 0.840 | 0.345 | 0.171 | 0.128 | 0.109 |
| IReckon | 1.000 | 0.600 | 0.569 | 0.544 | 0.626 |
| IsoLasso | 1.000 | 0.337 | 0.123 | 0.069 | 0.037 |
| MiTie | 0.647 | 0.353 | 0.379 | 0.347 | 0.366 |
| StringTie | 0.991 | 0.506 | 0.362 | 0.314 | 0.329 |
|  |  |  |  |  |  |
| **Middle** | **Skipped Exons** |  |  |  |  |
|  |  |  |  |  |  |
| Recall | One Splice Form | Two Splice Forms | Three Splice Forms | Four Splice Forms | Five Splice Forms |
| CLASS | 0.988 | 0.979 | 0.906 | 0.834 | 0.793 |
| Cufflinks | 0.997 | 0.913 | 0.887 | 0.815 | 0.817 |
| FlipFlop | 1.000 | 0.905 | 0.778 | 0.669 | 0.598 |
| IReckon | 0.499 | 0.906 | 0.895 | 0.854 | 0.873 |
| IsoLasso | 0.500 | 0.679 | 0.555 | 0.447 | 0.392 |
| MiTie | 0.926 | 0.869 | 0.889 | 0.787 | 0.815 |
| StringTie | 0.990 | 0.986 | 0.981 | 0.945 | 0.931 |
|  |  |  |  |  |  |
| Precision | One Splice Form | Two Splice Forms | Three Splice Forms | Four Splice Forms | Five Splice Forms |
| CLASS | 0.909 | 0.671 | 0.505 | 0.435 | 0.429 |
| Cufflinks | 0.988 | 0.832 | 0.835 | 0.822 | 0.885 |
| FlipFlop | 0.840 | 0.538 | 0.477 | 0.434 | 0.428 |
| IReckon | 1.000 | 0.774 | 0.750 | 0.737 | 0.769 |
| IsoLasso | 1.000 | 0.547 | 0.505 | 0.493 | 0.511 |
| MiTie | 0.647 | 0.378 | 0.400 | 0.379 | 0.409 |
| StringTie | 0.991 | 0.818 | 0.781 | 0.731 | 0.718 |
|  |  |  |  |  |  |
| **Right** | **Truncation** |  |  |  |  |
|  |  |  |  |  |  |
| Recall | One Splice Form | Two Splice Forms | Three Splice Forms | Four Splice Forms | Five Splice Forms |
| CLASS | 0.988 | 0.494 | 0.330 | 0.249 | 0.198 |
| Cufflinks | 0.997 | 0.726 | 0.646 | 0.540 | 0.501 |
| FlipFlop | 1.000 | 0.504 | 0.343 | 0.265 | 0.218 |
| IReckon | 0.499 | 0.460 | 0.403 | 0.374 | 0.377 |
| IsoLasso | 0.500 | 0.403 | 0.292 | 0.212 | 0.183 |
| MiTie | 0.926 | 0.819 | 0.867 | 0.779 | 0.815 |
| StringTie | 0.990 | 0.869 | 0.879 | 0.803 | 0.817 |
|  |  |  |  |  |  |
| Precision | One Splice Form | Two Splice Forms | Three Splice Forms | Four Splice Forms | Five Splice Forms |
| CLASS | 0.909 | 0.980 | 0.983 | 0.993 | 0.979 |
| Cufflinks | 0.988 | 0.902 | 0.907 | 0.862 | 0.893 |
| FlipFlop | 0.840 | 0.794 | 0.716 | 0.666 | 0.687 |
| IReckon | 1.000 | 0.918 | 0.919 | 0.854 | 0.867 |
| IsoLasso | 1.000 | 0.925 | 0.932 | 0.901 | 0.941 |
| MiTie | 0.647 | 0.585 | 0.623 | 0.559 | 0.569 |
| StringTie | 0.991 | 0.774 | 0.750 | 0.656 | 0.642 |

Table S21 - Values to generate figure S15

| **DataSet T1** |  |  |  |  |  |  |  |  |  |  |
| --- | --- | --- | --- | --- | --- | --- | --- | --- | --- | --- |
| **Figure S15** | **With Gene Models (TopHat2) - Split by annotation** | | | | | |  |  |  | |
|  |  |  |  |  |  |  |  |  |  |  |
| **Left** | **Different exon starts/ends** | | | | | |  |  |  |  |
|  |  |  |  |  |  |  |  |  |  |  |
| Recall | One Splice Form | Two Splice Forms | Three Splice Forms | Four Splice Forms | Five Splice Forms | One Splice Form Annotated | Two Splice Forms Annotated | Three Splice Forms Annotated | Four Splice Forms Annotated | Five Splice Forms Annotated |
| CLASS | 0.890 | 0.080 | 0.008 | 0.009 | 0.001 | 1.000 | 0.383 | 0.054 | 1.000 | 0.005 |
| Cufflinks | 0.868 | 0.128 | 0.074 | 0.056 | 0.035 | 1.000 | 0.991 | 0.990 | 0.985 | 0.986 |
| FlipFlop | 0.938 | 0.242 | 0.142 | 0.148 | 0.103 | 1.000 | 0.718 | 0.447 | 0.367 | 0.294 |
| IReckon | 0.000 | 0.172 | 0.068 | 0.046 | 0.007 | 1.000 | 0.929 | 0.974 | 0.984 | 0.995 |
| IsoLasso | 0.000 | 0.128 | 0.050 | 0.021 | 0.004 | 1.000 | 0.549 | 0.140 | 0.068 | 0.030 |
| MiTie | 0.836 | 0.064 | 0.048 | 0.039 | 0.011 | 1.000 | 1.000 | 1.000 | 0.997 | 0.997 |
| StringTie | 0.892 | 0.288 | 0.264 | 0.179 | 0.089 | 1.000 | 1.000 | 0.997 | 0.996 | 1.000 |
| StringTie-SR | 0.888 | 0.360 | 0.288 | 0.207 | 0.099 | 1.000 | 0.999 | 0.998 | 0.996 | 1.000 |
|  |  |  |  |  |  |  |  |  |  |  |
| Precision | One Splice Form | Two Splice Forms | Three Splice Forms | Four Splice Forms | Five Splice Forms | One Splice Form Annotated | Two Splice Forms Annotated | Three Splice Forms Annotated | Four Splice Forms Annotated | Five Splice Forms Annotated |
| CLASS | 0.802 | 0.013 | 0.001 | 0.002 | 0.000 | 0.994 | 0.998 | 0.993 | 0.023 | 0.947 |
| Cufflinks | 0.816 | 0.122 | 0.094 | 0.114 | 0.148 | 1.000 | 0.959 | 0.984 | 0.972 | 0.989 |
| FlipFlop | 0.737 | 0.058 | 0.013 | 0.019 | 0.010 | 1.000 | 0.999 | 0.997 | 0.997 | 0.996 |
| IReckon | 0.000 | 0.050 | 0.053 | 0.062 | 0.029 | 0.998 | 0.790 | 0.720 | 0.663 | 0.669 |
| IsoLasso | NA | 0.025 | 0.007 | 0.006 | 0.001 | 1.000 | 0.983 | 0.989 | 0.949 | 0.952 |
| MiTie | 0.860 | 0.096 | 0.044 | 0.048 | 0.011 | 0.988 | 0.426 | 0.453 | 0.428 | 0.443 |
| StringTie | 0.748 | 0.220 | 0.145 | 0.128 | 0.063 | 0.988 | 0.943 | 0.954 | 0.926 | 0.901 |
| StringTie-SR | 0.755 | 0.200 | 0.101 | 0.100 | 0.048 | 0.998 | 0.963 | 0.968 | 0.944 | 0.925 |
|  |  |  |  |  |  |  |  |  |  |  |
| **Middle** | **Skipped Exons** |  |  |  |  |  |  |  |  |  |
|  |  |  |  |  |  |  |  |  |  |  |
| Recall | One Splice Form | Two Splice Forms | Three Splice Forms | Four Splice Forms | Five Splice Forms | One Splice Form Annotated | Two Splice Forms Annotated | Three Splice Forms Annotated | Four Splice Forms Annotated | Five Splice Forms Annotated |
| CLASS | 0.890 | 0.916 | 0.804 | 0.721 | 0.717 | 1.000 | 0.977 | 0.888 | 0.818 | 0.780 |
| Cufflinks | 0.868 | 0.572 | 0.368 | 0.300 | 0.291 | 1.000 | 0.973 | 0.960 | 0.938 | 0.932 |
| FlipFlop | 0.938 | 0.856 | 0.714 | 0.631 | 0.549 | 1.000 | 0.900 | 0.778 | 0.659 | 0.598 |
| IReckon | 0.000 | 0.726 | 0.602 | 0.473 | 0.521 | 1.000 | 0.956 | 0.951 | 0.960 | 0.959 |
| IsoLasso | 0.000 | 0.410 | 0.460 | 0.394 | 0.371 | 1.000 | 0.616 | 0.490 | 0.412 | 0.357 |
| MiTie | 0.836 | 0.380 | 0.228 | 0.062 | 0.035 | 1.000 | 0.999 | 1.000 | 1.000 | 1.000 |
| StringTie | 0.892 | 0.928 | 0.858 | 0.713 | 0.594 | 1.000 | 0.992 | 0.980 | 0.990 | 0.989 |
| StringTie-SR | 0.888 | 0.938 | 0.892 | 0.728 | 0.670 | 1.000 | 0.991 | 0.985 | 0.991 | 0.986 |
|  |  |  |  |  |  |  |  |  |  |  |
| Precision | One Splice Form | Two Splice Forms | Three Splice Forms | Four Splice Forms | Five Splice Forms | One Splice Form Annotated | Two Splice Forms Annotated | Three Splice Forms Annotated | Four Splice Forms Annotated | Five Splice Forms Annotated |
| CLASS | 0.802 | 0.296 | 0.131 | 0.137 | 0.112 | 0.994 | 0.988 | 0.980 | 0.954 | 0.964 |
| Cufflinks | 0.816 | 0.399 | 0.317 | 0.353 | 0.457 | 1.000 | 0.968 | 0.969 | 0.949 | 0.971 |
| FlipFlop | 0.737 | 0.205 | 0.118 | 0.152 | 0.123 | 1.000 | 0.971 | 0.956 | 0.944 | 0.943 |
| IReckon | 0.000 | 0.512 | 0.397 | 0.539 | 0.603 | 0.998 | 0.892 | 0.870 | 0.787 | 0.830 |
| IsoLasso | NA | 0.109 | 0.097 | 0.156 | 0.156 | 1.000 | 0.981 | 0.943 | 0.904 | 0.896 |
| MiTie | 0.860 | 0.552 | 0.329 | 0.134 | 0.092 | 0.988 | 0.427 | 0.453 | 0.427 | 0.457 |
| StringTie | 0.748 | 0.571 | 0.441 | 0.445 | 0.364 | 0.988 | 0.698 | 0.664 | 0.589 | 0.606 |
| StringTie-SR | 0.755 | 0.504 | 0.361 | 0.367 | 0.310 | 0.998 | 0.827 | 0.792 | 0.702 | 0.714 |
|  |  |  |  |  |  |  |  |  |  |  |
| **Right** | **Truncation** |  |  |  |  |  |  |  |  |  |
|  |  |  |  |  |  |  |  |  |  |  |
| Recall | One Splice Form | Two Splice Forms | Three Splice Forms | Four Splice Forms | Five Splice Forms | One Splice Form Annotated | Two Splice Forms Annotated | Three Splice Forms Annotated | Four Splice Forms Annotated | Five Splice Forms Annotated |
| CLASS | 0.890 | 0.492 | 0.316 | 0.221 | 0.132 | 1.000 | 0.490 | 0.325 | 0.253 | 0.214 |
| Cufflinks | 0.868 | 0.472 | 0.316 | 0.213 | 0.133 | 1.000 | 0.796 | 0.706 | 0.635 | 0.591 |
| FlipFlop | 0.938 | 0.508 | 0.336 | 0.251 | 0.164 | 1.000 | 0.500 | 0.343 | 0.268 | 0.232 |
| IReckon | 0.000 | 0.000 | 0.000 | 0.000 | 0.000 | 1.000 | 0.613 | 0.476 | 0.499 | 0.472 |
| IsoLasso | 0.000 | 0.000 | 0.000 | 0.029 | 0.014 | 1.000 | 0.536 | 0.348 | 0.273 | 0.226 |
| MiTie | 0.836 | 0.238 | 0.150 | 0.080 | 0.046 | 1.000 | 0.999 | 1.000 | 1.000 | 1.000 |
| StringTie | 0.892 | 0.496 | 0.320 | 0.223 | 0.134 | 1.000 | 0.977 | 0.968 | 0.980 | 0.983 |
| StringTie-SR | 0.888 | 0.502 | 0.334 | 0.227 | 0.139 | 1.000 | 0.964 | 0.961 | 0.976 | 0.975 |
|  |  |  |  |  |  |  |  |  |  |  |
| Precision | One Splice Form | Two Splice Forms | Three Splice Forms | Four Splice Forms | Five Splice Forms | One Splice Form Annotated | Two Splice Forms Annotated | Three Splice Forms Annotated | Four Splice Forms Annotated | Five Splice Forms Annotated |
| CLASS | 0.802 | 0.925 | 0.883 | 0.957 | 0.943 | 0.994 | 0.999 | 0.994 | 0.992 | 0.997 |
| Cufflinks | 0.816 | 0.933 | 0.952 | 0.973 | 0.978 | 1.000 | 0.884 | 0.890 | 0.847 | 0.883 |
| FlipFlop | 0.737 | 0.499 | 0.303 | 0.361 | 0.270 | 1.000 | 0.997 | 0.992 | 0.977 | 0.990 |
| IReckon | 0.000 | 0.000 | 0.000 | NA | NA | 0.998 | 0.723 | 0.753 | 0.740 | 0.789 |
| IsoLasso | NA | 0.000 | 0.000 | 0.453 | 0.350 | 1.000 | 0.953 | 0.961 | 0.933 | 0.967 |
| MiTie | 0.860 | 0.546 | 0.466 | 0.412 | 0.341 | 0.988 | 0.740 | 0.738 | 0.636 | 0.631 |
| StringTie | 0.748 | 0.905 | 0.865 | 0.921 | 0.944 | 0.988 | 0.430 | 0.470 | 0.443 | 0.494 |
| StringTie-SR | 0.755 | 0.937 | 0.898 | 0.904 | 0.933 | 0.998 | 0.432 | 0.471 | 0.446 | 0.495 |

Table S22 - Values used to generate figure S16

| **DataSet T1** |  |  |  |  |  |  |  |  |  |  |
| --- | --- | --- | --- | --- | --- | --- | --- | --- | --- | --- |
| **Figure S16** | **With Gene Models (STAR) - Split by annotation** | | | | | | |  |  | |
|  |  |  |  |  |  |  |  |  |  |  |
| **Left** | **Different exon starts/ends** | | | |  |  |  |  |  | |
|  |  |  |  |  |  |  |  |  |  |  |
| Recall | One Splice Form | Two Splice Forms | Three Splice Forms | Four Splice Forms | Five Splice Forms | One Splice Form Annotated | Two Splice Forms Annotated | Three Splice Forms Annotated | Four Splice Forms Annotated | Five Splice Forms Annotated |
| CLASS | 0.958 | 0.418 | 0.294 | 0.183 | 0.077 | 1.000 | 1.000 | 1.000 | 1.000 | 0.115 |
| Cufflinks | 0.916 | 0.258 | 0.192 | 0.119 | 0.072 | 1.000 | 0.620 | 0.480 | 0.393 | 0.327 |
| FlipFlop | 0.938 | 0.350 | 0.188 | 0.118 | 0.054 | 1.000 | 1.000 | 0.162 | 0.103 | 0.072 |
| IReckon | 0.000 | 0.454 | 0.342 | 0.264 | 0.140 | 1.000 | 0.626 | 0.343 | 0.251 | 0.232 |
| IsoLasso | 0.000 | 0.216 | 0.214 | 0.144 | 0.047 | 1.000 | 0.450 | 0.204 | 0.115 | 0.080 |
| MiTie | 0.892 | 0.210 | 0.138 | 0.071 | 0.032 | 1.000 | 1.000 | 1.000 | 0.995 | 0.993 |
| StringTie | 0.954 | 0.408 | 0.322 | 0.243 | 0.128 | 1.000 | 0.497 | 1.000 | 1.000 | 0.209 |
| StringTie-SR | 0.950 | 0.420 | 0.328 | 0.237 | 0.130 | 1.000 | 0.493 | 1.000 | 1.000 | 0.209 |
|  |  |  |  |  |  |  |  |  |  |  |
| Precision | One Splice Form | Two Splice Forms | Three Splice Forms | Four Splice Forms | Five Splice Forms | One Splice Form Annotated | Two Splice Forms Annotated | Three Splice Forms Annotated | Four Splice Forms Annotated | Five Splice Forms Annotated |
| CLASS | 0.822 | 0.235 | 0.088 | 0.077 | 0.027 | 0.952 | 0.443 | 0.252 | 0.160 | 0.998 |
| Cufflinks | 0.804 | 0.183 | 0.119 | 0.135 | 0.103 | 0.996 | 0.936 | 0.954 | 0.905 | 0.936 |
| FlipFlop | 0.526 | 0.053 | 0.017 | 0.017 | 0.007 | 0.938 | 0.378 | 0.998 | 0.994 | 0.997 |
| IReckon | 0.000 | 0.115 | 0.040 | 0.033 | 0.014 | 0.994 | 0.952 | 0.986 | 0.984 | 0.955 |
| IsoLasso | 0.000 | 0.099 | 0.071 | 0.092 | 0.029 | 0.970 | 0.991 | 0.996 | 0.994 | 0.997 |
| MiTie | 0.557 | 0.111 | 0.048 | 0.041 | 0.017 | 0.845 | 0.400 | 0.440 | 0.419 | 0.436 |
| StringTie | 0.764 | 0.147 | 0.080 | 0.088 | 0.043 | 0.972 | 0.999 | 0.316 | 0.240 | 0.980 |
| StringTie-SR | 0.783 | 0.169 | 0.089 | 0.091 | 0.046 | 0.962 | 0.999 | 0.314 | 0.240 | 0.980 |
|  |  |  |  |  |  |  |  |  |  |  |
| **Middle** | **Skipped Exons** | | | | |  |  |  |  |  |
|  |  |  |  |  |  |  |  |  |  |  |
| Recall | One Splice Form | Two Splice Forms | Three Splice Forms | Four Splice Forms | Five Splice Forms | One Splice Form Annotated | Two Splice Forms Annotated | Three Splice Forms Annotated | Four Splice Forms Annotated | Five Splice Forms Annotated |
| CLASS | 0.958 | 0.902 | 0.778 | 0.724 | 0.699 | 1.000 | 0.899 | 0.797 | 0.747 | 0.713 |
| Cufflinks | 0.916 | 0.526 | 0.338 | 0.269 | 0.278 | 1.000 | 0.959 | 0.898 | 0.898 | 0.872 |
| FlipFlop | 0.938 | 0.738 | 0.490 | 0.437 | 0.340 | 1.000 | 0.717 | 0.568 | 0.453 | 0.396 |
| IReckon | 0.000 | 0.852 | 0.710 | 0.612 | 0.616 | 1.000 | 0.909 | 0.925 | 0.916 | 0.932 |
| IsoLasso | 0.000 | 0.486 | 0.502 | 0.425 | 0.412 | 1.000 | 0.705 | 0.537 | 0.442 | 0.378 |
| MiTie | 0.892 | 0.474 | 0.268 | 0.139 | 0.073 | 1.000 | 1.000 | 1.000 | 1.000 | 1.000 |
| StringTie | 0.954 | 0.912 | 0.802 | 0.626 | 0.575 | 1.000 | 0.968 | 0.957 | 0.970 | 0.970 |
| StringTie-SR | 0.950 | 0.934 | 0.868 | 0.695 | 0.624 | 1.000 | 0.958 | 0.958 | 0.968 | 0.968 |
|  |  |  |  |  |  |  |  |  |  |  |
| Precision | One Splice Form | Two Splice Forms | Three Splice Forms | Four Splice Forms | Five Splice Forms | One Splice Form Annotated | Two Splice Forms Annotated | Three Splice Forms Annotated | Four Splice Forms Annotated | Five Splice Forms Annotated |
| CLASS | 0.822 | 0.176 | 0.086 | 0.107 | 0.086 | 0.952 | 0.989 | 0.975 | 0.956 | 0.959 |
| Cufflinks | 0.804 | 0.361 | 0.262 | 0.320 | 0.428 | 0.996 | 0.967 | 0.974 | 0.951 | 0.963 |
| FlipFlop | 0.526 | 0.121 | 0.059 | 0.076 | 0.051 | 0.938 | 0.995 | 0.983 | 0.973 | 0.973 |
| IReckon | 0.000 | 0.451 | 0.337 | 0.431 | 0.458 | 0.994 | 0.960 | 0.907 | 0.842 | 0.859 |
| IsoLasso | 0.000 | 0.171 | 0.130 | 0.186 | 0.177 | 0.970 | 0.988 | 0.973 | 0.957 | 0.942 |
| MiTie | 0.557 | 0.289 | 0.158 | 0.134 | 0.082 | 0.845 | 0.395 | 0.433 | 0.412 | 0.443 |
| StringTie | 0.764 | 0.307 | 0.220 | 0.217 | 0.181 | 0.972 | 0.978 | 0.947 | 0.904 | 0.888 |
| StringTie-SR | 0.783 | 0.408 | 0.276 | 0.277 | 0.217 | 0.962 | 0.978 | 0.945 | 0.906 | 0.890 |
|  |  |  |  |  |  |  |  |  |  |  |
| **Right** | **Truncation** |  |  |  |  |  |  |  |  |  |
|  |  |  |  |  |  |  |  |  |  |  |
| Recall | One Splice Form | Two Splice Forms | Three Splice Forms | Four Splice Forms | Five Splice Forms | One Splice Form Annotated | Two Splice Forms Annotated | Three Splice Forms Annotated | Four Splice Forms Annotated | Five Splice Forms Annotated |
| CLASS | 0.958 | 0.486 | 0.328 | 0.229 | 0.137 | 1.000 | 0.471 | 0.309 | 0.242 | 0.206 |
| Cufflinks | 0.916 | 0.444 | 0.316 | 0.193 | 0.123 | 1.000 | 0.747 | 0.666 | 0.573 | 0.546 |
| FlipFlop | 0.938 | 0.488 | 0.324 | 0.243 | 0.141 | 1.000 | 0.479 | 0.309 | 0.245 | 0.210 |
| IReckon | 0.000 | 0.000 | 0.000 | 0.000 | 0.000 | 1.000 | 0.615 | 0.492 | 0.500 | 0.474 |
| IsoLasso | 0.000 | 0.002 | 0.000 | 0.029 | 0.018 | 1.000 | 0.522 | 0.333 | 0.265 | 0.220 |
| MiTie | 0.892 | 0.290 | 0.214 | 0.124 | 0.072 | 1.000 | 0.999 | 1.000 | 1.000 | 1.000 |
| StringTie | 0.954 | 0.490 | 0.328 | 0.231 | 0.135 | 1.000 | 0.967 | 0.944 | 0.963 | 0.963 |
| StringTie-SR | 0.950 | 0.486 | 0.330 | 0.227 | 0.137 | 1.000 | 0.942 | 0.916 | 0.932 | 0.935 |
|  |  |  |  |  |  |  |  |  |  |  |
| Precision | One Splice Form | Two Splice Forms | Three Splice Forms | Four Splice Forms | Five Splice Forms | One Splice Form Annotated | Two Splice Forms Annotated | Three Splice Forms Annotated | Four Splice Forms Annotated | Five Splice Forms Annotated |
| CLASS | 0.822 | 0.769 | 0.672 | 0.779 | 0.685 | 0.952 | 0.999 | 0.987 | 0.999 | 0.992 |
| Cufflinks | 0.804 | 0.819 | 0.749 | 0.781 | 0.759 | 0.996 | 0.836 | 0.853 | 0.806 | 0.840 |
| FlipFlop | 0.526 | 0.254 | 0.142 | 0.154 | 0.081 | 0.938 | 0.986 | 0.961 | 0.970 | 0.982 |
| IReckon | 0.000 | 0.000 | 0.000 | 0.000 | 0.000 | 0.994 | 0.721 | 0.763 | 0.741 | 0.788 |
| IsoLasso | 0.000 | 0.006 | 0.000 | 0.116 | 0.061 | 0.970 | 0.950 | 0.954 | 0.929 | 0.964 |
| MiTie | 0.557 | 0.267 | 0.211 | 0.228 | 0.159 | 0.845 | 0.685 | 0.695 | 0.609 | 0.609 |
| StringTie | 0.764 | 0.666 | 0.465 | 0.606 | 0.491 | 0.972 | 0.428 | 0.469 | 0.441 | 0.494 |
| StringTie-SR | 0.783 | 0.700 | 0.511 | 0.641 | 0.557 | 0.962 | 0.432 | 0.469 | 0.442 | 0.494 |

Table S23 - Values to generate figure S17

| **DataSet EP and ER** | | |  |
| --- | --- | --- | --- |
| **Figure S17** | **With Gene Models (Truth)** | |  |
|  |  |  |  |
| **Left** | **EnsemblPerfect** |  |  |
|  |  |  |  |
| Recall | 0-10X | 10-100X | 100X_and_more |
| CLASS | 0.168 | 0.378 | 0.515 |
| Cufflinks | 0.295 | 0.586 | 0.887 |
| FlipFlop | 0.175 | 0.441 | 0.592 |
| IReckon | 0.574 | 0.699 | 0.772 |
| IsoLasso | 0.053 | 0.398 | 0.556 |
| MiTie | 0.602 | 0.719 | 0.805 |
| StringTie | 0.788 | 0.909 | 0.946 |
|  |  |  |  |
| Precision | 0-10X | 10-100X | 100X_and_more |
| CLASS | 0.506 | 0.602 | 0.564 |
| Cufflinks | 0.942 | 0.955 | 0.953 |
| FlipFlop | 0.456 | 0.564 | 0.518 |
| IReckon | 0.813 | 0.773 | 0.701 |
| IsoLasso | 0.277 | 0.647 | 0.614 |
| MiTie | 0.861 | 0.829 | 0.774 |
| StringTie | 0.901 | 0.871 | 0.808 |
|  |  |  |  |
| **Right** | **EnsemblRealistic** |  |  |
|  |  |  |  |
| Recall | 0-10X | 10-100X | 100X_and_more |
| CLASS | 0.161 | 0.380 | 0.536 |
| Cufflinks | 0.272 | 0.632 | 0.905 |
| FlipFlop | 0.089 | 0.245 | 0.257 |
| IReckon | 0.580 | 0.746 | 0.812 |
| IsoLasso | 0.034 | 0.281 | 0.498 |
| MiTie | 0.574 | 0.712 | 0.791 |
| StringTie | 0.723 | 0.897 | 0.950 |
|  |  |  |  |
| Precision | 0-10X | 10-100X | 100X_and_more |
| CLASS | 0.583 | 0.565 | 0.511 |
| Cufflinks | 0.806 | 0.818 | 0.789 |
| FlipFlop | 0.171 | 0.190 | 0.112 |
| IReckon | 0.607 | 0.451 | 0.335 |
| IsoLasso | 0.242 | 0.508 | 0.501 |
| MiTie | 0.866 | 0.768 | 0.663 |
| StringTie | 0.877 | 0.782 | 0.688 |

Table S24 - Values to genarate figure S18

| **DataSet EP and ER** | |  |  |  |  |  |
| --- | --- | --- | --- | --- | --- | --- |
| **Figure S18** | **With Gene Models (TopHat2) - Split by annotation** | | | | |  |
|  |  |  |  |  |  |  |
| **Left** | **EnsemblPerfect** | |  |  |  |  |
|  |  |  |  |  |  |  |
| Recall | 0-10X | 10-100X | 100X_and_more | 0-10X_annotated | 10-100X_annotated | 100X_and_more_annotated |
| CLASS | 0.113 | 0.309 | 0.445 | 0.159 | 0.375 | 0.512 |
| Cufflinks | 0.104 | 0.324 | 0.509 | 0.332 | 0.621 | 0.930 |
| FlipFlop | 0.016 | 0.066 | 0.121 | 0.221 | 0.501 | 0.659 |
| IReckon | 0.086 | 0.156 | 0.221 | 0.664 | 0.794 | 0.862 |
| IsoLasso | 0.016 | 0.079 | 0.139 | 0.083 | 0.454 | 0.625 |
| MiTie | 0.053 | 0.152 | 0.361 | 0.696 | 0.812 | 0.869 |
| StringTie | 0.121 | 0.362 | 0.574 | 0.912 | 0.989 | 0.994 |
| Stringtie-SR | 0.124 | 0.367 | 0.579 | 0.887 | 0.987 | 0.993 |
|  |  |  |  |  |  |  |
| Precision | 0-10X | 10-100X | 100X_and_more | 0-10X_annotated | 10-100X_annotated | 100X_and_more_annotated |
| CLASS | 0.410 | 0.524 | 0.558 | 0.482 | 0.600 | 0.554 |
| Cufflinks | 0.847 | 0.900 | 0.894 | 0.925 | 0.950 | 0.945 |
| FlipFlop | 0.077 | 0.150 | 0.209 | 0.499 | 0.597 | 0.536 |
| IReckon | 0.435 | 0.419 | 0.446 | 0.830 | 0.801 | 0.721 |
| IsoLasso | 0.119 | 0.253 | 0.320 | 0.367 | 0.673 | 0.631 |
| MiTie | 0.412 | 0.474 | 0.635 | 0.869 | 0.844 | 0.778 |
| StringTie | 0.589 | 0.692 | 0.719 | 0.902 | 0.873 | 0.802 |
| Stringtie-SR | 0.585 | 0.698 | 0.716 | 0.900 | 0.870 | 0.797 |
|  |  |  |  |  |  |  |
| **Right** | **EnsemblRealistic** | |  |  |  |  |
|  |  |  |  |  |  |  |
| Recall | 0-10X | 10-100X | 100X_and_more | 0-10X_annotated | 10-100X_annotated | 100X_and_more_annotated |
| CLASS | 0.109 | 0.317 | 0.494 | 0.166 | 0.391 | 0.562 |
| Cufflinks | 0.079 | 0.321 | 0.523 | 0.310 | 0.679 | 0.967 |
| FlipFlop | 0.013 | 0.031 | 0.037 | 0.107 | 0.274 | 0.290 |
| IReckon | 0.072 | 0.160 | 0.216 | 0.594 | 0.775 | 0.875 |
| IsoLasso | 0.012 | 0.059 | 0.092 | 0.048 | 0.317 | 0.564 |
| MiTie | 0.023 | 0.092 | 0.261 | 0.676 | 0.814 | 0.871 |
| StringTie | 0.090 | 0.365 | 0.617 | 0.862 | 0.975 | 0.992 |
| Stringtie-SR | 0.085 | 0.365 | 0.610 | 0.831 | 0.970 | 0.991 |
|  |  |  |  |  |  |  |
| Precision | 0-10X | 10-100X | 100X_and_more | 0-10X_annotated | 10-100X_annotated | 100X_and_more_annotated |
| CLASS | 0.457 | 0.510 | 0.529 | 0.596 | 0.577 | 0.523 |
| Cufflinks | 0.542 | 0.677 | 0.716 | 0.823 | 0.824 | 0.787 |
| FlipFlop | 0.027 | 0.031 | 0.023 | 0.205 | 0.206 | 0.122 |
| IReckon | 0.243 | 0.250 | 0.250 | 0.760 | 0.622 | 0.502 |
| IsoLasso | 0.088 | 0.185 | 0.196 | 0.305 | 0.528 | 0.505 |
| MiTie | 0.207 | 0.288 | 0.449 | 0.888 | 0.791 | 0.679 |
| StringTie | 0.443 | 0.576 | 0.631 | 0.889 | 0.789 | 0.684 |
| Stringtie-SR | 0.414 | 0.558 | 0.620 | 0.883 | 0.776 | 0.670 |

Table S25 - Values to generate figure S19

| **DataSet EP and ER** | |  |  |  |  |  |
| --- | --- | --- | --- | --- | --- | --- |
| **Figure S19** | **With Gene Models (STAR) - Split by annotation** | | | | |  |
|  |  |  |  |  |  |  |
| **Left** | **EnsemblPerfect** | |  |  |  |  |
|  |  |  |  |  |  |  |
| Recall | 0-10X | 10-100X | 100X_and_more | 0-10X_annotated | 10-100X_annotated | 100X_and_more_annotated |
| CLASS | 0.100 | 0.247 | 0.378 | 0.108 | 0.257 | 0.358 |
| Cufflinks | 0.116 | 0.325 | 0.514 | 0.311 | 0.597 | 0.921 |
| FlipFlop | 0.016 | 0.049 | 0.094 | 0.146 | 0.356 | 0.510 |
| IReckon | 0.098 | 0.167 | 0.235 | 0.655 | 0.785 | 0.858 |
| IsoLasso | 0.018 | 0.077 | 0.125 | 0.067 | 0.415 | 0.588 |
| MiTie | 0.066 | 0.169 | 0.408 | 0.669 | 0.794 | 0.860 |
| StringTie | 0.123 | 0.369 | 0.622 | 0.816 | 0.918 | 0.924 |
| Stringtie-SR | 0.130 | 0.371 | 0.599 | 0.779 | 0.908 | 0.918 |
|  |  |  |  |  |  |  |
| Precision | 0-10X | 10-100X | 100X_and_more | 0-10X_annotated | 10-100X_annotated | 100X_and_more_annotated |
| CLASS | 0.431 | 0.506 | 0.541 | 0.418 | 0.533 | 0.497 |
| Cufflinks | 0.797 | 0.861 | 0.900 | 0.905 | 0.932 | 0.932 |
| FlipFlop | 0.061 | 0.104 | 0.153 | 0.347 | 0.467 | 0.417 |
| IReckon | 0.463 | 0.424 | 0.444 | 0.817 | 0.787 | 0.708 |
| IsoLasso | 0.116 | 0.236 | 0.286 | 0.300 | 0.639 | 0.601 |
| MiTie | 0.368 | 0.393 | 0.542 | 0.804 | 0.769 | 0.681 |
| StringTie | 0.557 | 0.669 | 0.734 | 0.874 | 0.841 | 0.755 |
| Stringtie-SR | 0.582 | 0.678 | 0.721 | 0.864 | 0.840 | 0.757 |
|  |  |  |  |  |  |  |
| **Right** | **EnsemblRealistic** | |  |  |  |  |
|  |  |  |  |  |  |  |
| Recall | 0-10X | 10-100X | 100X_and_more | 0-10X_annotated | 10-100X_annotated | 100X_and_more_annotated |
| CLASS | 0.102 | 0.286 | 0.440 | 0.116 | 0.296 | 0.414 |
| Cufflinks | 0.076 | 0.283 | 0.412 | 0.283 | 0.632 | 0.942 |
| FlipFlop | 0.017 | 0.025 | 0.029 | 0.085 | 0.200 | 0.220 |
| IReckon | 0.087 | 0.170 | 0.220 | 0.580 | 0.760 | 0.860 |
| IsoLasso | 0.012 | 0.056 | 0.078 | 0.040 | 0.262 | 0.478 |
| MiTie | 0.028 | 0.099 | 0.274 | 0.666 | 0.810 | 0.874 |
| StringTie | 0.083 | 0.348 | 0.603 | 0.715 | 0.843 | 0.823 |
| Stringtie-SR | 0.092 | 0.359 | 0.589 | 0.680 | 0.829 | 0.812 |
|  |  |  |  |  |  |  |
| Precision | 0-10X | 10-100X | 100X_and_more | 0-10X_annotated | 10-100X_annotated | 100X_and_more_annotated |
| CLASS | 0.406 | 0.476 | 0.467 | 0.479 | 0.492 | 0.424 |
| Cufflinks | 0.486 | 0.611 | 0.614 | 0.788 | 0.793 | 0.756 |
| FlipFlop | 0.033 | 0.023 | 0.018 | 0.167 | 0.154 | 0.091 |
| IReckon | 0.271 | 0.255 | 0.233 | 0.739 | 0.590 | 0.466 |
| IsoLasso | 0.077 | 0.159 | 0.158 | 0.251 | 0.463 | 0.445 |
| MiTie | 0.182 | 0.236 | 0.362 | 0.850 | 0.730 | 0.599 |
| StringTie | 0.333 | 0.490 | 0.532 | 0.833 | 0.697 | 0.551 |
| Stringtie-SR | 0.345 | 0.499 | 0.554 | 0.823 | 0.696 | 0.550 |
